# Development of Reference Transcriptomes for the Major Field Insect Pests of Cowpea: A Toolbox for Insect Pest Management Approaches in West Africa

**DOI:** 10.1371/journal.pone.0079929

**Published:** 2013-11-22

**Authors:** Tolulope A. Agunbiade, Weilin Sun, Brad S. Coates, Rousseau Djouaka, Manuele Tamò, Malick N. Ba, Clementine Binso-Dabire, Ibrahim Baoua, Brett P. Olds, Barry R. Pittendrigh

**Affiliations:** 1 Department of Entomology, University of Illinois at Urbana-Champaign, Urbana, Illinois, United States of America; 2 Corn Insects and Crop Genetics Research Unit, United States Department of Agriculture, Agricultural Research Service, Ames, Iowa, United States of America; 3 International Institute of Tropical Agriculture, Cotonou, Benin; 4 Institut de l’Environnement et de Recherches Agricoles, Ouagadougou, Burkina Faso; 5 Institut National de la Recherche Agronomique du Niger, Maradi, Niger; 6 Department of Animal Biology, University of Illinois at Urbana-Champaign, Urbana, Illinois, United States of America; University of North Carolina at Charlotte, United States of America

## Abstract

Cowpea is a widely cultivated and major nutritional source of protein for many people that live in West Africa. Annual yields and longevity of grain storage is greatly reduced by feeding damage caused by a complex of insect pests that include the pod sucking bugs, *Anoplocnemis curvipes* Fabricius (Hemiptera: Coreidae) and *Clavigralla tomentosicollis* Stål (Hemiptera: Coreidae); as well as phloem-feeding cowpea aphids, *Aphis craccivora* Koch (Hemiptera: Aphididae) and flower thrips, *Megalurothrips sjostedti* Trybom (Thysanoptera: Thripidae). Efforts to control these pests remain a challenge and there is a need to understand the structure and movement of these pest populations in order to facilitate the development of integrated pest management strategies (IPM). Molecular tools have the potential to help facilitate a better understanding of pest populations. Towards this goal, we used 454 pyrosequencing technology to generate 319,126, 176,262, 320,722 and 227,882 raw reads from *A. curvipes*, *A. craccivora*, *C. tomentosicollis* and *M. sjostedti*, respectively. The reads were *de novo* assembled into 11,687, 7,647, 10,652 and 7,348 transcripts for *A. curvipes*, *A. craccivora*, *C. tomentosicollis* and *M. sjostedti*, respectively. Functional annotation of the resulting transcripts identified genes putatively involved in insecticide resistance, pathogen defense and immunity. Additionally, sequences that matched the primary aphid endosymbiont, *Buchnera aphidicola*, were identified among *A. craccivora* transcripts. Furthermore, 742, 97, 607 and 180 single nucleotide polymorphisms (SNPs) were respectively predicted among *A. curvipes*, *A. craccivora*, *C. tomentosicollis* and *M. sjostedti* transcripts, and will likely be valuable tools for future molecular genetic marker development. These results demonstrate that Roche 454-based transcriptome sequencing could be useful for the development of genomic resources for cowpea pest insects in West Africa.

## Introduction

Crops of cowpea (*Vigna Unguiculata* (L). Walp) provide a major nutritional source of protein for about 200 million people in sub-Saharan Africa [1]. Cowpea production is highest in the West African countries of Nigeria, Niger and Burkina Faso, where insect feeding damage by over 100 pest species is a major constraint on field production and in grain storage [1]. Yield is most dramatically affected by insect pests that occur during the flowering and seed pod stages. These include flower and pod feeding insects such as flower thrips, *Megalurothrips sjostedti* Trybom (Thysanoptera: Thripidae); legume pod borer, *Maruca vitrata* Fabricius (Lepidoptera: Crambidae); pod sucking insects, *Clavigralla tomentosicollis* Stål (Hemiptera: Coreidae) [2] and *Anoplocnemis curvipes* Fabricius (Hemiptera: Coreidae); and phloem-feeding cowpea aphids, *Aphis craccivora* Koch (Hemiptera: Aphididae). Crop damage by these insect pests can be as high as 60 to 100% in the field [3–5]. *Aphis craccivora* can cause significant damage even at low population densities due to its ability to transmit at least 14 viruses including the potyviruses, the cowpea aphid-borne mosaic virus (CABMV) [6,7] and the blackeye cowpea mosaic virus (BICMV) [8]. These viruses produce severe cowpea mottling, chlorosis, and seed shriveling [9] which severely reduce yields [10,11]. In contrast to most plant viruses, which fail to cross into developing embryos from infected maternal tissues [12], CABMV and BICMV appear to propagate via vertical transmission from parent to progeny seed [13,14] and is exacerbated by horizontal transmissions by aphid vectors. 

Much research has been directed towards developing strategies to control the feeding of the legume pod borer, *M. vitrata* on cowpea crops. Past use of chemical insecticides has resulted in increased frequencies of resistance in *M. vitrata* to three classes of insecticides in Nigeria [15], and unfortunately fits within the paradigm where selection pressures imposed by widespread application of a chemical control agent can oftentimes lead to the evolution of insecticide resistance within targeted pest insect populations [16,17]. Additionally, chemical insecticides are often financially inaccessible to smallholder farmers in West Africa, and pose serious health and environmental risks when used indiscriminately by untrained applicators [18,19]. Therefore, recent shifts toward the use of affordable and sustainable biocontrol measures have been initiated within West Africa [2,20]. Some of these potential *M. vitrata* control strategies have included the deployment of bio-pesticides [21,22] and the development of a transgenic cowpea that expresses *Bacillus thuringiensis* (*Bt*) toxins [23]. Additionally, traps baited with the female *M. vitrata* sex pheromone blends, in a 100:5:5 ratio of (*E*, *E*)-10,12-hexadecadienal, (*E*, *E*)-10,12-hexadecadienol and (*E*)-10-hexadecenal, have been distributed to farmers in Benin and used as a successful early warning tool [24]. These baited traps have the potential for monitoring seasonal northward *M. vitrata* migrations during the rainy season, but the fidelity of pheromone blend, and trap position and design can affect the accuracy of resulting estimates of population size and route of migration [25–27]. The population genetic structure of *M. vitrata* has been described through the application of next generation sequencing (NGS) and high throughput single nucleotide polymorphism (SNP) genotyping technologies [28] as well as microsatellite loci [29]. In these aforementioned studies, *M. vitrata* population structure and estimates of gene flow (migration) within West African cowpea production area were assessed. The results of these studies have the potential to enhance integrated pest management (IPM) programs to determine the logical locations of natural enemy releases. This prior research on *M. vitrata* serves as a model for the application of genome-based approaches to increase the effectiveness of strategies used to control pest insect populations. 

Since transgenic cowpea that express *Bacillus thuringiensis* (*Bt*) Cry1Ab toxin shows no toxicity towards non-lepidopteran insects and a majority of biocontrol strategies for *M. vitrata* are species-specific, cowpea crops remain susceptible to continued feeding and plant disease transmission by thrips, aphids and pod sucking pests. Thus, the only method widely available to date for the control of *A. craccivora*, *A. curvipes*, *C. tomentosicollis* and *M. sjostedti* by indigenous farmers in West Africa has been the application of chemical insecticides. Since insecticide resistance had evolved within *M*. *vitrata* populations [15], the establishment of effective insect resistance management (IRM) plans for *A. craccivora*, *A. curvipes*, *C. tomentosicollis* and *M. sjostedti* may be critical for delaying the evolution of resistance. The absence of population genetic data for these species hinders the estimation of movement patterns of these insects within their endemic range such that the regional scales necessary for IRM programs to remain effective are difficult to devise. NGS technologies that include Roche 454 GS FLX, Solexa/Illumina Genome Analyzer, ABI/SOLiD Gene Sequencers and Helicos Genetic Analysis System platforms use massively parallel pyrosequencing technologies to collect millions of nucleotide sequences in very short time frames [30-34]. Moreover, NGS technologies provide a rapid and cost-effective way to obtain large amounts of DNA sequence data from organisms where no prior information had existed [28]. *De novo* transcriptome analysis has proven to be a valuable first step to obtaining sequence information and expression levels of genes involved in developmental and metabolic pathways, insecticide resistance, and to discover single nucleotide polymorphisms (SNPs) in all kinds of model and non-model organisms [35-38]. SNPs are changes of a single nucleotide at a specific location within the genome of a species, and high-throughput assays have been developed for their detection and application as genetic markers [39-43]. Estimation of allelic frequency variation at SNP loci are effective for describing population demographics [44] and are increasingly becoming the marker of choice in population genetic analysis. 

In this study, we applied Roche 454 sequencing technology to generate and subsequently assemble contigs from DNA sequencing reads from independent normalized cDNA libraries for *A. curvipes*, *A. craccivora*, *C. tomentosicollis* and *M. sjostedti*. Annotations of individual gene transcripts were used to identify candidate genes putatively involved in insecticide resistance, regulation of insect growth and response to disease transmission. This is the first report of genomic data for these insect pests and provides valuable tool for understanding molecular gene functions of several major field insect pests in cowpea cropping systems of West Africa. The application of this genomics data might ultimately lead to a better understanding of the pest populations, with the long-term potential to improve the effectiveness of IPM programs by better defining pest-pathogen interactions, and pest population dynamics prior to deployment of biocontrol agents.

## Materials and Methods

### Ethics Statement

For all the insect samples used in the study, no permission was required for the insect sampling and collection. Insect sampling and collection in Benin was performed with our collaborators at the International Institute of Tropical Agriculture (IITA). Permission was not required because the insects used for the study are common insect pests on legumes, and IITA Benin has a Memorandum of Understanding (MOU) with the government of Benin for conducting research on these insect pests. In Burkina Faso and Niger, insect sampling and collection were also carried out with our collaborators at the Institut de l’Environnement et de Recherches Agricoles (INERA) and the Institut National de la Recherche Agronomique du Niger (INRAN), respectively. Both INERA and INRAN are national government agencies in their respective countries and therefore have the mandate to work on these insect pests from their respective governments. The insects used for this study are not endangered species.

### Development of Reference Transcriptome Sequence Assemblies

**Figure 1:**
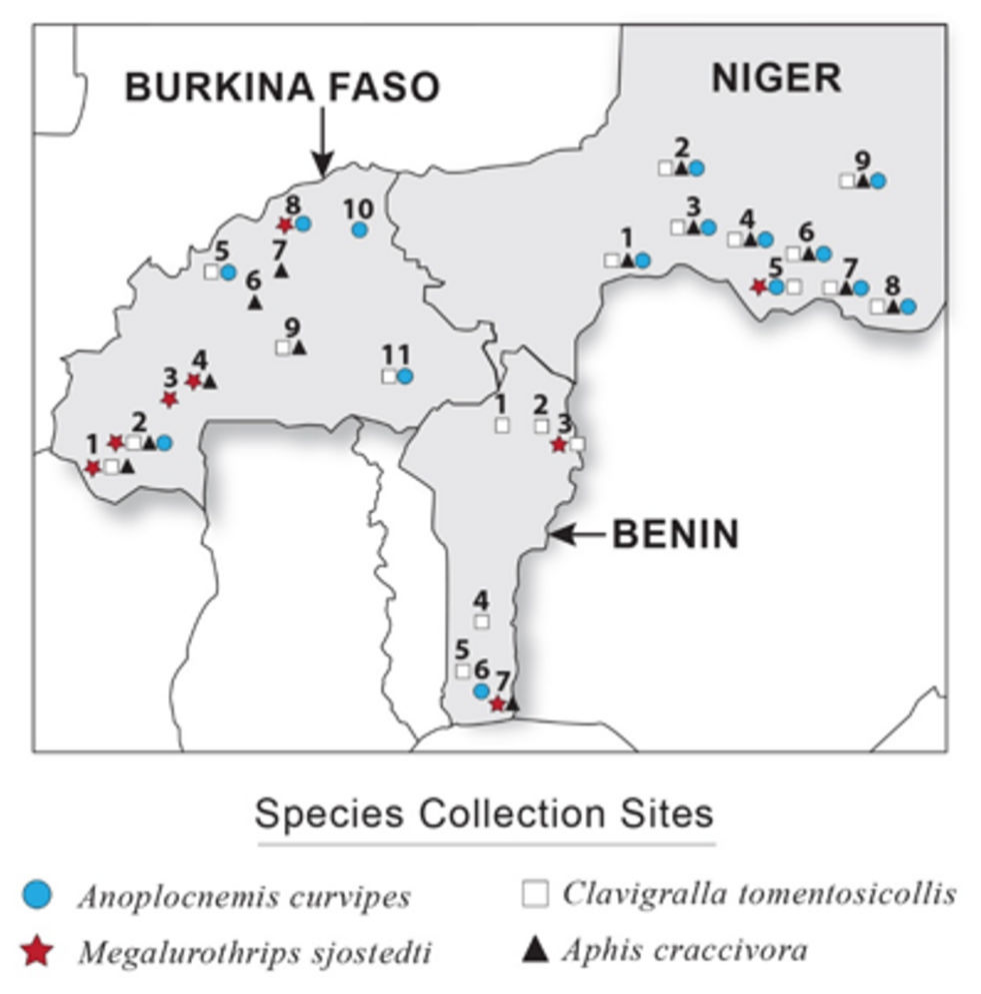
Map of Benin, Burkina Faso, and Niger showing the sites from which *A. curvipes*, *A. craccivora*, *C. tomentosicollis* and *M. sjostedti* were collected.

Insect samples were collected during the summer through fall of 2011 at 7, 11 and 9 locations in Benin, Burkina Faso and Niger respectively for *A. craccivora*, *A. curvipes*, *C. tomentosicollis* and *M. sjostedti* (Figure 1). A total of 79, 1,920, 364 and 740 individual insect samples were collected respectively for *A. craccivora*, *A. curvipes*, *C. tomentosicollis* and *M. sjostedti* from these locations. Both the larval and adult life stages were sampled for all species and stored in RNAlater (Ambion, TX, USA) immediately after collection in the field. All samples from each species, from a single location, were pooled and total RNA was extracted from the insect samples at IITA Benin and INERA Burkina Faso using QIAGEN RNeasy RNA extraction kits (CA, USA) and following the manufacturer's instructions. The RNA was shipped to University of Illinois at Urbana-Champaign (UIUC), USA in 70% ethanol where it was resuspended in water and quantified by measuring the absorbance at 260 nm using a NanoDrop spectrophotometer (Thermo Scientific, DE, USA). The samples were then stored in an ultra-low temperature freezer (-80°C). 

Four normalized cDNA libraries were constructed and sequenced on a Roche 454 GS-FLX at the W.M. Keck Center for Comparative and Functional Genomics, Roy J. Carver Biotechnology Center, UIUC. Briefly, messenger RNA (mRNA) was isolated from 10μg of total RNA with the Oligotex kit (Qiagen, Valencia, CA). The mRNA-enriched fraction was converted to 454 barcoded cDNA libraries and normalized [45]. The barcoded libraries were pooled in equimolar concentration based on average fragment length and concentration. After library construction, the pooled libraries were quantified using a Qubit fluorometer (Invitrogen, CA, USA) and average fragment sizes were determined by analyzing 1µl of the samples on the Bioanalyzer (Agilent, CA, USA) using a DNA 7500 chip. The pooled library was diluted to 1 x 10^6^ molecules/µl. Emulsion-based clonal amplification and sequencing on a full plate on the 454 Genome Sequencer FLX+ system was performed according to the manufacturer’s instructions (454 Life Sciences, CT, USA). Signal processing and base calling were performed using the bundled 454 Data Analysis Software v2.6. 

The raw sequence read data from the four insect pests were analyzed using the CLC Genomics Workbench 6.0.1 (Cambridge, MA, USA). Pre-processing of the raw reads from each of the four insect samples involved trimming each 454 read using a Phred quality score of 20 and also removing nucleotides < 50 bp from the ends. The adapter sequences were also trimmed from the raw reads. The processed read data from each of the four insect samples were assembled into contiguous sequences using parameters: mismatch cost = 2, insertion and deletion cost = 3, length fraction = 60% and similarity = 90%. After assembly, the vector contamination were removed using the UniVec database and also after assembly, human, bacterial, fish (*Danio rerio*), mouse (*Mus musculus*), *Salmonella enterica*, archeal and viral contamination were removed using a web-based version of DeConSeq [46] using a coverage of 90% and a sequence identity threshold of 94%. The clean transcriptomes, with the contaminations removed, were then deposited at DDBJ/EMBL/GenBank for each of the four insect species. 

### Functional Gene Annotation

Open reading frames (ORFs) were predicted from assembled contigs using the ORF-Predictor server [47] using all 6 possible reading frames for prediction. The assembled transcripts were used as queries to search against NCBI’s non-redundant (nr) database using the BLASTx algorithm [48], with a cut-off *E*-value of ≤ 10^-6^ and a high scoring segment pairs (HSP) length cut-off of 33. The Blast2GO software package v2.6.5 [49] was used for automating BLASTx searches as well as to retrieve associated gene ontology (GO) terms that allowed the prediction of transcript functions [49,50]. The contigs with significant GO terms were determined with an *E*-value hit filter of ≤ 1 x 10^-6^ and an annotation cut off of 55. Gene ontologies were categorized with respect to molecular function, biological process and cellular component. 

Annotations for *A. craccivora*, *A. curvipes*, *C. tomentosicollis* and *M. sjostedti* gene functions were manually searched for those putatively involved in the expression of insecticide resistance traits, and pathogen defense and immunity, using each gene function as keywords to search GO terms. Prediction of candidate gene function was also obtained using InterProScan [51,52] and Kyoto Encyclodepia of Genes and Genomes (KEGG) pathway analyses [53] using Blast2GO v2.6.5 [49]. 

### Prediction of Putative SNPs

The associated SNP detection software on the CLC Workbench 6.0.1 was used for putative SNP discovery among Roche 454 reads for all species. Attempts to reduce the rate of false SNP discovery included applying a read coverage cut-off of ≥ 35-fold and reporting SNPs that were present in ≥ 35% of the aligned reads. These criteria might reduce the false SNP discovery rate by potentially eliminating sequencing errors from the prediction. However, such stringent criteria likely increases type II error, therefore we also performed a comparative prediction of putative SNPs using a reduced coverage cut off of ≥ 10%. All putative indels and nucleotide variants involving > 2 nucleotides were excluded. Lastly, only SNPs located in an ORF were extracted and reported in this study. We checked whether SNPs introduced an amino acid change to differentiate non-synonymous and synonymous SNPs by using the open reading frames of each of the contigs with SNPs, identifying the codons containing the SNPs and then translating and comparing the amino acids for each allele on CLC Workbench 6.0.1. We also checked the type of substitution, whether transition or transversion, using the CLC Workbench 6.0.1. 

### Metagenomic Identification of Endosymbiont and Pathogen Transcripts

The bacterial endosymbiont, *Buchnera aphidicola*, was identified from a BLASTn search against *Buchnera* (Taxid: 32199) in NCBI using the assembled *A. craccivora* contigs as queries. The *A. craccivora* contigs with relevant hits to *Buchnera* were extracted and further confirmatory analysis was performed using InterProScan on Blast2GO v2.6.5, and the associated KEGG pathways were investigated also using Blast2GO v2.6.5. 

### Data Deposition

The raw Roche 454 sequence data were submitted to the National Center for Biotechnology Information (NCBI) Sequence Read Archive (SRA) with accession numbers of SRR768514, SRR768515, SRR768524 and SRR768525 for *A. curvipes*, *A. craccivora*, *C. tomentosicollis* and *M. sjostedti*, respectively. The Transcriptome Shotgun Assembly project for the four insect species were submitted to DDBJ/EMBL/GenBank under the accession numbers of GAJV00000000, GAJW00000000, GAJX00000000 and GAJY00000000 for *A. curvipes*, *A. craccivora*, *C. tomentosicollis* and *M. sjostedti*, respectively. The version of the TSA accession numbers described in this paper is the first version for each of the four species. 

## Results

### Development of Reference Transcriptome Sequence Assemblies

Normalized species-specific libraries were successfully constructed from mRNA isolated from pooled samples of all tissues and pooled adult and larval life stages. A total of 319,126, 176,262, 320,722 and 227,882 raw reads were respectively obtained from these *A. curvipes*, *A. craccivora*, *C. tomentosicollis* and *M. sjostedti* libraries. The bacterial and human contaminants discovered by DeConSeq were negligible across the four insect species. Seventeen, 2, 11 and 37 contigs were identified by DeConSeq as contaminants in *A. curvipes*, *A. craccivora*, *C. tomentosicollis* and *M. sjostedti*, respectively and were also subsequently removed from the transcripts. The remaining statistics for sequence and contig assemblies are reported in Table 1. After assembly and decontamination, the mean contig length ranged from 669.8 to 688.1, from which ORFs with a mean length of between 498.5 and 524.5 bp was predicted (Table 1). 

**Table 1:** Statistics from Roche 454 sequencing of *A. curvipes*, *A. craccivora*, *C. tomentosicollis* and *M. sjostedti* cDNA libraries generated from pools of all tissues and the larval and adult life stages.

	*A. curvipes*	*A. craccivora*	*C. tomentosicollis*	*M. sjostedti*
Putative SNPs identified	742	97	607	180
Contigs with SNPs	256	30	225	63
Transition	505	65	423	116
Transversion	237	32	184	64
Transition/Transversion Ratio	2.1	2.0	2.3	1.8
SNPs per Kilobase	0.09	0.02	0.08	0.04
Synonymous SNPs	425	72	419	97
Non-synonymous SNPs	317	25	188	83
Mean read depth of SNPs	97.5	66.6	115.5	74.9

### Functional Gene Annotation

**Figure 2:**
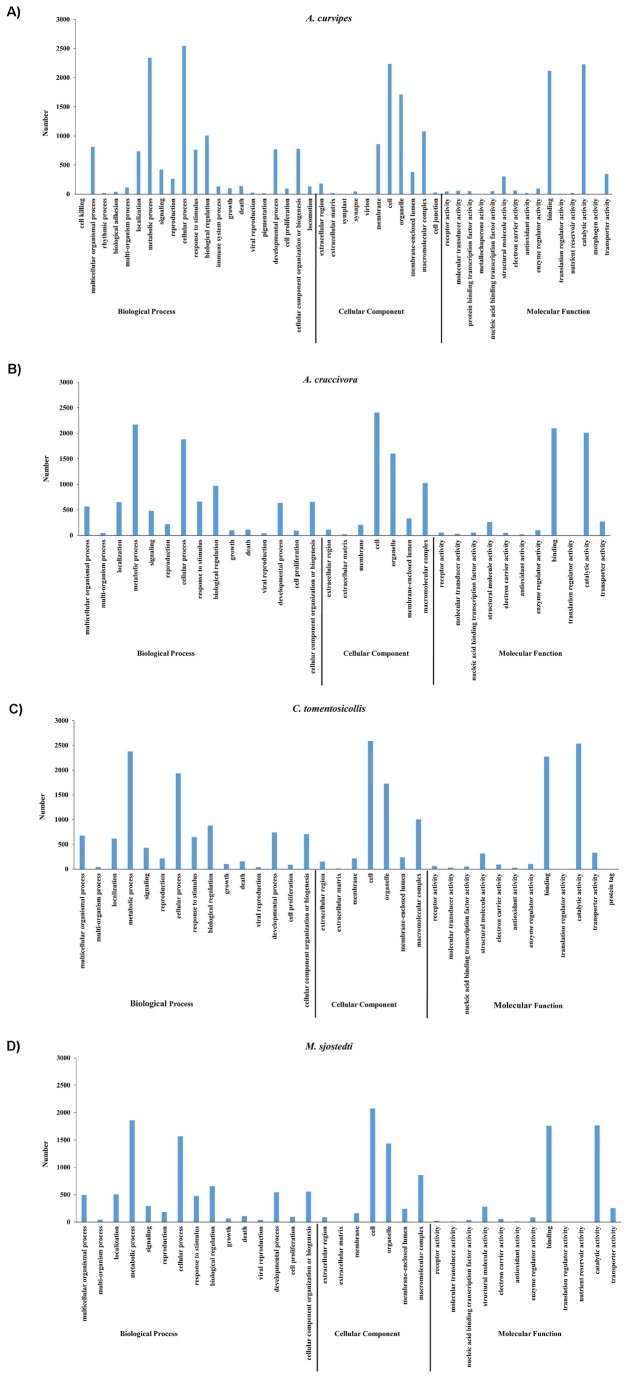
Gene ontology classification into biological process, cellular component and molecular function. Gene ontology terms were determined using an e-value of ≤ 1.0 e-6 sorted based on level 2 classifications in all the contigs of (a) *A. curvipes*, (b) *A. craccivora*, (c) *C. tomentosicollis*, (d) *M. sjostedti*.

ORFs were predicted from ≥ 98% of *A. curvipes*, *A. craccivora*, *C. tomentosicollis* and *M. sjostedti* transcripts, and respectively showed mean lengths of 498.5, 514.3, 524.5 and 508.8 bp (Table 1). Blast2GO output indicated that BLASTx hits were obtained for 6,430 (55%), 7,647 (79.9%), 6,839 (64.2%) and 4,292 (58.4%) contigs in *A. curvipes*, *A. craccivora*, *C. tomentosicollis* and *M. sjostedti*, respectively. The contigs with significant BLASTx matches were assigned GO terms into molecular function, biological process, and cellular components (Tables S1a to S1d). The functional classification based on molecular function, biological process and cellular component are represented in Figures 2a to 2d. In the molecular function category, the most highly represented were assigned to binding (13.7% for *A. curvipes*, 13.6% for *A. craccivora*, 13.4% for *C. tomentosicollis* and 13.5% for *M. sjostedti*) and catalytic activity (14.4% for *A. curvipes*, 13.1% for *A. craccivora*, 14.9% for *C. tomentosicollis* and 13.5% for *M. sjostedti*). In the biological process category, the most highly represented were assigned to cellular process (4.7% for *A. curvipes*, 4.7% for *A. craccivora*, 4.7% for *C. tomentosicollis* and 4.7% for *M. sjostedti*) and metabolic process (5.4% for *A. curvipes*, 5.4% for *A. craccivora*, 5.8% for *C. tomentosicollis* and 5.6% for *M. sjostedti*) while in the cellular component category, the most highly represented were assigned to cell (9.3% for *A. curvipes*, 9.1% for *A. craccivora*, 9.5% for *C. tomentosicollis* and 9% for *M*. *sjostedti*) and cell part (8.2% for *A. curvipes*, 8% for *A. craccivora*, 8.3% for *C*. *tomentosicollis* and 8.2% for *M. sjostedti*) (Tables S1a to S1d).

The majority of the top BLASTx hits in the four insect species were from insects. In *A. curvipes*, the most common were from Hemiptera [*Riptortus pedestris* (23.6%)], Coleoptera [*Tribolium castaneum* (7.1%)], Phthiraptera [*Pediculus humanus* (6.7%)] and another Hemiptera [*Acryrthosiphon pisum* (6%)] (Figure S1a). The most frequent hits from *A. craccivora* were from Hemiptera [*A. pisum* (83.7%)], two fungi species [*Rhizopus delemar* (1.5%); *Batrachochytrium dendrobatidis* (0.7%)] and Phthiraptera [*P. humanus* (0.5%)] (Figure S1b). The most frequent in *C. tomentosicollis* were from Hemiptera [*R. pedestris* (27.7%)], Coleoptera [*T. castaneum* (7.7%)], another Hemiptera [*A. pisum* (7%)] and Phthiraptera [*P. humanus* (6.7%)] and (Figure S1c) while the most frequent in *M. sjostedti* transcripts were from Coleoptera [*T. castaneum* (9.7%)], Phthiraptera [*P. humanus* (8.4%)], Lepidoptera [*Danaus plexippus* (6.5%)] and Hymenoptera [*Nasonia vitripennis* (5.6%)] (Figure S1d). BLASTx hits also revealed matches to other fungi species, bacteria, and a plant among *A. craccivora* contigs (Figure S1b), but these were not analogously observed within libraries from the other three insects. 

**Figure 3:**
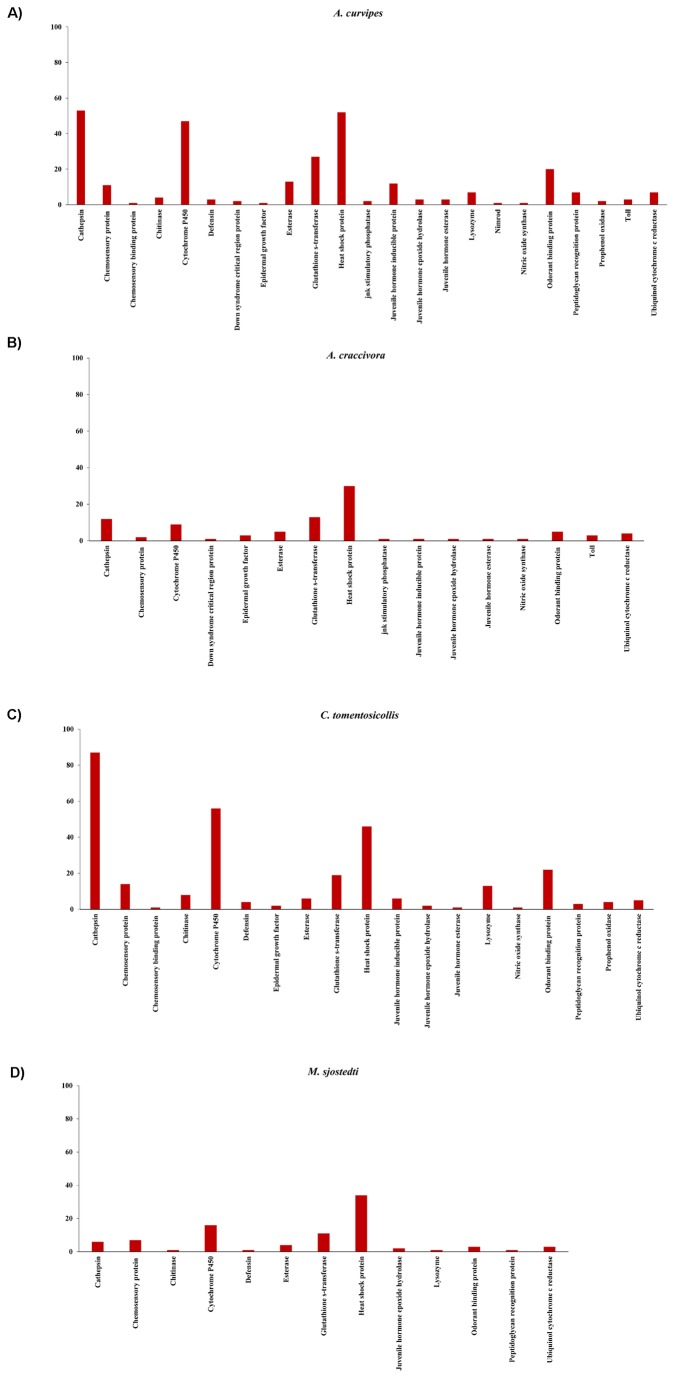
Transcripts putatively involved in responses to xenobiotic (e.g., insecticide resistance) and disease transmission in (a) *A. curvipes*, (b) *A. craccivora*, (c) *C. tomentosicollis*, (d) *M. sjostedti*.

 Within the libraries constructed from *A. curvipes*, *A. craccivora*, *C. tomentosicollis* and *M. sjostedti* cDNA, a combined total of 23 candidate genes for detoxification, immunity and pathogen defense, development and communication were identified including cytochrome P450, glutathione s-transferase, esterase, cathepsin, heat shock protein, chitinase, defensin, c-Jun NH (2)-terminal kinase (jnk) stimulatory phosphatase, down syndrome critical region protein, epidermal growth factor, lysozyme, nimrod, nitric acid synthase, prophenol oxidase, ubiquinol cytochrome c reductase, peptidoglycan recognition protein, toll protein, chemosensory protein, juvenile hormone inducible protein, juvenile hormone esterase and juvenile hormone epoxide hydrolase, chemosensory binding protein as well as odorant binding protein (Figures 3a to 3d).

### Metagenomic Identification of Endosymbiont- and Pathogen-Derived Transcripts

Thirty six BLASTn hits to the NCBI nr protein database were identical to the primary endosymbiont of aphids, *B. aphidicola*, when transcripts from *A. craccivora* were used as queries of which 23 unique transcripts retrieved InterProScan annotations (Table S2) and nine were predicted to be involved in nine different bacterial biochemical pathways (Table 2). Transcripts from six different fungi species were also predicted among *A. craccivora* transcripts, including *Rhizopus delemar* and *Batrachochytrium dendrobatidis* (Figure S1b).

**Table 2:** The orthologs of *A. craccivora* contigs derived from the genome of the primary aphid endosymbiont, *B. aphidicola* (identified in GenBank accession BA000003.2.).

**Contig ID**	**Orthologous *B. aphidicola* gene**	***B. aphidicola* protein (EC)**
Aphis 1561	D-fructose-6-phosphate amidotransferase (glmS)	BAB12753.1 (EC 2.6.1.16)
Aphis 5691	UDP-N-acetylglucosamine pyrophosphorylase (glmU)	BAB12754.1 (EC 2.7.7.23)
Aphis 5225	S-adenosylmethionine synthetase (metK)	BAB13109.1 (EC 2.5.1.6)
Aphis 7020	acetolactate synthase small subunit (ilvH)	BAB12941.1 (EC 2.2.1.6)
Aphis 6159	2-oxoglutarate dehydrogenase e1 component (sucA)	BAB13011.1 (EC 1.2.4.2)
Aphis 5021	ABC transporter ATP-binding protein (uup)	BAB13068.1
Aphis 376	Spermidine synthase (speE)	BAB12926.1 (EC 2.5.1.16)
Aphis 3768	6-phsphoglucanate dehydrogenase (gnd)	BAB12826.1 (EC 1.1.1.44)
Aphis 3870	Hypothetical GTP-binding protein (yfgK)	BAB13291.1

Information regarding protein function can be retrieved from SwissProt database (http://enzyme.expasy.org/) by searches for EC number (not available for all *B. aphidicola* genes)*.*

### Prediction of Putative SNPs

All *A. curvipes*, *A. craccivora*, *C. tomentosicollis* and *M. sjostedti* contigs, that contained a putative ORF, were included in the SNP prediction pipeline (Tables S3a to S3d; Tables S4a to S4d). From these predictions, 256, 30, 225 and 63 contigs respectively from *A. curvipes*, *A. craccivora*, *C. tomentosicollis* and *M. sjostedti* had putative SNPs, which respectively contained a total of 742, 97, 607 and 180 putative SNPs (Tables S3a to S3d; Tables S4a to S4d). The mean depth of reads aligned to the reference transcripts depth for all putative SNPs was > 60 across all species (Table 3). The density of SNPs within transcripts was measured by estimates of mean number of putative SNPs per kilobase, and were all less than 1 (0.09, 0.02, 0.08 and 0.04 respectively among *A. curvipes*, *A. craccivora*, *C. tomentosicollis* and *M. sjostedti* contigs) (Table 3). The alternate ≥ 10% coverage cut off we used comparatively predicted 2,703, 340, 2,087 and 780 putative SNPs for *A. curvipes*, *A. craccivora*, *C. tomentosicollis* and *M. sjostedti*, respectively. As a consequence of 454-based sequence by synthesis methods used, resulting reads are prone to sequencing errors known as homopolymers which comprise imprecise nucleotide numbers in long arrays of the same nucleotide. These errors can cause misalignment within contig assemblies such that incorrect SNP predictions can result in sequence regions flanking the homopolymer stretch. To compensate for these errors, the assembled contigs were filtered for 454/Ion homopolymer INDELS in the SNP detection software in the CLC Genomics Workbench before performing the SNPs prediction. To understand the effects of SNP mutations and associate them to the different transcripts obtained in our study, we differentiated synonymous SNPs from non-synonymous SNPs. Of the total number of SNPs obtained in the four insect species, there were 425, 72, 419 and 97 synonymous SNPs respectively in *A. curvipes*, *A. craccivora*, *C. tomentosicollis* and *M. sjostedti* and respectively 317, 25, 188 and 83 non-synonymous SNPs. In all four species, transitions were more frequent than transversions (Ts/Tv > 1) (Table 3). 

**Table 3:** Summary of the putative single nucleotide polymorphism (SNP) predictions from reads mapped to reference *A. curvipes*, *A. craccivora*, *C. tomentosicollis* and *M. sjostedti* transcripts.

	*A. curvipes*	*A. craccivora*	*C. tomentosicollis*	*M. sjostedti*
Normalization	Normalized	Normalized	Normalized	Normalized
Total number of raw reads	319,126	176,262	320,722	227,882
Mean raw read lengths (bp)	382.7	402.1	389.6	391.5
Total number of processed reads after trimming	304,110	166,565	306,666	211,626
Mean trimmed read length (bp)	315.5	356.5	327.1	340.9
Final processed number of assembled contigs	11,687	7,647	10,652	7,348
Mean length of assembled contig (bp)	688.1	669.8	685.8	683.7
Total number of singletons	219	211	180	115
Mean ORF length (bp)	498.5	514.3	524.5	508.8
Total number of contigs with BLASTx hits	6,430	6,113	6,839	4,292

## Discussion

### Development of Reference Transcriptome Sequence Assemblies

Next generation sequencing technologies offer a rapid entry point into genomic research [44] and can generate valuable molecular resources for non-model species [35,54] that are a foundation from which a diversity of research questions can be addressed [54,55]. In the absence of complete genome sequences, transcriptome sequencing remain a useful molecular resource that can be applied to the identification of candidate insecticide resistance genes and mutations that can be developed into genetic markers for population genetic studies [28], as well as the identification of potential targets for RNAi knockdown. The Roche 454 platform provides long sequence read lengths that may better allow the assembly of *de novo* transcriptomes [54], but remain susceptible to sequencing errors in homopolymer regions. The Roche 454 transcriptome data presented in this study from *A. curvipes*, *A. craccivora*, *C. tomentosicollis* and *M. sjostedti* had a high median length (≥ 680 bp), and a majority of resulting contigs encoded a predicted ORF (= protein coding sequence, CDS). Despite this, a high number of contigs (> 35%) had ORFs with no amino acid similarity to known proteins within the NCBI nr database and was especially the case for *A. curvipes*, *C. tomentosicollis* and *M. sjostedti* transcripts. However, only 20.06% of the *A. craccivora* contigs had no similarity to known proteins in the NCBI nr database. Annotation of previous transcriptome assemblies have similarly revealed a high number of contigs with genes of unknown function [56-59] which may represent novel uncharacterized genes and reflect the limitation of inferring transcript functions by comparison to model species that have long evolutionary distances to the non-model species in question [60]. Even within whole genome sequence assemblies, species-specific genes can comprise a high percentage of predicted ORFs [61]. The presence of these genes of unknown function could similarly suggest these proteins may be species-specific and, that *de novo* transcriptome assemblies from non-model insect pest species are useful for phylogenetic novel gene discovery. Furthermore, the resulting assembly of sequence data allow for the identification of novel gene pathways that have potentials for RNAi targeting within a suite of species-specific control tactics. 

### Functional Annotation

Functional annotations of assembled *A. curvipes*, *A. craccivora*, *C. tomentosicollis* and *M. sjostedti* transcripts allowed for the identification of candidate genes encoding proteins putatively involved in insecticide resistance, and pathogen defense and immunity. Transcriptomic approaches are powerful tools to identify new genes and gene functions and have been successfully applied to many organisms. In this study, we have identified genes putatively involved in the response to and the detoxification of xenobiotics in the four insect pests. Some of these xenobiotic response/detoxification genes will likely be useful for the study of chemical insecticide resistance traits as well the role in detoxification following exposure to plant allelochemicals. For example, strains of *A. craccivora* have elevated esterase activities that were linked to increased resistance to the nicotinic acetylecholine receptor agonist, dinotefuran, which belongs to the third generation of neonicotinoids [62]. Our results will provide a foundation that makes the future study of the involvement of these candidate genes in field-observed insecticide resistance traits in these insects more likely, and may also represent genetic markers that can be used to screen field populations (and compare resistant vs. susceptible individuals) to determine linkage (or not) of the locus to the resistant phenotype trait. 

Our current understanding of insect immunity and stress responses comes from holometabolous insects and includes flies, butterflies, beetles and bees [63]. The four insect pests under study in this paper are all hemimetabolous insects with three of them (*A. curvipes*, *A. craccivora* and *C. tomentosicollis*) falling into the same insect order of Hemiptera and the fourth insect *M. sjostedti* falling into the insect order, Thysanoptera. Because all studied species exhibit incomplete development, comparison with the genome of a hemimetabolous insect (i.e., pea aphid, *A. pisum*) may provide insights into immunity and defense mechanisms in these pest insects. It is also interesting to note that while the four insect species included in this study were not intentionally immunologically challenged, we still observed some transcripts putatively involved in insect defense and immunity based on studies conducted on other insects such as *A. pisum*. We did not observe as many immunity and defense transcripts in both *A. craccivora* and *M. sjostedti* as we observed in *A. curvipes* and *C. tomentosicollis*. The immune genes observed in this study include most genes involved in the IMD pathway in insects and includes chitinase, defensin, down syndrome critical region protein, epidermal growth factor receptor, jnk stimulatory phosphatase, lysozyme, nimrod, nitric oxide synthase, odorant binding protein, peptidoglycan-recognition protein and pro-phenol oxidase. We also observed genes involved in toll signaling pathway. It is interesting to note that none of these genes are represented across all the four insect pests. Some insects have particular genes that others lack, and vice versa. For example, *A. craccivora* appears to be missing the defensin gene, however, a lack of such a gene would have to be verified in the future if a genomic project were to occur for this species. This is consistent with studies conducted on *A. pisum*, which shows the pea aphid is lacking many of the antimicrobial peptides, such as defensin, common to other insects [64]. The reduced humoral immune system in *A. pisum*, including an apparently non-functional IMD signaling pathway and absence of PGRPs, has been suggested to be an adaptation for the symbiosis with the bacterium *B. aphidicola* [65]. The presence of defensin in the human louse, *P. humanus* and in the ancient apterygote insect, the fire brat, *Thermobia domestica* [66], suggests that defensins may have been lost during aphid evolution. 

### Prediction of Putative SNPs

Single nucleotide polymorphisms are rapidly becoming the marker of choice for many applications in population ecology, evolution and conservation genetics, because of the potential for high genotyping efficiency, data quality, genome-wide coverage and analytical simplicity (e.g. in modeling mutational dynamics) [42]. Transcriptome-derived SNPs have several advantages over those developed from genomic sequences [67–69], including acquisition of actual gene sequences that allow for direct mapping and comparative genome studies among organisms ([70] and references therein). SNPs derived from transcriptomes are also a source of candidate polymorphisms underlying important traits that can lead to the identification of quantitative trait nucleotides (QTN) [71] linked to ecologically relevant genes. The applicability of SNPs from sequence data for marker development has been previously reported [72–74] and has been applied for the genetic mapping of insect orders such as Lepidoptera (*Bombyx mori* [75]), and for population genetics of the Glanville fritillary butterfly, *Melitaea cinxia* [76]. The current study provides a set of at least 742, 97, 607 and 180 putative SNPs respectively for *A. curvipes*, *A. craccivora*, *C*. *tomentosicollis* and *M. sjostedti* (predicted using the criteria that SNPs be present in ≥ 35% of aligned reads), and the segregating mutations that can be developed into molecular genetic markers for the study of the population genetic structure of these insect pests. Although a greater number of putative SNPs were predicted using a more lenient coverage cut off value of 10%, these loci may be prone to type I error and secondary validation methods may likely be required to distinguish these from sequencing errors.

The frequency of SNPs in laboratory strains of *Drosophila* was reported at 5 SNP per kilobase [77] and at 1.3 SNPs per kilobase in the inbred Dazao strain of *Bombyx mori* [78]. Similarly, laboratory strains of the malaria mosquito, *Anopheles funestus*, were reported to have 7.2 SNPs per kilobase [79], and 8.0 SNPs per kilobase in *An. gambiae* [80]. Compared to the results obtained from the above studies, we did observe a lower amount of SNPs per kilobase in the present study. Although laboratory strains were used in those studies, we used field-collected insects in our study and usually a reduced SNP frequency is reported in laboratory strains because homozygosity may be increased by the effects of inbreeding or random genetic drift. Non-synonymous SNPs have commonly been reported to occur less frequently than synonymous SNPs, and is presumably due to the evolutionary constraints of negative selection that may eliminate deleterious substitutions from the population [81]. Non-synonymous SNPs are of particular interest because they are more likely to affect the function of the encoded protein and may influence phenotype. It has been estimated that 20–30% of non-synonymous SNPs affect protein function [82,83]. In our study, we did observe a higher number of synonymous SNPs than non-synonymous SNPs across transcripts from all the four insect species. In metazoan DNA sequences, an excess of transition vs. transversion mutations is often observed. This may be partly due to the relatively high rate of change of methylated cytosines to thymine, as well as post-mutation processes of selection on codon-usage bias within coding regions [84]. The role of population genetic and biochemical effects on the rate and direction of nucleotide changes remains unknown, but are likely factors that affect the observed level of SNP allele frequencies within natural populations.

### Metagenomic Identification of Endosymbiont- and Pathogen-Derived Transcripts

Aphids are sap-feeding insects that infest a wide range of plant species. Although sap fluids from plant phloem contain high concentrations of carbohydrates, they are deficient in nitrogenous nutrients such as specific amino acids [85,86]. To overcome these nutritional deficiencies, species within Aphidoidea have established mutualistic relationships with the obligate intracellular endosymbiont, *B. aphidicola* [87,88]. *Buchnera* endosymbionts produce essential amino acids that cannot be synthesized by aphids or obtained in sufficient quantities from plant saps [65,87]. In return, aphids provide *Buchnera* with other nutrients required to survive [89]. Relationships between these two groups have existed for approximately 150 to 200 million years [90] resulting in drastic *Buchnera* genome reductions due to the loss of many genes needed for independent life and has led to the inability to survive outside host cells [91]. Therefore, aphids and associated *Buchnera* symbionts may be inseparable mutualistic partners. We observed 36 *B. aphidicola* transcripts among BLASTn hits to our *A. craccivora* transcripts. These 36 *B. aphidicola* transcripts were annotated from twelve different strains of *B. aphidicola*. We also observed ubiquinone in the BLASTx search of *A. craccivora*. Symbiotic *B. aphidicola* are aerobic bacterium which, due to gene reduction in metabolic pathways, cannot carry out respiration without obtaining gene products from the host [92]. The electron transport chain consists of a primary dehydrogenase and a terminal reductase, which are linked by ubiquinone [93]. Ubiquinone is an essential redox component of the aerobic respiration of bacteria and mitochondria [94], and participates in the transfer of electrons and hydrogen between flavoproteins and cytochrome b in the respiratory chain. Also, one of the 36 *A. craccivora* contigs with hits to *B. aphidicola* was annotated as the gene symbionin, which has been reported to increase the transmission of plant viruses by binding to the read-through domain of the viral coat protein [95]. Further study of these genes may likely lead to a better understanding of symbiosis and plant disease transmission by *A. craccivora*, and may lead to potential tactics to reduce or eliminate the disease vectoring capacity of *A. craccivora*.

Additionally, we observed BLASTx hits to six fungi species among *A. craccivora* sequences. *Rhizopus oryzae* (*R. oryzae* has been reclassified to include *R. oryzae* and *R. delemar* [96]. *Rhizopus delemar* was observed in the BLASTx hits in this study) was previously reported to be an entomopathogenic fungal species [97]. *Batrachochytrium dendrobatidis* causes chytridiomycosis and is a major cause of amphibian population decline worldwide [98] and the sequences within our aphid transcripts may be derived from a related fungal species that is capable of infecting *A. craccivora* in West Africa. In contrast, *Melampsora larici-populina* is a cause of rust in poplar trees [99]. These may have resulted from environmental contamination or were present within gut contents of whole aphids that were used for library preparations. These results suggest that application of NGS may be used for the metagenomic identification of putative pathogen species, which in turn may be useful for the biological control of pest insect species.

## Conclusion

With the exception of prior studies that focused on *M. vitrata*, this study represents the first attempt to develop transcriptomic and molecular marker data for field insect pests of cowpea in West Africa.  Although the sequence data, and biological functions of these genes, may be of interest and importance to molecular biologists, the molecular markers are potentially of much greater near-term pragmatic importance for those interested in controlling these pests. Previous studies have already demonstrated that such molecular markers can give us important insights into pest movement patterns that ultimately will impact how pest control strategies for *M. vitrata* need to be developed in different agro-ecological zones in West Africa [28,29]. For example, *M. vitrata* is an endemic pest in the southern part of the selected West African countries and migratory in the northern part; thus biocontrol agents need to be released in the south and spraying of pesticides or biopesticides [22] may be a better solution in areas where these insects are not endemic. This understanding has emerged from a combination of studies on the biology of this pest and through the use of molecular markers. We term this approach, combining traditional IPM strategies with knowledge that emerges from population genetics/genomics tools, IPM-omics [100].  This study lays the foundation for research in other pest species, with the long-term goal to develop a comprehensive program that integrates genomics datasets into IPM and IRM programs in order to minimize the crop damage inflicted by pest insect species of cowpea in West Africa.

## Supporting Information

Figure S1 **Species distribution of the top BLASTx hits in (a) *A. curvipes*, (b) *A. craccivora*, (c) *C. tomentosicollis*, (d) *M. sjostedti*.**
(TIF)

Table S1 a. Counts of all the genes identified in the gene ontology analysis of all the contigs present in *A. curvipes*. b. Counts of all the genes identified in the gene ontology analysis of all the contigs present in *A. craccivora*. c. Counts of all the genes identified in the gene ontology analysis of all the contigs present in *C. tomentosicollis*. d. Counts of all the genes identified in the gene ontology analysis of all the contigs present in *M. sjostedti*.
(DOCX)

Table S2 **InterproScan results from *A. craccivora* contigs that showed BLASTn hits to *B. aphidicola***.
(DOCX)

Table S3 a. Summary of all SNPs detected in contigs in *A. curvipes*, including sequence description, length, organism, minimum e-value, number of GOs and number of SNPs associated with each contig. b. Summary of all SNPs detected in contigs in *A. craccivora*, including sequence description, length, organism, minimum e-value, number of GOs and number of SNPs associated with each contig. c. Summary of all SNPs detected in contigs in *C. tomentosicollis*, including sequence description, length, organism, minimum e-value, number of GOs and number of SNPs associated with each contig. d. Summary of all SNPs detected in contigs in *M. sjostedti*, including sequence description, length, organism, minimum e-value, number of GOs and number of SNPs associated with each contig.
(DOCX)

Table S4 a. List of all SNPs detected in *A. curvipes*, including consensus positions on contigs, alleles, coverage, frequency and contig sequences. b. List of all SNPs detected in *A. craccivora*, including consensus positions on contigs, alleles, coverage, frequency and contig sequences. c. List of all SNPs detected in *C. tomentosicollis*, including consensus positions on contigs, alleles, coverage, frequency and contig sequences. d. List of all SNPs detected in M. sjostedti, including consensus positions on contigs, alleles, coverage, frequency and contig sequences.
(XLSX)
