# Supplementary figures and images for: Development of Reference Transcriptomes for the Major Field Insect Pests of Cowpea: A Toolbox for Insect Pest Management Approaches in West Africa
Source: PLoS One. 2013 Nov 22;8(11):e79929. doi: 10.1371/journal.pone.0079929 (PMC3838393; doi:10.1371/journal.pone.0079929)

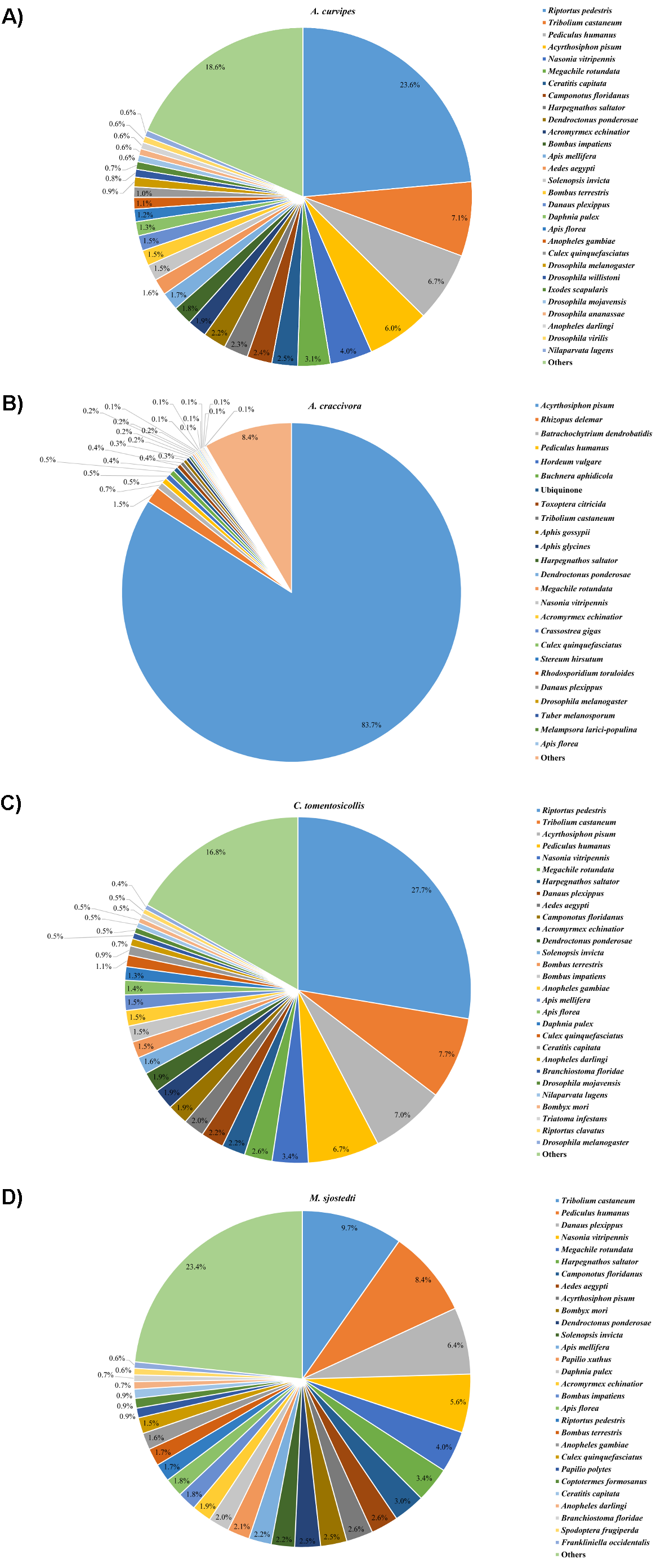

Supplement: Figure S1 — Species distribution of the top BLASTx hits in (a) A. curvipes, (b) A. craccivora, (c) C. tomentosicollis, (d) M. sjostedti. (TIF) [file pone.0079929.s001.tif]
