# Supplementary material for: Development of Reference Transcriptomes for the Major Field Insect Pests of Cowpea: A Toolbox for Insect Pest Management Approaches in West Africa
Source: PLoS One. 2013 Nov 22;8(11):e79929. doi: 10.1371/journal.pone.0079929 (PMC3838393; doi:10.1371/journal.pone.0079929)
Supplement: Table S1 — a. Counts of all the genes identified in the gene ontology analysis of all the contigs present in A. curvipes. b. Counts of all the genes identified in the gene ontology analysis of all the contigs present in A. craccivora. c. Counts of all the genes identified in the gene ontology analysis of all the contigs present in C. tomentosicollis. d. Counts of all the genes identified in the gene ontology analysis of all the contigs present in M. sjostedti. (DOCX) [file pone.0079929.s002.docx]

**Table S1a.**

| **GO Level** | **Term (Name)** | **#Sequence** | **Parents (Name)** | **Category** |
| --- | --- | --- | --- | --- |
| 2 | metabolic process | 2166 | biological_process | Biological Process |
| null | single-organism process | 1313 | biological_process | Biological Process |
| null | single-organism cellular process | 1088 | single-organism process, cellular process | Biological Process |
| 3 | primary metabolic process | 1616 | metabolic process | Biological Process |
| 2 | cellular process | 1880 | biological_process | Biological Process |
| 3 | regulation of biological process | 870 | biological regulation, biological_process | Biological Process |
| 3 | multicellular organismal development | 684 | single-multicellular organism process, developmental process | Biological Process |
| 2 | developmental process | 748 | biological_process | Biological Process |
| 3 | cellular component organization | 755 | cellular component organization or biogenesis | Biological Process |
| 3 | organic substance metabolic process | 1588 | metabolic process | Biological Process |
| 4 | protein metabolic process | 962 | primary metabolic process, macromolecule metabolic process | Biological Process |
| 2 | response to stimulus | 684 | biological_process | Biological Process |
| 4 | transport | 638 | establishment of localization | Biological Process |
| 3 | catabolic process | 519 | metabolic process | Biological Process |
| 4 | nucleobase-containing compound metabolic process | 564 | heterocycle metabolic process, primary metabolic process, organic cyclic compound metabolic process, cellular aromatic compound metabolic process, cellular nitrogen compound metabolic process | Biological Process |
| 2 | biological regulation | 914 | biological_process | Biological Process |
| null | single-multicellular organism process | 685 | multicellular organismal process, single-organism process | Biological Process |
| 3 | cellular metabolic process | 1270 | metabolic process, cellular process | Biological Process |
| 3 | biosynthetic process | 713 | metabolic process | Biological Process |
| 4 | cell differentiation | 427 | cellular developmental process | Biological Process |
| 5 | cellular protein metabolic process | 693 | protein metabolic process, cellular macromolecule metabolic process | Biological Process |
| 2 | cellular component organization or biogenesis | 755 | biological_process | Biological Process |
| 5 | organelle organization | 473 | single-organism cellular process, cellular component organization | Biological Process |
| 3 | macromolecule metabolic process | 1050 | organic substance metabolic process | Biological Process |
| 6 | translation | 376 | cellular macromolecule biosynthetic process, cellular protein metabolic process, gene expression | Biological Process |
| 4 | anatomical structure morphogenesis | 359 | anatomical structure development, developmental process | Biological Process |
| 4 | signal transduction | 336 | single organism signaling, cell communication, cellular response to stimulus, regulation of cellular process | Biological Process |
| 3 | response to stress | 334 | response to stimulus | Biological Process |
| 6 | cellular protein modification process | 331 | cellular protein metabolic process, protein modification process | Biological Process |
| 3 | establishment of localization | 638 | biological_process, localization | Biological Process |
| 4 | cellular macromolecule metabolic process | 770 | cellular metabolic process, macromolecule metabolic process | Biological Process |
| 4 | heterocycle metabolic process | 564 | cellular metabolic process | Biological Process |
| 4 | cellular nitrogen compound metabolic process | 564 | cellular metabolic process, nitrogen compound metabolic process | Biological Process |
| null | organic cyclic compound metabolic process | 564 | organic substance metabolic process | Biological Process |
| 4 | cellular aromatic compound metabolic process | 564 | cellular metabolic process | Biological Process |
| 3 | anatomical structure development | 401 | developmental process | Biological Process |
| 3 | cell cycle | 299 | single-organism cellular process | Biological Process |
| 3 | cell communication | 422 | single-organism cellular process | Biological Process |
| 2 | multicellular organismal process | 685 | biological_process | Biological Process |
| null | single organism signaling | 409 | single-organism process, signaling | Biological Process |
| 3 | cellular developmental process | 427 | developmental process, single-organism cellular process | Biological Process |
| 6 | cytoskeleton organization | 256 | organelle organization | Biological Process |
| 2 | reproduction | 243 | biological_process | Biological Process |
| 4 | gene expression | 399 | macromolecule metabolic process | Biological Process |
| 5 | cellular macromolecule biosynthetic process | 376 | cellular macromolecule metabolic process, cellular biosynthetic process, macromolecule biosynthetic process | Biological Process |
| 4 | regulation of cellular process | 336 | cellular process, regulation of biological process | Biological Process |
| 3 | cellular response to stimulus | 336 | response to stimulus, single-organism cellular process | Biological Process |
| 5 | protein modification process | 331 | macromolecule modification, protein metabolic process | Biological Process |
| 2 | localization | 639 | biological_process | Biological Process |
| 3 | nitrogen compound metabolic process | 564 | metabolic process | Biological Process |
| 4 | carbohydrate metabolic process | 185 | primary metabolic process, organic substance metabolic process | Biological Process |
| 4 | generation of precursor metabolites and energy | 184 | cellular metabolic process | Biological Process |
| 6 | protein transport | 184 | organic substance transport, establishment of protein localization | Biological Process |
| 2 | signaling | 409 | biological_process | Biological Process |
| 5 | ion transport | 145 | single-organism transport | Biological Process |
| 4 | embryo development | 140 | single-organism developmental process, multicellular organismal development, anatomical structure development | Biological Process |
| 3 | cell death | 136 | death, single-organism cellular process | Biological Process |
| 4 | cellular biosynthetic process | 376 | cellular metabolic process, biosynthetic process | Biological Process |
| 4 | macromolecule biosynthetic process | 376 | organic substance biosynthetic process, macromolecule metabolic process | Biological Process |
| 4 | macromolecule modification | 331 | macromolecule metabolic process | Biological Process |
| 4 | lipid metabolic process | 115 | primary metabolic process, organic substance metabolic process, single-organism metabolic process | Biological Process |
| 5 | organic substance transport | 184 | transport | Biological Process |
| 5 | establishment of protein localization | 184 | establishment of localization, protein localization | Biological Process |
| 6 | DNA metabolic process | 108 | nucleic acid metabolic process, cellular macromolecule metabolic process | Biological Process |
| 3 | cell-cell signaling | 105 | single organism signaling, cell communication | Biological Process |
| 3 | behavior | 105 | response to stimulus | Biological Process |
| 3 | response to external stimulus | 103 | response to stimulus | Biological Process |
| 3 | cellular homeostasis | 95 | homeostatic process, single-organism cellular process | Biological Process |
| 2 | growth | 97 | biological_process | Biological Process |
| null | single-organism metabolic process | 148 | metabolic process | Biological Process |
| 2 | cell proliferation | 89 | single-organism process | Biological Process |
| null | single-organism transport | 145 | single-organism process, transport | Biological Process |
| null | single-organism developmental process | 140 | single-organism process, developmental process | Biological Process |
| 2 | death | 136 | single-organism process | Biological Process |
| null | organic substance biosynthetic process | 376 | organic substance metabolic process, biosynthetic process | Biological Process |
| 3 | response to abiotic stimulus | 79 | response to stimulus | Biological Process |
| 3 | response to biotic stimulus | 77 | response to stimulus | Biological Process |
| 4 | protein localization | 185 | macromolecule localization | Biological Process |
| 5 | nucleic acid metabolic process | 108 | macromolecule metabolic process, nucleobase-containing compound metabolic process | Biological Process |
| 4 | homeostatic process | 95 | regulation of biological quality | Biological Process |
| 6 | mitochondrion organization | 53 | organelle organization | Biological Process |
| 3 | response to endogenous stimulus | 43 | response to stimulus | Biological Process |
| 3 | macromolecule localization | 185 | localization | Biological Process |
| 3 | secondary metabolic process | 38 | single-organism metabolic process | Biological Process |
| 3 | regulation of biological quality | 95 | biological regulation | Biological Process |
| 2 | viral reproduction | 29 | multi-organism cellular process | Biological Process |
| 3 | cell recognition | 25 | single-multicellular organism process, single-organism cellular process | Biological Process |
| 7 | regulation of gene expression, epigenetic | 25 | regulation of gene expression | Biological Process |
| 5 | cytoplasm organization | 22 | single-organism cellular process, cellular component organization | Biological Process |
| 3 | cell growth | 19 | growth, single-organism cellular process | Biological Process |
| null | multi-organism cellular process | 29 | multi-organism process, cellular process | Biological Process |
| 2 | multi-organism process | 32 | biological_process | Biological Process |
| 4 | symbiosis, encompassing mutualism through parasitism | 16 | interspecies interaction between organisms | Biological Process |
| 6 | regulation of gene expression | 25 | gene expression, regulation of macromolecule metabolic process | Biological Process |
| 3 | interspecies interaction between organisms | 16 | multi-organism process | Biological Process |
| 5 | regulation of macromolecule metabolic process | 25 | macromolecule metabolic process, regulation of metabolic process | Biological Process |
| 4 | regulation of metabolic process | 25 | regulation of biological process, metabolic process | Biological Process |
| 7 | glycine receptor clustering | 1 | postsynaptic membrane organization, neurotransmitter-gated ion channel clustering | Biological Process |
| 8 | neurotransmitter-gated ion channel clustering | 1 | receptor clustering, synapse assembly | Biological Process |
| 4 | postsynaptic membrane organization | 1 | cellular membrane organization | Biological Process |
| 6 | synapse assembly | 1 | nervous system development, synapse organization, cellular component assembly | Biological Process |
| 3 | cellular membrane organization | 1 | single-organism cellular process, membrane organization | Biological Process |
| 7 | receptor clustering | 1 | protein localization to membrane | Biological Process |
| 4 | protein localization to membrane | 1 | cellular membrane organization, cellular protein localization | Biological Process |
| 4 | cellular component assembly | 1 | cellular component biogenesis, cellular component organization | Biological Process |
| 4 | membrane organization | 1 | cellular component organization | Biological Process |
| 5 | nervous system development | 1 | system development | Biological Process |
| 4 | synapse organization | 1 | single-organism cellular process, cellular component organization | Biological Process |
| 4 | system development | 1 | multicellular organismal development, anatomical structure development | Biological Process |
| 5 | cellular protein localization | 1 | cellular macromolecule localization, protein localization | Biological Process |
| 3 | cellular component biogenesis | 1 | cellular component organization or biogenesis | Biological Process |
| 4 | cellular macromolecule localization | 1 | macromolecule localization, cellular localization | Biological Process |
| 3 | cellular localization | 1 | localization, single-organism cellular process | Biological Process |
| 2 | cell | 2494 | cellular_component | Cellular Component |
| 4 | intracellular part | 1949 | cell part, intracellular | Cellular Component |
| 5 | cytoplasm | 1502 | intracellular part | Cellular Component |
| 4 | intracellular | 2151 | cell part | Cellular Component |
| 3 | cell part | 2208 | cell, cellular_component | Cellular Component |
| 5 | cytoplasmic part | 1071 | intracellular part, cytoplasm | Cellular Component |
| 6 | intracellular membrane-bounded organelle | 1287 | intracellular organelle, membrane-bounded organelle | Cellular Component |
| 5 | intracellular organelle | 1639 | organelle, intracellular part | Cellular Component |
| 3 | protein complex | 733 | macromolecular complex | Cellular Component |
| 7 | nucleus | 656 | intracellular membrane-bounded organelle | Cellular Component |
| 2 | organelle | 1692 | cellular_component | Cellular Component |
| 2 | macromolecular complex | 966 | cellular_component | Cellular Component |
| 3 | membrane-bounded organelle | 1287 | organelle | Cellular Component |
| 6 | intracellular non-membrane-bounded organelle | 687 | intracellular organelle, non-membrane-bounded organelle | Cellular Component |
| 6 | mitochondrion | 424 | cytoplasmic part, intracellular membrane-bounded organelle | Cellular Component |
| 7 | cytoskeleton | 312 | intracellular non-membrane-bounded organelle | Cellular Component |
| 6 | cytosol | 273 | cytoplasmic part | Cellular Component |
| 3 | non-membrane-bounded organelle | 687 | organelle | Cellular Component |
| 6 | ribosome | 245 | ribonucleoprotein complex, cytoplasmic part, intracellular non-membrane-bounded organelle | Cellular Component |
| 6 | lipid particle | 199 | cytoplasmic part | Cellular Component |
| 6 | nuclear part | 278 | intracellular organelle part, nucleus | Cellular Component |
| 4 | plasma membrane | 187 | cell part, cell periphery, membrane | Cellular Component |
| 7 | nuclear lumen | 236 | nuclear part, intracellular organelle lumen | Cellular Component |
| 6 | nucleoplasm | 147 | nuclear part, nuclear lumen | Cellular Component |
| 5 | ribonucleoprotein complex | 245 | macromolecular complex, intracellular part | Cellular Component |
| 4 | intracellular organelle part | 321 | organelle part, intracellular organelle, intracellular part | Cellular Component |
| 6 | endoplasmic reticulum | 132 | cytoplasmic part, intracellular membrane-bounded organelle | Cellular Component |
| 2 | extracellular region | 186 | cellular_component | Cellular Component |
| 4 | cell periphery | 188 | cell part | Cellular Component |
| 2 | membrane | 187 | cellular_component | Cellular Component |
| 6 | nucleolus | 106 | nuclear part, nuclear lumen, intracellular non-membrane-bounded organelle | Cellular Component |
| 6 | Golgi apparatus | 104 | cytoplasmic part, intracellular membrane-bounded organelle | Cellular Component |
| 6 | intracellular organelle lumen | 236 | intracellular organelle part, organelle lumen | Cellular Component |
| 7 | chromosome | 95 | intracellular non-membrane-bounded organelle | Cellular Component |
| 3 | organelle part | 321 | cellular_component, organelle | Cellular Component |
| 4 | extracellular space | 78 | extracellular region part | Cellular Component |
| 7 | cytoplasmic membrane-bounded vesicle | 67 | cytoplasmic vesicle, intracellular membrane-bounded organelle, membrane-bounded vesicle | Cellular Component |
| 4 | organelle lumen | 236 | organelle part, membrane-enclosed lumen | Cellular Component |
| 7 | microtubule organizing center | 57 | microtubule cytoskeleton, cytoskeletal part | Cellular Component |
| 3 | extracellular region part | 90 | cellular_component, extracellular region | Cellular Component |
| 5 | nuclear envelope | 48 | nuclear part, organelle envelope, endomembrane system | Cellular Component |
| 4 | membrane-bounded vesicle | 67 | vesicle | Cellular Component |
| 6 | cytoplasmic vesicle | 67 | cytoplasmic part, vesicle, intracellular organelle | Cellular Component |
| 2 | membrane-enclosed lumen | 236 | cellular_component | Cellular Component |
| 6 | cytoskeletal part | 57 | intracellular organelle part, cytoskeleton | Cellular Component |
| 8 | microtubule cytoskeleton | 57 | cytoskeleton | Cellular Component |
| 6 | nuclear chromosome | 30 | nuclear part, nuclear lumen, chromosome | Cellular Component |
| 4 | organelle envelope | 48 | intracellular organelle part, membrane-bounded organelle, envelope | Cellular Component |
| 4 | endomembrane system | 48 | cell part | Cellular Component |
| 6 | vacuole | 36 | cytoplasmic part, intracellular membrane-bounded organelle | Cellular Component |
| 3 | vesicle | 67 | organelle | Cellular Component |
| 6 | endosome | 24 | cytoplasmic part, intracellular membrane-bounded organelle | Cellular Component |
| 7 | peroxisome | 21 | microbody | Cellular Component |
| 4 | envelope | 49 | cell part | Cellular Component |
| 4 | proteinaceous extracellular matrix | 15 | extracellular region part, extracellular matrix | Cellular Component |
| 8 | lysosome | 13 | lytic vacuole | Cellular Component |
| 6 | microbody | 21 | cytoplasmic part, intracellular membrane-bounded organelle | Cellular Component |
| 2 | extracellular matrix | 15 | cellular_component | Cellular Component |
| 5 | cilium | 8 | cell projection, intracellular membrane-bounded organelle | Cellular Component |
| 7 | lytic vacuole | 13 | vacuole | Cellular Component |
| 4 | cell projection | 8 | cell part | Cellular Component |
| 6 | plastid | 4 | cytoplasmic part, intracellular membrane-bounded organelle | Cellular Component |
| 4 | external encapsulating structure | 3 | cell part, cell periphery | Cellular Component |
| 5 | cell envelope | 1 | envelope | Cellular Component |
| 5 | cell wall | 1 | external encapsulating structure | Cellular Component |
| 5 | thylakoid | 1 | intracellular part | Cellular Component |
| 2 | binding | 2108 | molecular_function | Molecular Function |
| 2 | catalytic activity | 2218 | molecular_function | Molecular Function |
| 4 | nucleotide binding | 806 | small molecule binding, nucleoside phosphate binding | Molecular Function |
| 3 | hydrolase activity | 925 | catalytic activity | Molecular Function |
| 3 | protein binding | 668 | binding | Molecular Function |
| 3 | organic cyclic compound binding | 1169 | binding | Molecular Function |
| null | heterocyclic compound binding | 1164 | binding | Molecular Function |
| 3 | small molecule binding | 806 | binding | Molecular Function |
| null | nucleoside phosphate binding | 806 | heterocyclic compound binding, organic cyclic compound binding | Molecular Function |
| 3 | transferase activity | 571 | catalytic activity | Molecular Function |
| 3 | nucleic acid binding | 548 | heterocyclic compound binding, organic cyclic compound binding | Molecular Function |
| 2 | transporter activity | 343 | molecular_function | Molecular Function |
| 2 | structural molecule activity | 304 | molecular_function | Molecular Function |
| 4 | peptidase activity | 226 | hydrolase activity | Molecular Function |
| 4 | RNA binding | 251 | nucleic acid binding | Molecular Function |
| 4 | DNA binding | 179 | nucleic acid binding | Molecular Function |
| 5 | kinase activity | 186 | transferase activity, transferring phosphorus-containing groups | Molecular Function |
| 6 | protein kinase activity | 101 | kinase activity, phosphotransferase activity, alcohol group as acceptor | Molecular Function |
| 2 | enzyme regulator activity | 95 | molecular_function | Molecular Function |
| 4 | transferase activity, transferring phosphorus-containing groups | 186 | transferase activity | Molecular Function |
| 4 | cytoskeletal protein binding | 94 | protein binding | Molecular Function |
| 5 | translation factor activity, nucleic acid binding | 82 | RNA binding | Molecular Function |
| 6 | calcium ion binding | 72 | metal ion binding | Molecular Function |
| 2 | electron carrier activity | 63 | molecular_function | Molecular Function |
| 5 | actin binding | 62 | cytoskeletal protein binding | Molecular Function |
| 5 | phosphotransferase activity, alcohol group as acceptor | 101 | transferase activity, transferring phosphorus-containing groups | Molecular Function |
| 3 | lipid binding | 53 | binding | Molecular Function |
| 4 | receptor binding | 47 | protein binding | Molecular Function |
| 2 | receptor activity | 45 | molecular_function | Molecular Function |
| 4 | hydrolase activity, acting on ester bonds | 99 | hydrolase activity | Molecular Function |
| 5 | metal ion binding | 72 | cation binding | Molecular Function |
| 5 | nuclease activity | 41 | hydrolase activity, acting on ester bonds | Molecular Function |
| 3 | sequence-specific DNA binding transcription factor activity | 40 | nucleic acid binding transcription factor activity | Molecular Function |
| 7 | phosphoprotein phosphatase activity | 40 | phosphatase activity | Molecular Function |
| 3 | carbohydrate binding | 39 | binding | Molecular Function |
| null | transcription regulator activity | 37 | obsolete_molecular_function | Molecular Function |
| 6 | ion channel activity | 28 | substrate-specific channel activity, ion transmembrane transporter activity | Molecular Function |
| 3 | signal transducer activity | 27 | molecular transducer activity | Molecular Function |
| 4 | cation binding | 72 | ion binding | Molecular Function |
| 8 | motor activity | 25 | nucleoside-triphosphatase activity | Molecular Function |
| 6 | phosphatase activity | 41 | phosphoric ester hydrolase activity | Molecular Function |
| 2 | nucleic acid binding transcription factor activity | 40 | molecular_function | Molecular Function |
| null | obsolete_molecular_function | 37 |  | Molecular Function |
| 2 | nutrient reservoir activity | 21 | molecular_function | Molecular Function |
| 3 | chromatin binding | 21 | binding | Molecular Function |
| 5 | carboxylic ester hydrolase activity | 17 | hydrolase activity, acting on ester bonds | Molecular Function |
| 5 | ion transmembrane transporter activity | 28 | substrate-specific transmembrane transporter activity | Molecular Function |
| 5 | substrate-specific channel activity | 28 | substrate-specific transmembrane transporter activity, channel activity | Molecular Function |
| 2 | molecular transducer activity | 27 | molecular_function | Molecular Function |
| 3 | ion binding | 72 | binding | Molecular Function |
| 7 | nucleoside-triphosphatase activity | 25 | pyrophosphatase activity | Molecular Function |
| 5 | phosphoric ester hydrolase activity | 41 | hydrolase activity, acting on ester bonds | Molecular Function |
| 2 | antioxidant activity | 13 | molecular_function | Molecular Function |
| 4 | substrate-specific transmembrane transporter activity | 28 | substrate-specific transporter activity, transmembrane transporter activity | Molecular Function |
| 5 | channel activity | 28 | passive transmembrane transporter activity | Molecular Function |
| 6 | pyrophosphatase activity | 25 | hydrolase activity, acting on acid anhydrides, in phosphorus-containing anhydrides | Molecular Function |
| 3 | transmembrane transporter activity | 28 | transporter activity | Molecular Function |
| 3 | substrate-specific transporter activity | 28 | transporter activity | Molecular Function |
| 4 | passive transmembrane transporter activity | 28 | transmembrane transporter activity | Molecular Function |
| 2 | translation regulator activity | 6 | molecular_function | Molecular Function |
| 5 | hydrolase activity, acting on acid anhydrides, in phosphorus-containing anhydrides | 25 | hydrolase activity, acting on acid anhydrides | Molecular Function |
| 4 | hydrolase activity, acting on acid anhydrides | 25 | hydrolase activity | Molecular Function |
| 8 | inositol monophosphate phosphatase activity | 1 | inositol phosphate phosphatase activity | Molecular Function |
| 3 | oxygen binding | 1 | binding | Molecular Function |
| 7 | inositol phosphate phosphatase activity | 1 | phosphatase activity | Molecular Function |

**Table S1b.**

| **GO Level** | **Term (Name)** | **#Sequence** | **Parents (Name)** | **Category** |
| --- | --- | --- | --- | --- |
| 3 | regulation of biological process | 946 | biological regulation, biological_process | Biological Process |
| 2 | metabolic process | 2170 | biological_process | Biological Process |
| 3 | catabolic process | 565 | metabolic process | Biological Process |
| 3 | multicellular organismal development | 566 | single-multicellular organism process, developmental process | Biological Process |
| 4 | nucleobase-containing compound metabolic process | 614 | heterocycle metabolic process, primary metabolic process, organic cyclic compound metabolic process, cellular aromatic compound metabolic process, cellular nitrogen compound metabolic process | Biological Process |
| 3 | biosynthetic process | 806 | metabolic process | Biological Process |
| 4 | transport | 651 | establishment of localization | Biological Process |
| 4 | signal transduction | 408 | single organism signaling, cell communication, cellular response to stimulus, regulation of cellular process | Biological Process |
| 4 | cell differentiation | 401 | cellular developmental process | Biological Process |
| 3 | cellular component organization | 654 | cellular component organization or biogenesis | Biological Process |
| 6 | cellular protein modification process | 358 | cellular protein metabolic process, protein modification process | Biological Process |
| 6 | translation | 328 | cellular macromolecule biosynthetic process, cellular protein metabolic process, gene expression | Biological Process |
| 4 | protein metabolic process | 906 | primary metabolic process, macromolecule metabolic process | Biological Process |
| 4 | anatomical structure morphogenesis | 316 | anatomical structure development, developmental process | Biological Process |
| 3 | response to stress | 287 | response to stimulus | Biological Process |
| 5 | organelle organization | 413 | single-organism cellular process, cellular component organization | Biological Process |
| 3 | cell cycle | 245 | single-organism cellular process | Biological Process |
| 2 | reproduction | 219 | biological_process | Biological Process |
| 4 | carbohydrate metabolic process | 203 | primary metabolic process, organic substance metabolic process | Biological Process |
| 4 | generation of precursor metabolites and energy | 199 | cellular metabolic process | Biological Process |
| 6 | cytoskeleton organization | 186 | organelle organization | Biological Process |
| 5 | ion transport | 185 | single-organism transport | Biological Process |
| 6 | protein transport | 185 | organic substance transport, establishment of protein localization | Biological Process |
| 4 | lipid metabolic process | 173 | primary metabolic process, organic substance metabolic process, single-organism metabolic process | Biological Process |
| 4 | embryo development | 136 | single-organism developmental process, multicellular organismal development, anatomical structure development | Biological Process |
| 3 | cell-cell signaling | 128 | single organism signaling, cell communication | Biological Process |
| 6 | DNA metabolic process | 117 | nucleic acid metabolic process, cellular macromolecule metabolic process | Biological Process |
| 3 | cell death | 112 | death, single-organism cellular process | Biological Process |
| 3 | behavior | 111 | response to stimulus | Biological Process |
| 3 | response to external stimulus | 96 | response to stimulus | Biological Process |
| 2 | cell proliferation | 93 | single-organism process | Biological Process |
| 2 | growth | 100 | biological_process | Biological Process |
| 3 | cellular homeostasis | 71 | homeostatic process, single-organism cellular process | Biological Process |
| 3 | response to abiotic stimulus | 68 | response to stimulus | Biological Process |
| 3 | secondary metabolic process | 50 | single-organism metabolic process | Biological Process |
| 3 | response to endogenous stimulus | 45 | response to stimulus | Biological Process |
| 2 | viral reproduction | 41 | multi-organism cellular process | Biological Process |
| 6 | mitochondrion organization | 39 | organelle organization | Biological Process |
| 3 | response to biotic stimulus | 35 | response to stimulus | Biological Process |
| 3 | cell communication | 495 | single-organism cellular process | Biological Process |
| 3 | cell recognition | 24 | single-multicellular organism process, single-organism cellular process | Biological Process |
| 7 | regulation of gene expression, epigenetic | 24 | regulation of gene expression | Biological Process |
| 3 | primary metabolic process | 1652 | metabolic process | Biological Process |
| 4 | symbiosis, encompassing mutualism through parasitism | 21 | interspecies interaction between organisms | Biological Process |
| 3 | cell growth | 19 | growth, single-organism cellular process | Biological Process |
| 5 | cytoplasm organization | 17 | single-organism cellular process, cellular component organization | Biological Process |
| 5 | intracellular signal transduction | 1 | signal transduction | Biological Process |
| 5 | cellular response to drug | 1 | cellular response to chemical stimulus, response to drug | Biological Process |
| 2 | cellular process | 1881 | biological_process | Biological Process |
| 4 | cellular response to chemical stimulus | 1 | response to chemical stimulus, cellular response to stimulus | Biological Process |
| 3 | regulation of biological quality | 71 | biological regulation | Biological Process |
| 2 | localization | 651 | biological_process | Biological Process |
| 2 | response to stimulus | 662 | biological_process | Biological Process |
| 3 | organic substance metabolic process | 1638 | metabolic process | Biological Process |
| 3 | cellular developmental process | 401 | developmental process, single-organism cellular process | Biological Process |
| 4 | cellular biosynthetic process | 328 | cellular metabolic process, biosynthetic process | Biological Process |
| null | organic substance biosynthetic process | 328 | organic substance metabolic process, biosynthetic process | Biological Process |
| null | single-organism cellular process | 1023 | single-organism process, cellular process | Biological Process |
| 5 | cellular protein metabolic process | 671 | protein metabolic process, cellular macromolecule metabolic process | Biological Process |
| null | single-organism metabolic process | 210 | metabolic process | Biological Process |
| 3 | anatomical structure development | 352 | developmental process | Biological Process |
| null | multi-organism cellular process | 41 | multi-organism process, cellular process | Biological Process |
| 5 | cellular macromolecule biosynthetic process | 328 | cellular macromolecule metabolic process, cellular biosynthetic process, macromolecule biosynthetic process | Biological Process |
| 4 | cellular macromolecule metabolic process | 760 | cellular metabolic process, macromolecule metabolic process | Biological Process |
| 4 | regulation of metabolic process | 24 | regulation of biological process, metabolic process | Biological Process |
| 2 | cellular component organization or biogenesis | 654 | biological_process | Biological Process |
| 2 | multi-organism process | 46 | biological_process | Biological Process |
| 6 | regulation of gene expression | 24 | gene expression, regulation of macromolecule metabolic process | Biological Process |
| 4 | heterocycle metabolic process | 614 | cellular metabolic process | Biological Process |
| 5 | organic substance transport | 185 | transport | Biological Process |
| 5 | establishment of protein localization | 185 | establishment of localization, protein localization | Biological Process |
| 2 | death | 112 | single-organism process | Biological Process |
| null | single organism signaling | 480 | single-organism process, signaling | Biological Process |
| 5 | nucleic acid metabolic process | 117 | macromolecule metabolic process, nucleobase-containing compound metabolic process | Biological Process |
| 5 | regulation of macromolecule metabolic process | 24 | macromolecule metabolic process, regulation of metabolic process | Biological Process |
| null | single-organism process | 1256 | biological_process | Biological Process |
| 3 | macromolecule metabolic process | 1002 | organic substance metabolic process | Biological Process |
| 4 | homeostatic process | 71 | regulation of biological quality | Biological Process |
| 4 | cellular nitrogen compound metabolic process | 614 | cellular metabolic process, nitrogen compound metabolic process | Biological Process |
| 2 | signaling | 480 | biological_process | Biological Process |
| null | single-organism transport | 185 | single-organism process, transport | Biological Process |
| null | single-organism developmental process | 136 | single-organism process, developmental process | Biological Process |
| 3 | response to chemical stimulus | 1 | response to stimulus | Biological Process |
| null | organic cyclic compound metabolic process | 614 | organic substance metabolic process | Biological Process |
| 2 | multicellular organismal process | 566 | biological_process | Biological Process |
| 4 | macromolecule biosynthetic process | 328 | organic substance biosynthetic process, macromolecule metabolic process | Biological Process |
| 2 | biological regulation | 970 | biological_process | Biological Process |
| 3 | macromolecule localization | 185 | localization | Biological Process |
| 3 | nitrogen compound metabolic process | 614 | metabolic process | Biological Process |
| 3 | establishment of localization | 651 | biological_process, localization | Biological Process |
| 4 | regulation of cellular process | 408 | cellular process, regulation of biological process | Biological Process |
| 4 | cellular aromatic compound metabolic process | 614 | cellular metabolic process | Biological Process |
| 4 | response to drug | 1 | response to chemical stimulus | Biological Process |
| 3 | interspecies interaction between organisms | 21 | multi-organism process | Biological Process |
| 4 | gene expression | 351 | macromolecule metabolic process | Biological Process |
| null | all | 0 |  | Biological Process |
| 4 | macromolecule modification | 358 | macromolecule metabolic process | Biological Process |
| 3 | cellular metabolic process | 1300 | metabolic process, cellular process | Biological Process |
| 3 | cellular response to stimulus | 409 | response to stimulus, single-organism cellular process | Biological Process |
| 4 | protein localization | 185 | macromolecule localization | Biological Process |
| 2 | developmental process | 636 | biological_process | Biological Process |
| null | single-multicellular organism process | 566 | multicellular organismal process, single-organism process | Biological Process |
| 5 | protein modification process | 358 | macromolecule modification, protein metabolic process | Biological Process |
| 3 | protein complex | 790 | macromolecular complex | Cellular Component |
| 2 | cell | 2405 | cellular_component | Cellular Component |
| 5 | cytoplasm | 1340 | intracellular part | Cellular Component |
| 7 | nucleus | 690 | intracellular membrane-bounded organelle | Cellular Component |
| 4 | intracellular | 2046 | cell part | Cellular Component |
| 6 | mitochondrion | 314 | cytoplasmic part, intracellular membrane-bounded organelle | Cellular Component |
| 6 | ribosome | 275 | ribonucleoprotein complex, cytoplasmic part, intracellular non-membrane-bounded organelle | Cellular Component |
| 6 | nucleoplasm | 244 | nuclear part, nuclear lumen | Cellular Component |
| 7 | cytoskeleton | 244 | intracellular non-membrane-bounded organelle | Cellular Component |
| 4 | plasma membrane | 207 | cell part, cell periphery, membrane | Cellular Component |
| 6 | cytosol | 190 | cytoplasmic part | Cellular Component |
| 6 | nucleolus | 129 | nuclear part, nuclear lumen, intracellular non-membrane-bounded organelle | Cellular Component |
| 7 | chromosome | 153 | intracellular non-membrane-bounded organelle | Cellular Component |
| 6 | endoplasmic reticulum | 122 | cytoplasmic part, intracellular membrane-bounded organelle | Cellular Component |
| 2 | organelle | 1602 | cellular_component | Cellular Component |
| 6 | lipid particle | 87 | cytoplasmic part | Cellular Component |
| 6 | Golgi apparatus | 86 | cytoplasmic part, intracellular membrane-bounded organelle | Cellular Component |
| 2 | extracellular region | 112 | cellular_component | Cellular Component |
| 7 | cytoplasmic membrane-bounded vesicle | 53 | cytoplasmic vesicle, intracellular membrane-bounded organelle, membrane-bounded vesicle | Cellular Component |
| 6 | nuclear chromosome | 50 | nuclear part, nuclear lumen, chromosome | Cellular Component |
| 5 | nuclear envelope | 48 | nuclear part, organelle envelope, endomembrane system | Cellular Component |
| 6 | endosome | 38 | cytoplasmic part, intracellular membrane-bounded organelle | Cellular Component |
| 7 | microtubule organizing center | 38 | microtubule cytoskeleton, cytoskeletal part | Cellular Component |
| 4 | extracellular space | 30 | extracellular region part | Cellular Component |
| 6 | vacuole | 48 | cytoplasmic part, intracellular membrane-bounded organelle | Cellular Component |
| 8 | lysosome | 21 | lytic vacuole | Cellular Component |
| 7 | peroxisome | 19 | microbody | Cellular Component |
| 4 | proteinaceous extracellular matrix | 19 | extracellular region part, extracellular matrix | Cellular Component |
| 6 | plastid | 13 | cytoplasmic part, intracellular membrane-bounded organelle | Cellular Component |
| 5 | cell wall | 7 | external encapsulating structure | Cellular Component |
| 5 | cilium | 6 | cell projection, intracellular membrane-bounded organelle | Cellular Component |
| 4 | external encapsulating structure | 11 | cell part, cell periphery | Cellular Component |
| 5 | cell envelope | 1 | envelope | Cellular Component |
| 4 | organelle envelope | 48 | intracellular organelle part, membrane-bounded organelle, envelope | Cellular Component |
| 3 | extracellular region part | 47 | cellular_component, extracellular region | Cellular Component |
| 7 | lytic vacuole | 21 | vacuole | Cellular Component |
| 4 | endomembrane system | 48 | cell part | Cellular Component |
| 3 | membrane-bounded organelle | 1184 | organelle | Cellular Component |
| 3 | vesicle | 53 | organelle | Cellular Component |
| 4 | envelope | 49 | cell part | Cellular Component |
| 6 | intracellular organelle lumen | 334 | intracellular organelle part, organelle lumen | Cellular Component |
| null | all | 0 |  | Cellular Component |
| 2 | extracellular matrix | 19 | cellular_component | Cellular Component |
| 6 | intracellular non-membrane-bounded organelle | 713 | intracellular organelle, non-membrane-bounded organelle | Cellular Component |
| 4 | organelle lumen | 334 | organelle part, membrane-enclosed lumen | Cellular Component |
| 6 | nuclear part | 369 | intracellular organelle part, nucleus | Cellular Component |
| 3 | non-membrane-bounded organelle | 713 | organelle | Cellular Component |
| 6 | intracellular membrane-bounded organelle | 1184 | intracellular organelle, membrane-bounded organelle | Cellular Component |
| 4 | membrane-bounded vesicle | 53 | vesicle | Cellular Component |
| 5 | cytoplasmic part | 910 | intracellular part, cytoplasm | Cellular Component |
| 3 | cell part | 2114 | cell, cellular_component | Cellular Component |
| 6 | cytoskeletal part | 38 | intracellular organelle part, cytoskeleton | Cellular Component |
| 5 | intracellular organelle | 1531 | organelle, intracellular part | Cellular Component |
| 2 | membrane-enclosed lumen | 334 | cellular_component | Cellular Component |
| 5 | ribonucleoprotein complex | 275 | macromolecular complex, intracellular part | Cellular Component |
| 2 | membrane | 207 | cellular_component | Cellular Component |
| 6 | microbody | 19 | cytoplasmic part, intracellular membrane-bounded organelle | Cellular Component |
| 6 | cytoplasmic vesicle | 53 | cytoplasmic part, vesicle, intracellular organelle | Cellular Component |
| 2 | macromolecular complex | 1024 | cellular_component | Cellular Component |
| 4 | intracellular organelle part | 393 | organelle part, intracellular organelle, intracellular part | Cellular Component |
| 7 | nuclear lumen | 334 | nuclear part, intracellular organelle lumen | Cellular Component |
| 8 | microtubule cytoskeleton | 38 | cytoskeleton | Cellular Component |
| 4 | cell projection | 6 | cell part | Cellular Component |
| 4 | cell periphery | 215 | cell part | Cellular Component |
| 3 | organelle part | 393 | cellular_component, organelle | Cellular Component |
| 4 | intracellular part | 1827 | cell part, intracellular | Cellular Component |
| 4 | nucleotide binding | 828 | small molecule binding, nucleoside phosphate binding | Molecular Function |
| 2 | catalytic activity | 2012 | molecular_function | Molecular Function |
| 2 | binding | 2097 | molecular_function | Molecular Function |
| 3 | hydrolase activity | 867 | catalytic activity | Molecular Function |
| 3 | protein binding | 657 | binding | Molecular Function |
| 3 | transferase activity | 592 | catalytic activity | Molecular Function |
| 2 | structural molecule activity | 259 | molecular_function | Molecular Function |
| 2 | transporter activity | 272 | molecular_function | Molecular Function |
| 4 | DNA binding | 192 | nucleic acid binding | Molecular Function |
| 4 | peptidase activity | 189 | hydrolase activity | Molecular Function |
| 4 | RNA binding | 276 | nucleic acid binding | Molecular Function |
| 3 | nucleic acid binding | 592 | heterocyclic compound binding, organic cyclic compound binding | Molecular Function |
| 6 | protein kinase activity | 122 | kinase activity, phosphotransferase activity, alcohol group as acceptor | Molecular Function |
| 2 | enzyme regulator activity | 101 | molecular_function | Molecular Function |
| 5 | translation factor activity, nucleic acid binding | 89 | RNA binding | Molecular Function |
| 6 | calcium ion binding | 87 | metal ion binding | Molecular Function |
| 5 | kinase activity | 208 | transferase activity, transferring phosphorus-containing groups | Molecular Function |
| 3 | lipid binding | 65 | binding | Molecular Function |
| 5 | actin binding | 61 | cytoskeletal protein binding | Molecular Function |
| 2 | receptor activity | 53 | molecular_function | Molecular Function |
| 3 | sequence-specific DNA binding transcription factor activity | 53 | nucleic acid binding transcription factor activity | Molecular Function |
| 7 | phosphoprotein phosphatase activity | 51 | phosphatase activity | Molecular Function |
| 3 | carbohydrate binding | 50 | binding | Molecular Function |
| 2 | electron carrier activity | 48 | molecular_function | Molecular Function |
| 4 | receptor binding | 42 | protein binding | Molecular Function |
| 4 | cytoskeletal protein binding | 95 | protein binding | Molecular Function |
| 5 | nuclease activity | 38 | hydrolase activity, acting on ester bonds | Molecular Function |
| 3 | chromatin binding | 37 | binding | Molecular Function |
| 3 | signal transducer activity | 30 | molecular transducer activity | Molecular Function |
| 8 | motor activity | 29 | nucleoside-triphosphatase activity | Molecular Function |
| null | transcription regulator activity | 28 | obsolete_molecular_function | Molecular Function |
| 2 | antioxidant activity | 19 | molecular_function | Molecular Function |
| 6 | ion channel activity | 16 | substrate-specific channel activity, ion transmembrane transporter activity | Molecular Function |
| 3 | organic cyclic compound binding | 1213 | binding | Molecular Function |
| 5 | carboxylic ester hydrolase activity | 9 | hydrolase activity, acting on ester bonds | Molecular Function |
| 2 | translation regulator activity | 2 | molecular_function | Molecular Function |
| 4 | neurotransmitter transporter activity | 2 | transporter activity | Molecular Function |
| 6 | lead ion binding | 1 | metal ion binding | Molecular Function |
| 3 | small molecule binding | 829 | binding | Molecular Function |
| 3 | oxygen binding | 1 | binding | Molecular Function |
| 4 | substrate-specific transmembrane transporter activity | 16 | substrate-specific transporter activity, transmembrane transporter activity | Molecular Function |
| 5 | hydrolase activity, acting on acid anhydrides, in phosphorus-containing anhydrides | 29 | hydrolase activity, acting on acid anhydrides | Molecular Function |
| 2 | molecular transducer activity | 30 | molecular_function | Molecular Function |
| 5 | ion transmembrane transporter activity | 16 | substrate-specific transmembrane transporter activity | Molecular Function |
| 5 | metal ion binding | 88 | cation binding | Molecular Function |
| 5 | channel activity | 16 | passive transmembrane transporter activity | Molecular Function |
| 5 | phosphoric ester hydrolase activity | 51 | hydrolase activity, acting on ester bonds | Molecular Function |
| 4 | cation binding | 88 | ion binding | Molecular Function |
| 4 | hydrolase activity, acting on acid anhydrides | 29 | hydrolase activity | Molecular Function |
| 4 | hydrolase activity, acting on ester bonds | 98 | hydrolase activity | Molecular Function |
| 3 | transmembrane transporter activity | 16 | transporter activity | Molecular Function |
| 6 | phosphatase activity | 51 | phosphoric ester hydrolase activity | Molecular Function |
| 5 | phosphotransferase activity, alcohol group as acceptor | 122 | transferase activity, transferring phosphorus-containing groups | Molecular Function |
| 2 | nucleic acid binding transcription factor activity | 53 | molecular_function | Molecular Function |
| null | all | 0 |  | Molecular Function |
| 3 | substrate-specific transporter activity | 16 | transporter activity | Molecular Function |
| 3 | ion binding | 88 | binding | Molecular Function |
| 4 | passive transmembrane transporter activity | 16 | transmembrane transporter activity | Molecular Function |
| null | obsolete_molecular_function | 28 |  | Molecular Function |
| null | nucleoside phosphate binding | 828 | heterocyclic compound binding, organic cyclic compound binding | Molecular Function |
| 6 | pyrophosphatase activity | 29 | hydrolase activity, acting on acid anhydrides, in phosphorus-containing anhydrides | Molecular Function |
| null | heterocyclic compound binding | 1203 | binding | Molecular Function |
| 7 | nucleoside-triphosphatase activity | 29 | pyrophosphatase activity | Molecular Function |
| 5 | substrate-specific channel activity | 16 | substrate-specific transmembrane transporter activity, channel activity | Molecular Function |
| 4 | transferase activity, transferring phosphorus-containing groups | 208 | transferase activity | Molecular Function |

**Table S1c.**

| **GO Level** | **Term (Name)** | **#Sequence** | **Parents (Name)** | **Category** |
| --- | --- | --- | --- | --- |
| 2 | metabolic process | 2379 | biological_process | Biological Process |
| 3 | regulation of biological process | 850 | biological regulation, biological_process | Biological Process |
| 3 | multicellular organismal development | 680 | single-multicellular organism process, developmental process | Biological Process |
| 3 | catabolic process | 619 | metabolic process | Biological Process |
| 4 | nucleobase-containing compound metabolic process | 572 | heterocycle metabolic process, primary metabolic process, organic cyclic compound metabolic process, cellular aromatic compound metabolic process, cellular nitrogen compound metabolic process | Biological Process |
| 3 | cellular component organization | 708 | cellular component organization or biogenesis | Biological Process |
| 4 | cell differentiation | 413 | cellular developmental process | Biological Process |
| 4 | transport | 620 | establishment of localization | Biological Process |
| 3 | biosynthetic process | 759 | metabolic process | Biological Process |
| 4 | protein metabolic process | 998 | primary metabolic process, macromolecule metabolic process | Biological Process |
| 6 | translation | 371 | cellular macromolecule biosynthetic process, cellular protein metabolic process, gene expression | Biological Process |
| 4 | signal transduction | 360 | single organism signaling, cell communication, cellular response to stimulus, regulation of cellular process | Biological Process |
| 4 | anatomical structure morphogenesis | 347 | anatomical structure development, developmental process | Biological Process |
| 6 | cellular protein modification process | 344 | cellular protein metabolic process, protein modification process | Biological Process |
| 3 | response to stress | 316 | response to stimulus | Biological Process |
| 3 | cell cycle | 249 | single-organism cellular process | Biological Process |
| 4 | generation of precursor metabolites and energy | 235 | cellular metabolic process | Biological Process |
| 5 | organelle organization | 420 | single-organism cellular process, cellular component organization | Biological Process |
| 2 | reproduction | 217 | biological_process | Biological Process |
| 4 | carbohydrate metabolic process | 216 | primary metabolic process, organic substance metabolic process | Biological Process |
| 6 | cytoskeleton organization | 206 | organelle organization | Biological Process |
| 6 | protein transport | 199 | organic substance transport, establishment of protein localization | Biological Process |
| 4 | embryo development | 163 | single-organism developmental process, multicellular organismal development, anatomical structure development | Biological Process |
| 3 | cell death | 159 | death, single-organism cellular process | Biological Process |
| 4 | lipid metabolic process | 157 | primary metabolic process, organic substance metabolic process, single-organism metabolic process | Biological Process |
| 5 | ion transport | 133 | single-organism transport | Biological Process |
| 3 | response to external stimulus | 111 | response to stimulus | Biological Process |
| 3 | cell-cell signaling | 110 | single organism signaling, cell communication | Biological Process |
| 3 | cellular homeostasis | 110 | homeostatic process, single-organism cellular process | Biological Process |
| 3 | behavior | 100 | response to stimulus | Biological Process |
| 2 | growth | 104 | biological_process | Biological Process |
| 2 | cell proliferation | 89 | single-organism process | Biological Process |
| 3 | response to biotic stimulus | 74 | response to stimulus | Biological Process |
| 6 | DNA metabolic process | 72 | nucleic acid metabolic process, cellular macromolecule metabolic process | Biological Process |
| 3 | response to abiotic stimulus | 68 | response to stimulus | Biological Process |
| 6 | mitochondrion organization | 55 | organelle organization | Biological Process |
| 3 | response to endogenous stimulus | 43 | response to stimulus | Biological Process |
| 3 | secondary metabolic process | 41 | single-organism metabolic process | Biological Process |
| 2 | viral reproduction | 39 | multi-organism cellular process | Biological Process |
| 3 | cell recognition | 30 | single-multicellular organism process, single-organism cellular process | Biological Process |
| 3 | cell communication | 450 | single-organism cellular process | Biological Process |
| 3 | primary metabolic process | 1712 | metabolic process | Biological Process |
| 4 | symbiosis, encompassing mutualism through parasitism | 22 | interspecies interaction between organisms | Biological Process |
| 3 | cell growth | 20 | growth, single-organism cellular process | Biological Process |
| 7 | regulation of gene expression, epigenetic | 19 | regulation of gene expression | Biological Process |
| 5 | cytoplasm organization | 18 | single-organism cellular process, cellular component organization | Biological Process |
| 5 | intracellular signal transduction | 3 | signal transduction | Biological Process |
| 2 | death | 159 | single-organism process | Biological Process |
| 2 | cellular process | 1936 | biological_process | Biological Process |
| 3 | regulation of biological quality | 110 | biological regulation | Biological Process |
| 2 | localization | 620 | biological_process | Biological Process |
| 2 | response to stimulus | 648 | biological_process | Biological Process |
| 3 | organic substance metabolic process | 1697 | metabolic process | Biological Process |
| 3 | cellular developmental process | 413 | developmental process, single-organism cellular process | Biological Process |
| 4 | cellular biosynthetic process | 371 | cellular metabolic process, biosynthetic process | Biological Process |
| null | organic substance biosynthetic process | 371 | organic substance metabolic process, biosynthetic process | Biological Process |
| null | single-organism cellular process | 1038 | single-organism process, cellular process | Biological Process |
| 5 | cellular protein metabolic process | 698 | protein metabolic process, cellular macromolecule metabolic process | Biological Process |
| null | single-organism metabolic process | 186 | metabolic process | Biological Process |
| 3 | anatomical structure development | 398 | developmental process | Biological Process |
| null | multi-organism cellular process | 39 | multi-organism process, cellular process | Biological Process |
| 5 | cellular macromolecule biosynthetic process | 371 | cellular macromolecule metabolic process, cellular biosynthetic process, macromolecule biosynthetic process | Biological Process |
| 4 | cellular macromolecule metabolic process | 744 | cellular metabolic process, macromolecule metabolic process | Biological Process |
| 4 | regulation of metabolic process | 19 | regulation of biological process, metabolic process | Biological Process |
| 2 | cellular component organization or biogenesis | 708 | biological_process | Biological Process |
| 2 | multi-organism process | 44 | biological_process | Biological Process |
| 6 | regulation of gene expression | 19 | gene expression, regulation of macromolecule metabolic process | Biological Process |
| 4 | heterocycle metabolic process | 572 | cellular metabolic process | Biological Process |
| 5 | organic substance transport | 199 | transport | Biological Process |
| 5 | establishment of protein localization | 199 | establishment of localization, protein localization | Biological Process |
| null | single organism signaling | 431 | single-organism process, signaling | Biological Process |
| 5 | nucleic acid metabolic process | 72 | macromolecule metabolic process, nucleobase-containing compound metabolic process | Biological Process |
| 5 | regulation of macromolecule metabolic process | 19 | macromolecule metabolic process, regulation of metabolic process | Biological Process |
| null | single-organism process | 1285 | biological_process | Biological Process |
| 3 | macromolecule metabolic process | 1054 | organic substance metabolic process | Biological Process |
| 4 | homeostatic process | 110 | regulation of biological quality | Biological Process |
| 4 | cellular nitrogen compound metabolic process | 572 | cellular metabolic process, nitrogen compound metabolic process | Biological Process |
| 2 | signaling | 431 | biological_process | Biological Process |
| null | single-organism transport | 133 | single-organism process, transport | Biological Process |
| null | single-organism developmental process | 163 | single-organism process, developmental process | Biological Process |
| null | organic cyclic compound metabolic process | 572 | organic substance metabolic process | Biological Process |
| 2 | multicellular organismal process | 680 | biological_process | Biological Process |
| 4 | macromolecule biosynthetic process | 371 | organic substance biosynthetic process, macromolecule metabolic process | Biological Process |
| 2 | biological regulation | 879 | biological_process | Biological Process |
| 3 | macromolecule localization | 199 | localization | Biological Process |
| 3 | nitrogen compound metabolic process | 572 | metabolic process | Biological Process |
| 3 | establishment of localization | 620 | biological_process, localization | Biological Process |
| 4 | regulation of cellular process | 360 | cellular process, regulation of biological process | Biological Process |
| 4 | cellular aromatic compound metabolic process | 572 | cellular metabolic process | Biological Process |
| 3 | interspecies interaction between organisms | 22 | multi-organism process | Biological Process |
| 4 | gene expression | 390 | macromolecule metabolic process | Biological Process |
| null | all | 0 |  | Biological Process |
| 4 | macromolecule modification | 344 | macromolecule metabolic process | Biological Process |
| 3 | cellular metabolic process | 1322 | metabolic process, cellular process | Biological Process |
| 3 | cellular response to stimulus | 360 | response to stimulus, single-organism cellular process | Biological Process |
| 4 | protein localization | 199 | macromolecule localization | Biological Process |
| 2 | developmental process | 741 | biological_process | Biological Process |
| null | single-multicellular organism process | 680 | multicellular organismal process, single-organism process | Biological Process |
| 5 | protein modification process | 344 | macromolecule modification, protein metabolic process | Biological Process |
| 3 | protein complex | 765 | macromolecular complex | Cellular Component |
| 2 | cell | 2588 | cellular_component | Cellular Component |
| 5 | cytoplasm | 1564 | intracellular part | Cellular Component |
| 6 | mitochondrion | 465 | cytoplasmic part, intracellular membrane-bounded organelle | Cellular Component |
| 7 | nucleus | 642 | intracellular membrane-bounded organelle | Cellular Component |
| 4 | intracellular | 2204 | cell part | Cellular Component |
| 7 | cytoskeleton | 317 | intracellular non-membrane-bounded organelle | Cellular Component |
| 6 | ribosome | 242 | ribonucleoprotein complex, cytoplasmic part, intracellular non-membrane-bounded organelle | Cellular Component |
| 6 | cytosol | 232 | cytoplasmic part | Cellular Component |
| 4 | plasma membrane | 219 | cell part, cell periphery, membrane | Cellular Component |
| 6 | nucleoplasm | 159 | nuclear part, nuclear lumen | Cellular Component |
| 6 | lipid particle | 144 | cytoplasmic part | Cellular Component |
| 6 | endoplasmic reticulum | 137 | cytoplasmic part, intracellular membrane-bounded organelle | Cellular Component |
| 6 | nucleolus | 103 | nuclear part, nuclear lumen, intracellular non-membrane-bounded organelle | Cellular Component |
| 2 | organelle | 1728 | cellular_component | Cellular Component |
| 6 | Golgi apparatus | 97 | cytoplasmic part, intracellular membrane-bounded organelle | Cellular Component |
| 2 | extracellular region | 153 | cellular_component | Cellular Component |
| 7 | chromosome | 107 | intracellular non-membrane-bounded organelle | Cellular Component |
| 7 | cytoplasmic membrane-bounded vesicle | 66 | cytoplasmic vesicle, intracellular membrane-bounded organelle, membrane-bounded vesicle | Cellular Component |
| 7 | microtubule organizing center | 56 | microtubule cytoskeleton, cytoskeletal part | Cellular Component |
| 4 | extracellular space | 49 | extracellular region part | Cellular Component |
| 6 | endosome | 35 | cytoplasmic part, intracellular membrane-bounded organelle | Cellular Component |
| 5 | nuclear envelope | 34 | nuclear part, organelle envelope, endomembrane system | Cellular Component |
| 6 | nuclear chromosome | 33 | nuclear part, nuclear lumen, chromosome | Cellular Component |
| 6 | vacuole | 53 | cytoplasmic part, intracellular membrane-bounded organelle | Cellular Component |
| 8 | lysosome | 25 | lytic vacuole | Cellular Component |
| 7 | peroxisome | 23 | microbody | Cellular Component |
| 4 | proteinaceous extracellular matrix | 14 | extracellular region part, extracellular matrix | Cellular Component |
| 5 | cilium | 10 | cell projection, intracellular membrane-bounded organelle | Cellular Component |
| 6 | plastid | 3 | cytoplasmic part, intracellular membrane-bounded organelle | Cellular Component |
| 6 | cytoplasmic chromosome | 1 | cytoplasmic part, chromosome | Cellular Component |
| 4 | organelle envelope | 34 | intracellular organelle part, membrane-bounded organelle, envelope | Cellular Component |
| 3 | extracellular region part | 61 | cellular_component, extracellular region | Cellular Component |
| 7 | lytic vacuole | 25 | vacuole | Cellular Component |
| 4 | endomembrane system | 34 | cell part | Cellular Component |
| 3 | membrane-bounded organelle | 1307 | organelle | Cellular Component |
| 3 | vesicle | 66 | organelle | Cellular Component |
| 4 | envelope | 34 | cell part | Cellular Component |
| 6 | intracellular organelle lumen | 241 | intracellular organelle part, organelle lumen | Cellular Component |
| null | all | 0 |  | Cellular Component |
| 2 | extracellular matrix | 14 | cellular_component | Cellular Component |
| 6 | intracellular non-membrane-bounded organelle | 695 | intracellular organelle, non-membrane-bounded organelle | Cellular Component |
| 4 | organelle lumen | 241 | organelle part, membrane-enclosed lumen | Cellular Component |
| 6 | nuclear part | 272 | intracellular organelle part, nucleus | Cellular Component |
| 3 | non-membrane-bounded organelle | 695 | organelle | Cellular Component |
| 6 | intracellular membrane-bounded organelle | 1307 | intracellular organelle, membrane-bounded organelle | Cellular Component |
| 4 | membrane-bounded vesicle | 66 | vesicle | Cellular Component |
| 5 | cytoplasmic part | 1098 | intracellular part, cytoplasm | Cellular Component |
| 3 | cell part | 2276 | cell, cellular_component | Cellular Component |
| 6 | cytoskeletal part | 56 | intracellular organelle part, cytoskeleton | Cellular Component |
| 5 | intracellular organelle | 1663 | organelle, intracellular part | Cellular Component |
| 2 | membrane-enclosed lumen | 241 | cellular_component | Cellular Component |
| 5 | ribonucleoprotein complex | 242 | macromolecular complex, intracellular part | Cellular Component |
| 2 | membrane | 219 | cellular_component | Cellular Component |
| 6 | microbody | 23 | cytoplasmic part, intracellular membrane-bounded organelle | Cellular Component |
| 6 | cytoplasmic vesicle | 66 | cytoplasmic part, vesicle, intracellular organelle | Cellular Component |
| 2 | macromolecular complex | 1003 | cellular_component | Cellular Component |
| 4 | intracellular organelle part | 312 | organelle part, intracellular organelle, intracellular part | Cellular Component |
| 7 | nuclear lumen | 241 | nuclear part, intracellular organelle lumen | Cellular Component |
| 8 | microtubule cytoskeleton | 56 | cytoskeleton | Cellular Component |
| 4 | cell projection | 10 | cell part | Cellular Component |
| 4 | cell periphery | 219 | cell part | Cellular Component |
| 4 | intracellular part | 1985 | cell part, intracellular | Cellular Component |
| 3 | organelle part | 312 | cellular_component, organelle | Cellular Component |
| 2 | catalytic activity | 2538 | molecular_function | Molecular Function |
| 4 | nucleotide binding | 865 | small molecule binding, nucleoside phosphate binding | Molecular Function |
| 2 | binding | 2272 | molecular_function | Molecular Function |
| 3 | hydrolase activity | 1112 | catalytic activity | Molecular Function |
| 3 | protein binding | 708 | binding | Molecular Function |
| 3 | transferase activity | 630 | catalytic activity | Molecular Function |
| 2 | structural molecule activity | 315 | molecular_function | Molecular Function |
| 2 | transporter activity | 331 | molecular_function | Molecular Function |
| 4 | peptidase activity | 283 | hydrolase activity | Molecular Function |
| 4 | RNA binding | 284 | nucleic acid binding | Molecular Function |
| 4 | DNA binding | 177 | nucleic acid binding | Molecular Function |
| 3 | nucleic acid binding | 590 | heterocyclic compound binding, organic cyclic compound binding | Molecular Function |
| 6 | protein kinase activity | 124 | kinase activity, phosphotransferase activity, alcohol group as acceptor | Molecular Function |
| 2 | enzyme regulator activity | 104 | molecular_function | Molecular Function |
| 5 | translation factor activity, nucleic acid binding | 100 | RNA binding | Molecular Function |
| 2 | electron carrier activity | 90 | molecular_function | Molecular Function |
| 5 | kinase activity | 212 | transferase activity, transferring phosphorus-containing groups | Molecular Function |
| 6 | calcium ion binding | 83 | metal ion binding | Molecular Function |
| 3 | lipid binding | 82 | binding | Molecular Function |
| 5 | actin binding | 70 | cytoskeletal protein binding | Molecular Function |
| 2 | receptor activity | 61 | molecular_function | Molecular Function |
| 3 | carbohydrate binding | 56 | binding | Molecular Function |
| 3 | sequence-specific DNA binding transcription factor activity | 53 | nucleic acid binding transcription factor activity | Molecular Function |
| 4 | receptor binding | 52 | protein binding | Molecular Function |
| 7 | phosphoprotein phosphatase activity | 46 | phosphatase activity | Molecular Function |
| 4 | cytoskeletal protein binding | 104 | protein binding | Molecular Function |
| null | transcription regulator activity | 33 | obsolete_molecular_function | Molecular Function |
| 3 | signal transducer activity | 31 | molecular transducer activity | Molecular Function |
| 3 | chromatin binding | 30 | binding | Molecular Function |
| 6 | ion channel activity | 30 | substrate-specific channel activity, ion transmembrane transporter activity | Molecular Function |
| 2 | antioxidant activity | 29 | molecular_function | Molecular Function |
| 5 | nuclease activity | 28 | hydrolase activity, acting on ester bonds | Molecular Function |
| 8 | motor activity | 26 | nucleoside-triphosphatase activity | Molecular Function |
| 5 | carboxylic ester hydrolase activity | 17 | hydrolase activity, acting on ester bonds | Molecular Function |
| 3 | organic cyclic compound binding | 1247 | binding | Molecular Function |
| 4 | neurotransmitter transporter activity | 3 | transporter activity | Molecular Function |
| 2 | translation regulator activity | 1 | molecular_function | Molecular Function |
| 2 | protein tag | 1 | molecular_function | Molecular Function |
| 6 | 7SK snRNA binding | 1 | snRNA binding | Molecular Function |
| 7 | inositol phosphate phosphatase activity | 1 | phosphatase activity | Molecular Function |
| 3 | oxygen binding | 1 | binding | Molecular Function |
| 4 | substrate-specific transmembrane transporter activity | 30 | substrate-specific transporter activity, transmembrane transporter activity | Molecular Function |
| 5 | hydrolase activity, acting on acid anhydrides, in phosphorus-containing anhydrides | 26 | hydrolase activity, acting on acid anhydrides | Molecular Function |
| 2 | molecular transducer activity | 31 | molecular_function | Molecular Function |
| 5 | ion transmembrane transporter activity | 30 | substrate-specific transmembrane transporter activity | Molecular Function |
| 5 | metal ion binding | 83 | cation binding | Molecular Function |
| 5 | channel activity | 30 | passive transmembrane transporter activity | Molecular Function |
| 5 | phosphoric ester hydrolase activity | 47 | hydrolase activity, acting on ester bonds | Molecular Function |
| 4 | cation binding | 83 | ion binding | Molecular Function |
| 4 | hydrolase activity, acting on acid anhydrides | 26 | hydrolase activity | Molecular Function |
| 4 | hydrolase activity, acting on ester bonds | 92 | hydrolase activity | Molecular Function |
| 3 | transmembrane transporter activity | 30 | transporter activity | Molecular Function |
| 5 | snRNA binding | 1 | RNA binding | Molecular Function |
| 6 | phosphatase activity | 47 | phosphoric ester hydrolase activity | Molecular Function |
| 5 | phosphotransferase activity, alcohol group as acceptor | 124 | transferase activity, transferring phosphorus-containing groups | Molecular Function |
| 3 | small molecule binding | 865 | binding | Molecular Function |
| 2 | nucleic acid binding transcription factor activity | 53 | molecular_function | Molecular Function |
| null | all | 0 |  | Molecular Function |
| 3 | substrate-specific transporter activity | 30 | transporter activity | Molecular Function |
| 3 | ion binding | 83 | binding | Molecular Function |
| 4 | passive transmembrane transporter activity | 30 | transmembrane transporter activity | Molecular Function |
| null | obsolete_molecular_function | 33 |  | Molecular Function |
| null | nucleoside phosphate binding | 865 | heterocyclic compound binding, organic cyclic compound binding | Molecular Function |
| 6 | pyrophosphatase activity | 26 | hydrolase activity, acting on acid anhydrides, in phosphorus-containing anhydrides | Molecular Function |
| null | heterocyclic compound binding | 1243 | binding | Molecular Function |
| 7 | nucleoside-triphosphatase activity | 26 | pyrophosphatase activity | Molecular Function |
| 5 | substrate-specific channel activity | 30 | substrate-specific transmembrane transporter activity, channel activity | Molecular Function |
| 4 | transferase activity, transferring phosphorus-containing groups | 212 | transferase activity | Molecular Function |

**Table S1d.**

|  |  |  |  |  |  |
| --- | --- | --- | --- | --- | --- |
| **GO Level** | **Term (Name)** | **#Sequence** | **Parents (Name)** | **Category** |  |
| 2 | metabolic process | 1862 | biological_process | Biological Process |  |
| null | single-organism process | 1001 | biological_process | Biological Process |  |
| null | single-organism cellular process | 810 | single-organism process, cellular process | Biological Process |  |
| 3 | primary metabolic process | 1402 | metabolic process | Biological Process |  |
| 2 | cellular process | 1567 | biological_process | Biological Process |  |
| 3 | regulation of biological process | 644 | biological regulation, biological_process | Biological Process |  |
| 2 | developmental process | 544 | biological_process | Biological Process |  |
| 3 | multicellular organismal development | 495 | single-multicellular organism process, developmental process | Biological Process |  |
| 3 | cellular component organization | 557 | cellular component organization or biogenesis | Biological Process |  |
| 3 | organic substance metabolic process | 1384 | metabolic process | Biological Process |  |
| 4 | protein metabolic process | 817 | primary metabolic process, macromolecule metabolic process | Biological Process |  |
| 4 | nucleobase-containing compound metabolic process | 512 | heterocycle metabolic process, primary metabolic process, organic cyclic compound metabolic process, cellular aromatic compound metabolic process, cellular nitrogen compound metabolic process | Biological Process |  |
| 4 | transport | 506 | establishment of localization | Biological Process |  |
| 3 | catabolic process | 428 | metabolic process | Biological Process |  |
| 3 | cellular metabolic process | 1123 | metabolic process, cellular process | Biological Process |  |
| 2 | response to stimulus | 477 | biological_process | Biological Process |  |
| 2 | biological regulation | 655 | biological_process | Biological Process |  |
| 5 | cellular protein metabolic process | 608 | protein metabolic process, cellular macromolecule metabolic process | Biological Process |  |
| 3 | biosynthetic process | 641 | metabolic process | Biological Process |  |
| 5 | organelle organization | 396 | single-organism cellular process, cellular component organization | Biological Process |  |
| 4 | cell differentiation | 355 | cellular developmental process | Biological Process |  |
| 6 | translation | 352 | cellular macromolecule biosynthetic process, cellular protein metabolic process, gene expression | Biological Process |  |
| 3 | macromolecule metabolic process | 889 | organic substance metabolic process | Biological Process |  |
| null | single-multicellular organism process | 495 | multicellular organismal process, single-organism process | Biological Process |  |
| 2 | cellular component organization or biogenesis | 557 | biological_process | Biological Process |  |
| 4 | heterocycle metabolic process | 512 | cellular metabolic process | Biological Process |  |
| 4 | cellular nitrogen compound metabolic process | 512 | cellular metabolic process, nitrogen compound metabolic process | Biological Process |  |
| null | organic cyclic compound metabolic process | 512 | organic substance metabolic process | Biological Process |  |
| 4 | cellular aromatic compound metabolic process | 512 | cellular metabolic process | Biological Process |  |
| 4 | cellular macromolecule metabolic process | 677 | cellular metabolic process, macromolecule metabolic process | Biological Process |  |
| 6 | cellular protein modification process | 265 | cellular protein metabolic process, protein modification process | Biological Process |  |
| 3 | establishment of localization | 506 | biological_process, localization | Biological Process |  |
| 4 | anatomical structure morphogenesis | 260 | anatomical structure development, developmental process | Biological Process |  |
| 3 | cell cycle | 257 | single-organism cellular process | Biological Process |  |
| 3 | response to stress | 240 | response to stimulus | Biological Process |  |
| 4 | signal transduction | 232 | single organism signaling, cell communication, cellular response to stimulus, regulation of cellular process | Biological Process |  |
| 3 | anatomical structure development | 303 | developmental process | Biological Process |  |
| 4 | gene expression | 367 | macromolecule metabolic process | Biological Process |  |
| 6 | cytoskeleton organization | 216 | organelle organization | Biological Process |  |
| 3 | cellular developmental process | 355 | developmental process, single-organism cellular process | Biological Process |  |
| 5 | cellular macromolecule biosynthetic process | 352 | cellular macromolecule metabolic process, cellular biosynthetic process, macromolecule biosynthetic process | Biological Process |  |
| 3 | cell communication | 308 | single-organism cellular process | Biological Process |  |
| 2 | multicellular organismal process | 495 | biological_process | Biological Process |  |
| null | single organism signaling | 292 | single-organism process, signaling | Biological Process |  |
| 2 | reproduction | 184 | biological_process | Biological Process |  |
| 3 | nitrogen compound metabolic process | 512 | metabolic process | Biological Process |  |
| 4 | generation of precursor metabolites and energy | 164 | cellular metabolic process | Biological Process |  |
| 5 | protein modification process | 265 | macromolecule modification, protein metabolic process | Biological Process |  |
| 2 | localization | 506 | biological_process | Biological Process |  |
| 6 | protein transport | 153 | organic substance transport, establishment of protein localization | Biological Process |  |
| 4 | regulation of cellular process | 232 | cellular process, regulation of biological process | Biological Process |  |
| 3 | cellular response to stimulus | 232 | response to stimulus, single-organism cellular process | Biological Process |  |
| 4 | carbohydrate metabolic process | 136 | primary metabolic process, organic substance metabolic process | Biological Process |  |
| 5 | ion transport | 128 | single-organism transport | Biological Process |  |
| 4 | cellular biosynthetic process | 352 | cellular metabolic process, biosynthetic process | Biological Process |  |
| 4 | macromolecule biosynthetic process | 352 | organic substance biosynthetic process, macromolecule metabolic process | Biological Process |  |
| 4 | embryo development | 122 | single-organism developmental process, multicellular organismal development, anatomical structure development | Biological Process |  |
| 2 | signaling | 292 | biological_process | Biological Process |  |
| 4 | lipid metabolic process | 111 | primary metabolic process, organic substance metabolic process, single-organism metabolic process | Biological Process |  |
| 3 | cell death | 109 | death, single-organism cellular process | Biological Process |  |
| 4 | macromolecule modification | 265 | macromolecule metabolic process | Biological Process |  |
| 6 | DNA metabolic process | 95 | nucleic acid metabolic process, cellular macromolecule metabolic process | Biological Process |  |
| 2 | cell proliferation | 95 | single-organism process | Biological Process |  |
| 5 | organic substance transport | 153 | transport | Biological Process |  |
| 5 | establishment of protein localization | 153 | establishment of localization, protein localization | Biological Process |  |
| 3 | cell-cell signaling | 85 | single organism signaling, cell communication | Biological Process |  |
| null | single-organism metabolic process | 129 | metabolic process | Biological Process |  |
| null | single-organism transport | 128 | single-organism process, transport | Biological Process |  |
| null | organic substance biosynthetic process | 352 | organic substance metabolic process, biosynthetic process | Biological Process |  |
| 3 | behavior | 74 | response to stimulus | Biological Process |  |
| 3 | response to external stimulus | 74 | response to stimulus | Biological Process |  |
| null | single-organism developmental process | 122 | single-organism process, developmental process | Biological Process |  |
| 3 | cellular homeostasis | 70 | homeostatic process, single-organism cellular process | Biological Process |  |
| 2 | death | 110 | single-organism process | Biological Process |  |
| 2 | growth | 67 | biological_process | Biological Process |  |
| 5 | nucleic acid metabolic process | 95 | macromolecule metabolic process, nucleobase-containing compound metabolic process | Biological Process |  |
| 4 | protein localization | 153 | macromolecule localization | Biological Process |  |
| 3 | response to abiotic stimulus | 55 | response to stimulus | Biological Process |  |
| 4 | homeostatic process | 70 | regulation of biological quality | Biological Process |  |
| 2 | viral reproduction | 39 | multi-organism cellular process | Biological Process |  |
| 6 | mitochondrion organization | 38 | organelle organization | Biological Process |  |
| 3 | response to biotic stimulus | 36 | response to stimulus | Biological Process |  |
| 3 | macromolecule localization | 153 | localization | Biological Process |  |
| 3 | response to endogenous stimulus | 30 | response to stimulus | Biological Process |  |
| 3 | secondary metabolic process | 27 | single-organism metabolic process | Biological Process |  |
| 3 | regulation of biological quality | 70 | biological regulation | Biological Process |  |
| null | multi-organism cellular process | 39 | multi-organism process, cellular process | Biological Process |  |
| 2 | multi-organism process | 43 | biological_process | Biological Process |  |
| 4 | symbiosis, encompassing mutualism through parasitism | 21 | interspecies interaction between organisms | Biological Process |  |
| 3 | cell growth | 20 | growth, single-organism cellular process | Biological Process |  |
| 7 | regulation of gene expression, epigenetic | 19 | regulation of gene expression | Biological Process |  |
| 5 | cytoplasm organization | 17 | single-organism cellular process, cellular component organization | Biological Process |  |
| 3 | cell recognition | 15 | single-multicellular organism process, single-organism cellular process | Biological Process |  |
| 3 | interspecies interaction between organisms | 21 | multi-organism process | Biological Process |  |
| 6 | regulation of gene expression | 19 | gene expression, regulation of macromolecule metabolic process | Biological Process |  |
| 5 | regulation of macromolecule metabolic process | 19 | macromolecule metabolic process, regulation of metabolic process | Biological Process |  |
| 4 | regulation of metabolic process | 19 | regulation of biological process, metabolic process | Biological Process |  |
| 5 | intracellular signal transduction | 2 | signal transduction | Biological Process |  |
| 2 | cell | 2076 | cellular_component | Cellular Component |  |
| 4 | intracellular part | 1679 | cell part, intracellular | Cellular Component |  |
| 5 | cytoplasm | 1292 | intracellular part | Cellular Component |  |
| 4 | intracellular | 1834 | cell part | Cellular Component |  |
| 3 | cell part | 1876 | cell, cellular_component | Cellular Component |  |
| 5 | cytoplasmic part | 898 | intracellular part, cytoplasm | Cellular Component |  |
| 6 | intracellular membrane-bounded organelle | 1057 | intracellular organelle, membrane-bounded organelle | Cellular Component |  |
| 3 | protein complex | 642 | macromolecular complex | Cellular Component |  |
| 5 | intracellular organelle | 1402 | organelle, intracellular part | Cellular Component |  |
| 7 | nucleus | 579 | intracellular membrane-bounded organelle | Cellular Component |  |
| 2 | macromolecular complex | 859 | cellular_component | Cellular Component |  |
| 2 | organelle | 1434 | cellular_component | Cellular Component |  |
| 6 | intracellular non-membrane-bounded organelle | 636 | intracellular organelle, non-membrane-bounded organelle | Cellular Component |  |
| 3 | membrane-bounded organelle | 1057 | organelle | Cellular Component |  |
| 6 | mitochondrion | 315 | cytoplasmic part, intracellular membrane-bounded organelle | Cellular Component |  |
| 7 | cytoskeleton | 276 | intracellular non-membrane-bounded organelle | Cellular Component |  |
| 3 | non-membrane-bounded organelle | 636 | organelle | Cellular Component |  |
| 6 | ribosome | 219 | ribonucleoprotein complex, cytoplasmic part, intracellular non-membrane-bounded organelle | Cellular Component |  |
| 6 | cytosol | 204 | cytoplasmic part | Cellular Component |  |
| 6 | nuclear part | 270 | intracellular organelle part, nucleus | Cellular Component |  |
| 7 | nuclear lumen | 239 | nuclear part, intracellular organelle lumen | Cellular Component |  |
| 4 | plasma membrane | 161 | cell part, cell periphery, membrane | Cellular Component |  |
| 6 | nucleoplasm | 141 | nuclear part, nuclear lumen | Cellular Component |  |
| 5 | ribonucleoprotein complex | 219 | macromolecular complex, intracellular part | Cellular Component |  |
| 4 | intracellular organelle part | 297 | organelle part, intracellular organelle, intracellular part | Cellular Component |  |
| 6 | lipid particle | 126 | cytoplasmic part | Cellular Component |  |
| 6 | endoplasmic reticulum | 110 | cytoplasmic part, intracellular membrane-bounded organelle | Cellular Component |  |
| 6 | intracellular organelle lumen | 239 | intracellular organelle part, organelle lumen | Cellular Component |  |
| 6 | nucleolus | 102 | nuclear part, nuclear lumen, intracellular non-membrane-bounded organelle | Cellular Component |  |
| 4 | cell periphery | 161 | cell part | Cellular Component |  |
| 2 | membrane | 161 | cellular_component | Cellular Component |  |
| 7 | chromosome | 101 | intracellular non-membrane-bounded organelle | Cellular Component |  |
| 6 | Golgi apparatus | 82 | cytoplasmic part, intracellular membrane-bounded organelle | Cellular Component |  |
| 3 | organelle part | 297 | cellular_component, organelle | Cellular Component |  |
| 2 | extracellular region | 86 | cellular_component | Cellular Component |  |
| 4 | organelle lumen | 239 | organelle part, membrane-enclosed lumen | Cellular Component |  |
| 7 | cytoplasmic membrane-bounded vesicle | 61 | cytoplasmic vesicle, intracellular membrane-bounded organelle, membrane-bounded vesicle | Cellular Component |  |
| 6 | nuclear chromosome | 43 | nuclear part, nuclear lumen, chromosome | Cellular Component |  |
| 5 | nuclear envelope | 43 | nuclear part, organelle envelope, endomembrane system | Cellular Component |  |
| 2 | membrane-enclosed lumen | 239 | cellular_component | Cellular Component |  |
| 4 | membrane-bounded vesicle | 61 | vesicle | Cellular Component |  |
| 6 | cytoplasmic vesicle | 61 | cytoplasmic part, vesicle, intracellular organelle | Cellular Component |  |
| 7 | microtubule organizing center | 36 | microtubule cytoskeleton, cytoskeletal part | Cellular Component |  |
| 6 | vacuole | 43 | cytoplasmic part, intracellular membrane-bounded organelle | Cellular Component |  |
| 6 | endosome | 32 | cytoplasmic part, intracellular membrane-bounded organelle | Cellular Component |  |
| 4 | organelle envelope | 43 | intracellular organelle part, membrane-bounded organelle, envelope | Cellular Component |  |
| 4 | endomembrane system | 43 | cell part | Cellular Component |  |
| 3 | vesicle | 61 | organelle | Cellular Component |  |
| 6 | cytoskeletal part | 36 | intracellular organelle part, cytoskeleton | Cellular Component |  |
| 8 | microtubule cytoskeleton | 36 | cytoskeleton | Cellular Component |  |
| 8 | lysosome | 19 | lytic vacuole | Cellular Component |  |
| 7 | peroxisome | 17 | microbody | Cellular Component |  |
| 4 | extracellular space | 16 | extracellular region part | Cellular Component |  |
| 4 | envelope | 43 | cell part | Cellular Component |  |
| 3 | extracellular region part | 20 | cellular_component, extracellular region | Cellular Component |  |
| 7 | lytic vacuole | 19 | vacuole | Cellular Component |  |
| 6 | microbody | 17 | cytoplasmic part, intracellular membrane-bounded organelle | Cellular Component |  |
| 4 | proteinaceous extracellular matrix | 6 | extracellular region part, extracellular matrix | Cellular Component |  |
| 6 | plastid | 5 | cytoplasmic part, intracellular membrane-bounded organelle | Cellular Component |  |
| 5 | cilium | 4 | cell projection, intracellular membrane-bounded organelle | Cellular Component |  |
| 2 | extracellular matrix | 6 | cellular_component | Cellular Component |  |
| 4 | cell projection | 4 | cell part | Cellular Component |  |
| 5 | cell wall | 1 | external encapsulating structure | Cellular Component |  |
| 4 | external encapsulating structure | 1 | cell part, cell periphery | Cellular Component |  |
| 2 | binding | 1760 | molecular_function | Molecular Function |  |
| 2 | catalytic activity | 1766 | molecular_function | Molecular Function |  |
| 4 | nucleotide binding | 683 | small molecule binding, nucleoside phosphate binding | Molecular Function |  |
| 3 | hydrolase activity | 744 | catalytic activity | Molecular Function |  |
| 3 | protein binding | 567 | binding | Molecular Function |  |
| 3 | organic cyclic compound binding | 1016 | binding | Molecular Function |  |
| null | heterocyclic compound binding | 1010 | binding | Molecular Function |  |
| 3 | small molecule binding | 683 | binding | Molecular Function |  |
| null | nucleoside phosphate binding | 683 | heterocyclic compound binding, organic cyclic compound binding | Molecular Function |  |
| 3 | transferase activity | 470 | catalytic activity | Molecular Function |  |
| 3 | nucleic acid binding | 506 | heterocyclic compound binding, organic cyclic compound binding | Molecular Function |  |
| 2 | structural molecule activity | 280 | molecular_function | Molecular Function |  |
| 2 | transporter activity | 255 | molecular_function | Molecular Function |  |
| 4 | RNA binding | 256 | nucleic acid binding | Molecular Function |  |
| 4 | peptidase activity | 174 | hydrolase activity | Molecular Function |  |
| 4 | DNA binding | 159 | nucleic acid binding | Molecular Function |  |
| 5 | kinase activity | 159 | transferase activity, transferring phosphorus-containing groups | Molecular Function |  |
| 5 | translation factor activity, nucleic acid binding | 104 | RNA binding | Molecular Function |  |
| 4 | cytoskeletal protein binding | 112 | protein binding | Molecular Function |  |
| 6 | protein kinase activity | 94 | kinase activity, phosphotransferase activity, alcohol group as acceptor | Molecular Function |  |
| 2 | enzyme regulator activity | 84 | molecular_function | Molecular Function |  |
| 6 | calcium ion binding | 78 | metal ion binding | Molecular Function |  |
| 5 | actin binding | 76 | cytoskeletal protein binding | Molecular Function |  |
| 4 | transferase activity, transferring phosphorus-containing groups | 159 | transferase activity | Molecular Function |  |
| 3 | lipid binding | 66 | binding | Molecular Function |  |
| 2 | electron carrier activity | 58 | molecular_function | Molecular Function |  |
| 5 | phosphotransferase activity, alcohol group as acceptor | 94 | transferase activity, transferring phosphorus-containing groups | Molecular Function |  |
| 5 | metal ion binding | 78 | cation binding | Molecular Function |  |
| 7 | phosphoprotein phosphatase activity | 42 | phosphatase activity | Molecular Function |  |
| 3 | sequence-specific DNA binding transcription factor activity | 35 | nucleic acid binding transcription factor activity | Molecular Function |  |
| 4 | receptor binding | 34 | protein binding | Molecular Function |  |
| 8 | motor activity | 30 | nucleoside-triphosphatase activity | Molecular Function |  |
| 4 | cation binding | 78 | ion binding | Molecular Function |  |
| 4 | hydrolase activity, acting on ester bonds | 73 | hydrolase activity | Molecular Function |  |
| 6 | phosphatase activity | 42 | phosphoric ester hydrolase activity | Molecular Function |  |
| 3 | chromatin binding | 25 | binding | Molecular Function |  |
| 5 | nuclease activity | 24 | hydrolase activity, acting on ester bonds | Molecular Function |  |
| null | transcription regulator activity | 22 | obsolete_molecular_function | Molecular Function |  |
| 3 | carbohydrate binding | 22 | binding | Molecular Function |  |
| 2 | receptor activity | 21 | molecular_function | Molecular Function |  |
| 2 | nucleic acid binding transcription factor activity | 35 | molecular_function | Molecular Function |  |
| 7 | nucleoside-triphosphatase activity | 30 | pyrophosphatase activity | Molecular Function |  |
| 6 | ion channel activity | 18 | substrate-specific channel activity, ion transmembrane transporter activity | Molecular Function |  |
| 3 | ion binding | 78 | binding | Molecular Function |  |
| 5 | phosphoric ester hydrolase activity | 42 | hydrolase activity, acting on ester bonds | Molecular Function |  |
| null | obsolete_molecular_function | 22 |  | Molecular Function |  |
| 2 | antioxidant activity | 12 | molecular_function | Molecular Function |  |
| 3 | signal transducer activity | 12 | molecular transducer activity | Molecular Function |  |
| 5 | ion transmembrane transporter activity | 18 | substrate-specific transmembrane transporter activity | Molecular Function |  |
| 6 | pyrophosphatase activity | 30 | hydrolase activity, acting on acid anhydrides, in phosphorus-containing anhydrides | Molecular Function |  |
| 5 | substrate-specific channel activity | 18 | substrate-specific transmembrane transporter activity, channel activity | Molecular Function |  |
| 2 | molecular transducer activity | 12 | molecular_function | Molecular Function |  |
| 5 | carboxylic ester hydrolase activity | 7 | hydrolase activity, acting on ester bonds | Molecular Function |  |
| 4 | substrate-specific transmembrane transporter activity | 18 | substrate-specific transporter activity, transmembrane transporter activity | Molecular Function |  |
| 5 | hydrolase activity, acting on acid anhydrides, in phosphorus-containing anhydrides | 30 | hydrolase activity, acting on acid anhydrides | Molecular Function |  |
| 5 | channel activity | 18 | passive transmembrane transporter activity | Molecular Function |  |
| 2 | nutrient reservoir activity | 6 | molecular_function | Molecular Function |  |
| 4 | hydrolase activity, acting on acid anhydrides | 30 | hydrolase activity | Molecular Function |  |
| 3 | transmembrane transporter activity | 18 | transporter activity | Molecular Function |  |
| 3 | substrate-specific transporter activity | 18 | transporter activity | Molecular Function |  |
| 4 | passive transmembrane transporter activity | 18 | transmembrane transporter activity | Molecular Function |  |
| 2 | translation regulator activity | 1 | molecular_function | Molecular Function |  |
