# Supplementary material for: Development of Reference Transcriptomes for the Major Field Insect Pests of Cowpea: A Toolbox for Insect Pest Management Approaches in West Africa
Source: PLoS One. 2013 Nov 22;8(11):e79929. doi: 10.1371/journal.pone.0079929 (PMC3838393; doi:10.1371/journal.pone.0079929)
Supplement: Table S2 — InterproScan results from A. craccivora contigs that showed BLASTn hits to B. aphidicola. (DOCX) [file pone.0079929.s003.docx]

**Table S2.**

| **Contig ID** | **InterProScan** | | | |
| --- | --- | --- | --- | --- |
| Aphis 47 | noIPR | unintegrated | unintegrated | SignalP-NN(euk) (SIGNALP) |
| Aphis 47 | noIPR | unintegrated | unintegrated | tmhmm (TMHMM) |
| Aphis 47 | noIPR | unintegrated | unintegrated |  |
| Aphis 65 | IPR025472 | Protein of unknown function DUF4323 | Family | PF14211 (PFAM) |
| Aphis 65 | IPR025472 | Protein of unknown function DUF4323 | Family |  |
| Aphis 221 | IPR000850 | Adenylate kinase | Family | PR00094 (PRINTS) |
| Aphis 221 | IPR000850 | Adenylate kinase | Family | PTHR23359 (PANTHER) |
| Aphis 221 | IPR000850 | Adenylate kinase | Family | PF00406 (PFAM) |
| Aphis 221 | IPR000850 | Adenylate kinase | Family | GO:0005524 |
| Aphis 221 | IPR000850 | Adenylate kinase | Family | GO:0006139 |
| Aphis 221 | IPR000850 | Adenylate kinase | Family | GO:0019205 |
| Aphis 221 | IPR027417 | P-loop containing nucleoside triphosphate hydrolase | Domain | SSF52540 (SUPERFAMILY) |
| Aphis 221 | IPR027417 | P-loop containing nucleoside triphosphate hydrolase | Domain |  |
| Aphis 221 | noIPR | unintegrated | unintegrated | G3DSA:3.40.50.300 (GENE3D) |
| Aphis 221 | noIPR | unintegrated | unintegrated |  |
| Aphis 367 | noIPR | unintegrated | unintegrated | SignalP-NN(euk) (SIGNALP) |
| Aphis 367 | noIPR | unintegrated | unintegrated |  |
| Aphis 1561 | IPR001347 | Sugar isomerase (SIS) | Domain | PF01380 (PFAM) |
| Aphis 1561 | IPR001347 | Sugar isomerase (SIS) | Domain | PS51464 (PROFILE) |
| Aphis 1561 | IPR001347 | Sugar isomerase (SIS) | Domain | GO:0005975 |
| Aphis 1561 | IPR001347 | Sugar isomerase (SIS) | Domain | GO:0030246 |
| Aphis 1561 | IPR005855 | Glucosamine-fructose-6-phosphate aminotransferase, isomerising | Family | PTHR10937:SF0 (PANTHER) |
| Aphis 1561 | IPR005855 | Glucosamine-fructose-6-phosphate aminotransferase, isomerising | Family | GO:0004360 |
| Aphis 1561 | IPR005855 | Glucosamine-fructose-6-phosphate aminotransferase, isomerising | Family | GO:0005737 |
| Aphis 1561 | IPR005855 | Glucosamine-fructose-6-phosphate aminotransferase, isomerising | Family | GO:0016051 |
| Aphis 1561 | noIPR | unintegrated | unintegrated | G3DSA:3.40.50.10490 (GENE3D) |
| Aphis 1561 | noIPR | unintegrated | unintegrated | PTHR10937 (PANTHER) |
| Aphis 1561 | noIPR | unintegrated | unintegrated | SSF53697 (SUPERFAMILY) |
| Aphis 1561 | noIPR | unintegrated | unintegrated |  |
| Aphis 2076 | IPR002508 | Cell wall hydrolase/autolysin, catalytic | Domain | G3DSA:3.40.630.40 (GENE3D) |
| Aphis 2076 | IPR002508 | Cell wall hydrolase/autolysin, catalytic | Domain | PF01520 (PFAM) |
| Aphis 2076 | IPR002508 | Cell wall hydrolase/autolysin, catalytic | Domain | GO:0008745 |
| Aphis 2076 | IPR002508 | Cell wall hydrolase/autolysin, catalytic | Domain | GO:0009253 |
| Aphis 2076 | noIPR | unintegrated | unintegrated | PTHR30404 (PANTHER) |
| Aphis 2076 | noIPR | unintegrated | unintegrated | PTHR30404:SF0 (PANTHER) |
| Aphis 2076 | noIPR | unintegrated | unintegrated |  |
| Aphis 2391 | IPR001353 | Proteasome, subunit alpha/beta | Family | PF00227 (PFAM) |
| Aphis 2391 | IPR001353 | Proteasome, subunit alpha/beta | Family | GO:0004298 |
| Aphis 2391 | IPR001353 | Proteasome, subunit alpha/beta | Family | GO:0005839 |
| Aphis 2391 | IPR001353 | Proteasome, subunit alpha/beta | Family | GO:0051603 |
| Aphis 2391 | noIPR | unintegrated | unintegrated | G3DSA:3.60.20.10 (GENE3D) |
| Aphis 2391 | noIPR | unintegrated | unintegrated | PTHR32194 (PANTHER) |
| Aphis 2391 | noIPR | unintegrated | unintegrated | PTHR32194:SF0 (PANTHER) |
| Aphis 2391 | noIPR | unintegrated | unintegrated | SSF56235 (SUPERFAMILY) |
| Aphis 2391 | noIPR | unintegrated | unintegrated |  |
| Aphis 2472 | IPR004160 | Translation elongation factor EFTu/EF1A, C-terminal | Domain | PF03143 (PFAM) |
| Aphis 2472 | IPR004160 | Translation elongation factor EFTu/EF1A, C-terminal | Domain | GO:0005525 |
| Aphis 2472 | IPR004161 | Translation elongation factor EFTu/EF1A, domain 2 | Domain | PF03144 (PFAM) |
| Aphis 2472 | IPR004161 | Translation elongation factor EFTu/EF1A, domain 2 | Domain | GO:0005525 |
| Aphis 2472 | IPR004541 | Translation elongation factor EFTu/EF1A, bacterial/organelle | Family | PTHR23115:SF31 (PANTHER) |
| Aphis 2472 | IPR004541 | Translation elongation factor EFTu/EF1A, bacterial/organelle | Family | GO:0003746 |
| Aphis 2472 | IPR004541 | Translation elongation factor EFTu/EF1A, bacterial/organelle | Family | GO:0005525 |
| Aphis 2472 | IPR004541 | Translation elongation factor EFTu/EF1A, bacterial/organelle | Family | GO:0005622 |
| Aphis 2472 | IPR004541 | Translation elongation factor EFTu/EF1A, bacterial/organelle | Family | GO:0006414 |
| Aphis 2472 | IPR009000 | Translation elongation/initiation factor/Ribosomal, beta-barrel | Domain | SSF50447 (SUPERFAMILY) |
| Aphis 2472 | IPR009000 | Translation elongation/initiation factor/Ribosomal, beta-barrel | Domain |  |
| Aphis 2472 | IPR009001 | Translation elongation factor EF1A/initiation factor IF2gamma, C-terminal | Domain | SSF50465 (SUPERFAMILY) |
| Aphis 2472 | IPR009001 | Translation elongation factor EF1A/initiation factor IF2gamma, C-terminal | Domain |  |
| Aphis 2472 | noIPR | unintegrated | unintegrated | G3DSA:2.40.30.10 (GENE3D) |
| Aphis 2472 | noIPR | unintegrated | unintegrated | PTHR23115 (PANTHER) |
| Aphis 2472 | noIPR | unintegrated | unintegrated |  |
| Aphis 2530 | IPR000529 | Ribosomal protein S6 | Family | PF01250 (PFAM) |
| Aphis 2530 | IPR000529 | Ribosomal protein S6 | Family | TIGR00166 (TIGRFAMs) |
| Aphis 2530 | IPR000529 | Ribosomal protein S6 | Family | SSF54995 (SUPERFAMILY) |
| Aphis 2530 | IPR000529 | Ribosomal protein S6 | Family | GO:0003735 |
| Aphis 2530 | IPR000529 | Ribosomal protein S6 | Family | GO:0005840 |
| Aphis 2530 | IPR000529 | Ribosomal protein S6 | Family | GO:0006412 |
| Aphis 2530 | IPR000529 | Ribosomal protein S6 | Family | GO:0019843 |
| Aphis 2530 | IPR014717 | Translation elongation factor EF1B/ribosomal protein S6 | Domain | G3DSA:3.30.70.60 (GENE3D) |
| Aphis 2530 | IPR014717 | Translation elongation factor EF1B/ribosomal protein S6 | Domain |  |
| Aphis 2530 | IPR020815 | Ribosomal protein S6, conserved site | Conserved_site | PS01048 (PROSITE) |
| Aphis 2530 | IPR020815 | Ribosomal protein S6, conserved site | Conserved_site | GO:0003735 |
| Aphis 2530 | IPR020815 | Ribosomal protein S6, conserved site | Conserved_site | GO:0005840 |
| Aphis 2530 | IPR020815 | Ribosomal protein S6, conserved site | Conserved_site | GO:0006412 |
| Aphis 2530 | IPR020815 | Ribosomal protein S6, conserved site | Conserved_site | GO:0019843 |
| Aphis 2736 | noIPR | unintegrated | unintegrated | SignalP-NN(euk) (SIGNALP) |
| Aphis 2736 | noIPR | unintegrated | unintegrated |  |
| Aphis 3831 | IPR006847 | Translation initiation factor IF-2, N-terminal | Domain | PF04760 (PFAM) |
| Aphis 3831 | IPR006847 | Translation initiation factor IF-2, N-terminal | Domain | GO:0003743 |
| Aphis 3831 | IPR006847 | Translation initiation factor IF-2, N-terminal | Domain | GO:0006413 |
| Aphis 3831 | IPR009061 | DNA binding domain, putative | Domain | SSF46955 (SUPERFAMILY) |
| Aphis 3831 | IPR009061 | DNA binding domain, putative | Domain | GO:0000166 |
| Aphis 3831 | IPR013575 | Initiation factor 2 associated domain, bacterial | Domain | PF08364 (PFAM) |
| Aphis 3831 | IPR013575 | Initiation factor 2 associated domain, bacterial | Domain |  |
| Aphis 3831 | noIPR | unintegrated | unintegrated | G3DSA:3.30.56.50 (GENE3D) |
| Aphis 3831 | noIPR | unintegrated | unintegrated |  |
| Aphis 3870 | IPR016484 | GTP-binding protein EngA | Family | PTHR11649:SF5 (PANTHER) |
| Aphis 3870 | IPR016484 | GTP-binding protein EngA | Family | GO:0005525 |
| Aphis 3870 | noIPR | unintegrated | unintegrated | G3DSA:3.40.50.300 (GENE3D) |
| Aphis 3870 | noIPR | unintegrated | unintegrated | PTHR11649 (PANTHER) |
| Aphis 3870 | noIPR | unintegrated | unintegrated |  |
| Aphis 4564 | IPR000795 | Elongation factor, GTP-binding domain | Domain | PF00009 (PFAM) |
| Aphis 4564 | IPR000795 | Elongation factor, GTP-binding domain | Domain | GO:0003924 |
| Aphis 4564 | IPR000795 | Elongation factor, GTP-binding domain | Domain | GO:0005525 |
| Aphis 4564 | IPR005225 | Small GTP-binding protein domain | Domain | TIGR00231 (TIGRFAMs) |
| Aphis 4564 | IPR005225 | Small GTP-binding protein domain | Domain | GO:0005525 |
| Aphis 4564 | IPR006847 | Translation initiation factor IF-2, N-terminal | Domain | PF04760 (PFAM) |
| Aphis 4564 | IPR006847 | Translation initiation factor IF-2, N-terminal | Domain | GO:0003743 |
| Aphis 4564 | IPR006847 | Translation initiation factor IF-2, N-terminal | Domain | GO:0006413 |
| Aphis 4564 | IPR015760 | Translation initiation factor IF- 2 | Family | PTHR23115:SF41 (PANTHER) |
| Aphis 4564 | IPR015760 | Translation initiation factor IF- 2 | Family |  |
| Aphis 4564 | IPR027417 | P-loop containing nucleoside triphosphate hydrolase | Domain | SSF52540 (SUPERFAMILY) |
| Aphis 4564 | IPR027417 | P-loop containing nucleoside triphosphate hydrolase | Domain |  |
| Aphis 4564 | noIPR | unintegrated | unintegrated | G3DSA:3.40.50.300 (GENE3D) |
| Aphis 4564 | noIPR | unintegrated | unintegrated | PTHR23115 (PANTHER) |
| Aphis 4564 | noIPR | unintegrated | unintegrated |  |
| Aphis 4568 | IPR000819 | Peptidase M17, leucyl aminopeptidase, C-terminal | Domain | PF00883 (PFAM) |
| Aphis 4568 | IPR000819 | Peptidase M17, leucyl aminopeptidase, C-terminal | Domain | GO:0004177 |
| Aphis 4568 | IPR000819 | Peptidase M17, leucyl aminopeptidase, C-terminal | Domain | GO:0005622 |
| Aphis 4568 | IPR000819 | Peptidase M17, leucyl aminopeptidase, C-terminal | Domain | GO:0006508 |
| Aphis 4568 | noIPR | unintegrated | unintegrated | G3DSA:3.40.630.10 (GENE3D) |
| Aphis 4568 | noIPR | unintegrated | unintegrated | PTHR11963 (PANTHER) |
| Aphis 4568 | noIPR | unintegrated | unintegrated | PTHR11963:SF4 (PANTHER) |
| Aphis 4568 | noIPR | unintegrated | unintegrated | SSF53187 (SUPERFAMILY) |
| Aphis 4568 | noIPR | unintegrated | unintegrated |  |
| Aphis 5021 | IPR027417 | P-loop containing nucleoside triphosphate hydrolase | Domain | SSF52540 (SUPERFAMILY) |
| Aphis 5021 | IPR027417 | P-loop containing nucleoside triphosphate hydrolase | Domain |  |
| Aphis 5021 | noIPR | unintegrated | unintegrated | G3DSA:3.40.50.300 (GENE3D) |
| Aphis 5021 | noIPR | unintegrated | unintegrated | PTHR19211 (PANTHER) |
| Aphis 5021 | noIPR | unintegrated | unintegrated | PTHR19211:SF7 (PANTHER) |
| Aphis 5021 | noIPR | unintegrated | unintegrated |  |
| Aphis 5225 | IPR002133 | S-adenosylmethionine synthetase | Family | PTHR11964 (PANTHER) |
| Aphis 5225 | IPR002133 | S-adenosylmethionine synthetase | Family | GO:0004478 |
| Aphis 5225 | IPR002133 | S-adenosylmethionine synthetase | Family | GO:0005524 |
| Aphis 5225 | IPR002133 | S-adenosylmethionine synthetase | Family | GO:0006556 |
| Aphis 5225 | IPR022628 | S-adenosylmethionine synthetase, N-terminal | Domain | PF00438 (PFAM) |
| Aphis 5225 | IPR022628 | S-adenosylmethionine synthetase, N-terminal | Domain | GO:0004478 |
| Aphis 5225 | IPR022628 | S-adenosylmethionine synthetase, N-terminal | Domain | GO:0006556 |
| Aphis 5225 | IPR022636 | S-adenosylmethionine synthetase superfamily | Domain | SSF55973 (SUPERFAMILY) |
| Aphis 5225 | IPR022636 | S-adenosylmethionine synthetase superfamily | Domain | GO:0004478 |
| Aphis 5225 | IPR022636 | S-adenosylmethionine synthetase superfamily | Domain | GO:0006556 |
| Aphis 5225 | noIPR | unintegrated | unintegrated | G3DSA:3.30.300.10 (GENE3D) |
| Aphis 5225 | noIPR | unintegrated | unintegrated | PTHR11964:SF0 (PANTHER) |
| Aphis 5225 | noIPR | unintegrated | unintegrated |  |
| Aphis 5524 | IPR008580 | Domain of unknown function DUF862, eukaryotic | Domain | PF05903 (PFAM) |
| Aphis 5524 | IPR008580 | Domain of unknown function DUF862, eukaryotic | Domain |  |
| Aphis 5524 | noIPR | unintegrated | unintegrated | PTHR12378 (PANTHER) |
| Aphis 5524 | noIPR | unintegrated | unintegrated |  |
| Aphis 5691 | IPR001451 | Bacterial transferase hexapeptide repeat | Repeat | PF00132 (PFAM) |
| Aphis 5691 | IPR001451 | Bacterial transferase hexapeptide repeat | Repeat |  |
| Aphis 5691 | IPR005882 | Bifunctional UDP-N-acetylglucosamine pyrophosphorylase/glucosamine-1-phosphate N-acetyltransferase | Family | PTHR22572:SF17 (PANTHER) |
| Aphis 5691 | IPR005882 | Bifunctional UDP-N-acetylglucosamine pyrophosphorylase/glucosamine-1-phosphate N-acetyltransferase | Family | GO:0000287 |
| Aphis 5691 | IPR005882 | Bifunctional UDP-N-acetylglucosamine pyrophosphorylase/glucosamine-1-phosphate N-acetyltransferase | Family | GO:0000902 |
| Aphis 5691 | IPR005882 | Bifunctional UDP-N-acetylglucosamine pyrophosphorylase/glucosamine-1-phosphate N-acetyltransferase | Family | GO:0003977 |
| Aphis 5691 | IPR005882 | Bifunctional UDP-N-acetylglucosamine pyrophosphorylase/glucosamine-1-phosphate N-acetyltransferase | Family | GO:0005737 |
| Aphis 5691 | IPR005882 | Bifunctional UDP-N-acetylglucosamine pyrophosphorylase/glucosamine-1-phosphate N-acetyltransferase | Family | GO:0009103 |
| Aphis 5691 | IPR005882 | Bifunctional UDP-N-acetylglucosamine pyrophosphorylase/glucosamine-1-phosphate N-acetyltransferase | Family | GO:0009252 |
| Aphis 5691 | IPR005882 | Bifunctional UDP-N-acetylglucosamine pyrophosphorylase/glucosamine-1-phosphate N-acetyltransferase | Family | GO:0019134 |
| Aphis 5691 | IPR011004 | Trimeric LpxA-like | Domain | SSF51161 (SUPERFAMILY) |
| Aphis 5691 | IPR011004 | Trimeric LpxA-like | Domain | GO:0016740 |
| Aphis 5691 | noIPR | unintegrated | unintegrated | G3DSA:2.160.10.10 (GENE3D) |
| Aphis 5691 | noIPR | unintegrated | unintegrated | PTHR22572 (PANTHER) |
| Aphis 5691 | noIPR | unintegrated | unintegrated |  |
| Aphis 5984 | IPR001844 | Chaperonin Cpn60 | Family | PR00298 (PRINTS) |
| Aphis 5984 | IPR001844 | Chaperonin Cpn60 | Family | GO:0005737 |
| Aphis 5984 | IPR001844 | Chaperonin Cpn60 | Family | GO:0042026 |
| Aphis 5984 | IPR002423 | Chaperonin Cpn60/TCP-1 | Family | PTHR11353 (PANTHER) |
| Aphis 5984 | IPR002423 | Chaperonin Cpn60/TCP-1 | Family | PF00118 (PFAM) |
| Aphis 5984 | IPR002423 | Chaperonin Cpn60/TCP-1 | Family | SSF48592 (SUPERFAMILY) |
| Aphis 5984 | IPR002423 | Chaperonin Cpn60/TCP-1 | Family | GO:0005524 |
| Aphis 5984 | IPR002423 | Chaperonin Cpn60/TCP-1 | Family | GO:0044267 |
| Aphis 5984 | IPR018370 | Chaperonin Cpn60, conserved site | Conserved_site | PS00296 (PROSITE) |
| Aphis 5984 | IPR018370 | Chaperonin Cpn60, conserved site | Conserved_site | GO:0005524 |
| Aphis 5984 | IPR018370 | Chaperonin Cpn60, conserved site | Conserved_site | GO:0005737 |
| Aphis 5984 | IPR018370 | Chaperonin Cpn60, conserved site | Conserved_site | GO:0006457 |
| Aphis 5984 | IPR027409 | GroEL-like apical domain | Domain | G3DSA:3.50.7.10 (GENE3D) |
| Aphis 5984 | IPR027409 | GroEL-like apical domain | Domain | SSF52029 (SUPERFAMILY) |
| Aphis 5984 | IPR027409 | GroEL-like apical domain | Domain |  |
| Aphis 5984 | IPR027413 | GroEL-like equatorial domain | Domain | G3DSA:1.10.560.10 (GENE3D) |
| Aphis 5984 | IPR027413 | GroEL-like equatorial domain | Domain |  |
| Aphis 5984 | noIPR | unintegrated | unintegrated | PTHR11353:SF10 (PANTHER) |
| Aphis 5984 | noIPR | unintegrated | unintegrated |  |
| Aphis 6159 | IPR011603 | 2-oxoglutarate dehydrogenase, E1 component | Family | PTHR23152 (PANTHER) |
| Aphis 6159 | IPR011603 | 2-oxoglutarate dehydrogenase, E1 component | Family | GO:0004591 |
| Aphis 6159 | IPR011603 | 2-oxoglutarate dehydrogenase, E1 component | Family | GO:0006099 |
| Aphis 6159 | IPR011603 | 2-oxoglutarate dehydrogenase, E1 component | Family | GO:0030976 |
| Aphis 6159 | IPR011603 | 2-oxoglutarate dehydrogenase, E1 component | Family | GO:0055114 |
| Aphis 6159 | noIPR | unintegrated | unintegrated | PTHR23152:SF0 (PANTHER) |
| Aphis 6159 | noIPR | unintegrated | unintegrated |  |
| Aphis 6899 | noIPR | unintegrated | unintegrated | SignalP-NN(euk) (SIGNALP) |
| Aphis 6899 | noIPR | unintegrated | unintegrated |  |
| Aphis 7020 | IPR002903 | Ribosomal RNA small subunit methyltransferase H | Family | PF01795 (PFAM) |
| Aphis 7020 | IPR002903 | Ribosomal RNA small subunit methyltransferase H | Family | GO:0008168 |
| Aphis 7209 | noIPR | unintegrated | unintegrated | G3DSA:3.40.1280.10 (GENE3D) |
| Aphis 7209 | noIPR | unintegrated | unintegrated |  |
| Aphis 7344 | IPR000454 | ATPase, F0 complex, subunit C | Family | PR00124 (PRINTS) |
| Aphis 7344 | IPR000454 | ATPase, F0 complex, subunit C | Family | G3DSA:1.20.20.10 (GENE3D) |
| Aphis 7344 | IPR000454 | ATPase, F0 complex, subunit C | Family | GO:0015078 |
| Aphis 7344 | IPR000454 | ATPase, F0 complex, subunit C | Family | GO:0015986 |
| Aphis 7344 | IPR002379 | V-ATPase proteolipid subunit C-like domain | Domain | PF00137 (PFAM) |
| Aphis 7344 | IPR002379 | V-ATPase proteolipid subunit C-like domain | Domain | SSF81333 (SUPERFAMILY) |
| Aphis 7344 | IPR002379 | V-ATPase proteolipid subunit C-like domain | Domain | GO:0015078 |
| Aphis 7344 | IPR002379 | V-ATPase proteolipid subunit C-like domain | Domain | GO:0015991 |
| Aphis 7344 | IPR005953 | ATPase, F0 complex, subunit C, bacterial/chloroplast | Family | TIGR01260 (TIGRFAMs) |
| Aphis 7344 | IPR005953 | ATPase, F0 complex, subunit C, bacterial/chloroplast | Family | GO:0015078 |
| Aphis 7344 | IPR005953 | ATPase, F0 complex, subunit C, bacterial/chloroplast | Family | GO:0015986 |
| Aphis 7344 | IPR020537 | ATPase, F0 complex, subunit C, DCCD-binding site | Binding_site | PS00605 (PROSITE) |
| Aphis 7344 | IPR020537 | ATPase, F0 complex, subunit C, DCCD-binding site | Binding_site |  |
| Aphis 7344 | noIPR | unintegrated | unintegrated | SignalP-NN(euk) (SIGNALP) |
| Aphis 7344 | noIPR | unintegrated | unintegrated | tmhmm (TMHMM) |
| Aphis 7344 | noIPR | unintegrated | unintegrated |  |
