# Supplementary material for: Development of Reference Transcriptomes for the Major Field Insect Pests of Cowpea: A Toolbox for Insect Pest Management Approaches in West Africa
Source: PLoS One. 2013 Nov 22;8(11):e79929. doi: 10.1371/journal.pone.0079929 (PMC3838393; doi:10.1371/journal.pone.0079929)
Supplement: Table S3 — a. Summary of all SNPs detected in contigs in A. curvipes, including sequence description, length, organism, minimum e-value, number of GOs and number of SNPs associated with each contig. b. Summary of all SNPs detected in contigs in A. craccivora, including sequence description, length, organism, minimum e-value, number of GOs and number of SNPs associated with each contig. c. Summary of all SNPs detected in contigs in C. tomentosicollis, including sequence description, length, organism, minimum e-value, number of GOs and number of SNPs associated with each contig. d. Summary of all SNPs detected in contigs in M. sjostedti, including sequence description, length, organism, minimum e-value, number of GOs and number of SNPs associated with each contig. (DOCX) [file pone.0079929.s004.docx]

**Table S3a.**

| **Contig ID** | **Sequence Description** | **Length (bp)** | **Organism** | **Minimum E-value** | **#GOs** | **#SNPs** |
| --- | --- | --- | --- | --- | --- | --- |
| Anop 7 | elongation factor 1-alpha | 2152 | *Riptortus pedestris* | 0 | 6 | 1 |
| Anop 13 | atp synthase subunit mitochondrial-like | 2165 | *Toxoptera citricida* | 0 | 6 | 1 |
| Anop 14 | ankyrin repeat protein | 1054 | *Candidatus Amoebophilus asiaticus* | 5.64E-58 | 6 | 4 |
| Anop 15 | ankyrin repeat protein | 874 | *Candidatus Amoebophilus asiaticus* | 4.05E-47 | 6 | 10 |
| Anop 17 | cg7630 cg7630-pa | 772 | *Triatoma brasiliensis* | 3.58E-18 | 0 | 2 |
| Anop 23 | rrna intron-encoded homing endonuclease | 2738 | *Oxytricha trifallax* | 1.17E-55 | 1 | 1 |
| Anop 25 | cg41536 cg41536- partial | 3200 | *Daphnia pulex* | 1.90E-57 | 0 | 7 |
| Anop 26 | trifunctional purine biosynthetic protein adenosine-3 | 1487 | *Tribolium castaneum* | 0 | 6 | 2 |
| Anop 27 | transferrin | 305 | *Riptortus clavatus* | 9.41E-08 | 4 | 4 |
| Anop 31 | transferrin | 1344 | *Riptortus clavatus* | 0 | 4 | 5 |
| Anop 33 | achain crystal structure of engineered northeast structural genomics consortium target | 639 | synthetic construct | 1.17E-31 | 4 | 3 |
| Anop 34 | orf16-lacz fusion protein | 1781 | *Heliobacterium modesticaldum Ice1* | 2.91E-53 | 3 | 2 |
| Anop 35 | N/A | 851 |  |  | 0 | 4 |
| Anop 50 | atp-citrate synthase | 3596 | *Acyrthosiphon pisum* | 0 | 6 | 12 |
| Anop 51 | atp synthase f0 subunit 6 | 321 | *Stictopleurus subviridis* | 1.47E-38 | 4 | 2 |
| Anop 52 | atp synthase f0 subunit 6 | 315 | *Stictopleurus subviridis* | 5.60E-37 | 4 | 1 |
| Anop 53 | atpase subunit 6 | 310 | *Riptortus pedestris* | 2.66E-22 | 4 | 4 |
| Anop 54 | atp synthase f0 subunit 6 | 393 | *Stictopleurus subviridis* | 5.99E-47 | 4 | 2 |
| Anop 55 | pleiotrophin-like protein | 1400 | *Tribolium castaneum* | 4.47E-40 | 2 | 1 |
| Anop 58 | basic juvenile hormone sensitive hemolymph protein | 714 | *Riptortus clavatus* | 2.30E-119 | 6 | 1 |
| Anop 64 | midline fasciclin | 3028 | *Tribolium castaneum* | 5.98E-81 | 3 | 6 |
| Anop 71 | arylphorin receptor | 4026 | *Calliphora vicina* | 0 | 3 | 11 |
| Anop 72 | N/A | 355 |  |  | 0 | 3 |
| Anop 84 | apolipophorins | 4036 | *Apis mellifera* | 3.14E-102 | 1 | 3 |
| Anop 87 | adp atp translocase | 1573 | *Triatoma infestans* | 0 | 4 | 1 |
| Anop 92 | 15-hydroxyprostaglandin dehydrogenase | 1077 | *Acromyrmex echinatior* | 3.00E-43 | 4 | 2 |
| Anop 97 | ribosomal protein s18 | 571 | *Cicindela campestris* | 2.94E-86 | 4 | 1 |
| Anop 99 | hemi_pyrap ame: full=hemiptericin | 603 | *Hemiptericin* | 5.33E-23 | 3 | 1 |
| Anop 106 | cytochrome b | 463 | *Aeschyntelus notatus* | 3.73E-61 | 7 | 4 |
| Anop 107 | cytochrome b | 849 | *Hydaropsis longirostris* | 3.70E-129 | 7 | 5 |
| Anop 112 | enolase | 3401 | *Dendroctonus ponderosae* | 0 | 4 | 8 |
| Anop 113 | atp synthase subunit mitochondrial | 1231 | *Apis mellifera* | 0 | 6 | 2 |
| Anop 120 | glyceraldehyde-3-phosphate dehydrogenase | 400 | *Maconellicoccus hirsutus* | 4.12E-31 | 7 | 1 |
| Anop 121 | glyceraldhyde-3-phosphate partial | 389 | *Apolygus lucorum* | 1.51E-64 | 3 | 1 |
| Anop 123 | glyceraldehyde 3 phosphate dehydrogenase 1 | 535 | *Apolygus lucorum* | 1.06E-97 | 8 | 3 |
| Anop 125 | cytochrome c oxidase subunit partial | 519 | *Aeschyntelus notatus* | 1.65E-86 | 8 | 2 |
| Anop 133 | apolipophorin-iii precursor | 580 | *Riptortus clavatus* | 1.13E-62 | 3 | 1 |
| Anop 152 | tropomyosin 1 | 1814 | *Megachile rotundata* | 2.51E-158 | 3 | 2 |
| Anop 153 | myosin heavy muscle isoform 1 | 6145 | *Acyrthosiphon pisum* | 0 | 42 | 1 |
| Anop 159 | N/A | 358 |  |  | 0 | 1 |
| Anop 161 | trifunctional enzyme beta subunit (tp-beta) | 832 | *Tribolium castaneum* | 3.07E-135 | 7 | 1 |
| Anop 162 | vitellogenin | 4518 | *Riptortus clavatus* | 0 | 2 | 6 |
| Anop 163 | cytochrome c oxidase subunit iii | 1109 | *Stictopleurus subviridis* | 5.33E-55 | 4 | 1 |
| Anop 164 | cytochrome c oxidase subunit iii | 367 | *Stictopleurus subviridis* | 1.94E-62 | 4 | 1 |
| Anop 165 | proactivator polypeptide | 2113 | *Tribolium castaneum* | 1.32E-137 | 4 | 1 |
| Anop 169 | lipoyltransferase mitochondrial-like | 3307 | *Bombus terrestris* | 4.31E-122 | 2 | 7 |
| Anop 175 | fatty acid synthase | 7421 | *Nasonia vitripennis* | 0 | 6 | 4 |
| Anop 184 | nadh dehydrogenase subunit 5 | 604 | *Stictopleurus subviridis* | 6.57E-35 | 4 | 2 |
| Anop 187 | hexamerin 1 | 784 | *Riptortus clavatus* | 8.69E-113 | 6 | 10 |
| Anop 188 | vitellogenin | 2422 | *Riptortus clavatus* | 7.45E-168 | 4 | 3 |
| Anop 198 | ribosomal protein l9 | 709 | *Tribolium castaneum* | 5.21E-119 | 4 | 1 |
| Anop 205 | actin | 1506 | *Drosophila melanogaster* | 0 | 13 | 1 |
| Anop 213 | ribosomal protein s25 | 413 | *Diaphorina citri* | 1.57E-40 | 3 | 1 |
| Anop 214 | probable bax inhibitor 1-like | 1533 | *Triatoma infestans* | 2.98E-111 | 1 | 1 |
| Anop 215 | N/A | 295 |  |  | 0 | 7 |
| Anop 236 | heat shock protein 70 | 776 | *Pyrrhocoris apterus* | 6.14E-154 | 2 | 2 |
| Anop 238 | N/A | 382 |  |  | 0 | 2 |
| Anop 241 | mitochondrial porin | 1644 | *Homalodisca vitripennis* | 4.96E-133 | 11 | 1 |
| Anop 249 | cathepsin l-like | 438 | *Triatoma brasiliensis* | 5.67E-63 | 1 | 1 |
| Anop 258 | ribosomal protein s9 | 690 | *Meladema coriacea* | 7.39E-119 | 5 | 2 |
| Anop 261 | heat shock protein 70 | 571 | *Pyrrhocoris apterus* | 9.12E-96 | 4 | 3 |
| Anop 266 | heat shock protein 90 | 1073 | *Camponotus floridanus* | 0 | 4 | 3 |
| Anop 274 | serine threonine-protein phosphatase 6 regulatory ankyrin repeat subunit a-like | 290 | *Strongylocentrotus purpuratus* | 1.32E-15 | 2 | 1 |
| Anop 275 | imaginal disc growth factor | 901 | *Oncometopia nigricans* | 3.47E-105 | 2 | 7 |
| Anop 277 | imaginal disc growth factor | 419 | *Pieris rapae* | 3.05E-34 | 4 | 1 |
| Anop 282 | cytochrome oxidase subunit 1 | 610 | *Sethenira ferruginea* | 1.40E-74 | 10 | 3 |
| Anop 285 | N/A | 434 |  |  | 0 | 2 |
| Anop 290 | N/A | 424 |  |  | 0 | 3 |
| Anop 291 | nadh dehydrogenase subunit 2 | 455 | *Chauliops fallax* | 3.37E-24 | 5 | 5 |
| Anop 293 | nadh dehydrogenase subunit 2 | 553 | *Aeschyntelus notatus* | 9.67E-33 | 5 | 2 |
| Anop 296 | N/A | 673 |  |  | 0 | 1 |
| Anop 300 | cytochrome oxidase subunit 1 | 356 | *Homoeocerus sp. ST-2009* | 5.22E-28 | 10 | 1 |
| Anop 304 | probable atp-dependent rna helicase ddx17-like | 2214 | *Tribolium castaneum* | 0 | 6 | 2 |
| Anop 316 | ribosomal protein p1 | 526 | *Triatoma infestans* | 7.86E-33 | 3 | 1 |
| Anop 318 | N/A | 508 |  |  | 0 | 2 |
| Anop 324 | N/A | 563 |  |  | 0 | 2 |
| Anop 325 | vitellogenin | 2012 | *Lethocerus deyrollei* | 1.28E-124 | 2 | 3 |
| Anop 332 | cytochrome p450 | 901 | *Tribolium castaneum* | 3.01E-34 | 2 | 2 |
| Anop 334 | cytochrome oxidase subunit i | 219 | *Cletus punctiger* | 4.87E-35 | 10 | 3 |
| Anop 337 | ribosomal protein l23a | 1033 | *Tribolium castaneum* | 8.98E-79 | 7 | 1 |
| Anop 344 | nadh dehydrogenase subunit 2 | 360 | *Aeschyntelus notatus* | 3.83E-13 | 4 | 2 |
| Anop 348 | apolipophorin-iii precursor | 477 | *Riptortus clavatus* | 3.45E-59 | 3 | 1 |
| Anop 349 | apolipophorin-iii precursor | 472 | *Riptortus clavatus* | 5.47E-63 | 3 | 1 |
| Anop 351 | ankyrin repeat domain protein | 951 | *Strongylocentrotus purpuratus* | 8.78E-35 | 3 | 16 |
| Anop 354 | hypothetical protein EAI_16042 | 681 | *Harpegnathos saltator* | 9.95E-07 | 0 | 2 |
| Anop 358 | nadh dehydrogenase subunit i | 1020 | *Aeschyntelus notatus* | 1.09E-108 | 4 | 6 |
| Anop 364 | cytochrome b | 848 | *Riptortus pedestris* | 8.85E-42 | 7 | 6 |
| Anop 365 | disulfide isomerase | 1998 | *Litopenaeus vannamei* | 0 | 12 | 1 |
| Anop 376 | abp2_ripcl ame: full=probable antibacterial peptide flags: precursor | 945 | *Riptortus clavatus* | 2.01E-44 | 1 | 6 |
| Anop 377 | superoxide dismutase | 778 | *Triatoma infestans* | 2.65E-82 | 4 | 2 |
| Anop 380 | ubiquitin | 228 | *Cherax quadricarinatus* | 1.16E-46 | 61 | 5 |
| Anop 389 | N/A | 518 |  |  | 4 | 2 |
| Anop 392 | atp synthase delta mitochondrial | 566 | *Culex quinquefasciatus* | 1.11E-53 | 4 | 3 |
| Anop 395 | 40s ribosomal protein s2 | 1113 | *Tribolium castaneum* | 4.75E-139 | 4 | 3 |
| Anop 422 | N/A | 342 |  |  | 0 | 7 |
| Anop 433 | ribosomal protein l7a | 943 | *Solenopsis invicta* | 2.72E-97 | 2 | 2 |
| Anop 447 | ornithine decarboxylase | 1221 | *Pediculus humanus corporis* | 1.12E-42 | 2 | 1 |
| Anop 467 | mitochondrial phosphate carrier protein | 1338 | *Aedes aegypti* | 7.12E-164 | 4 | 4 |
| Anop 471 | cytochrome oxidase subunit partial | 361 | *Anoplocnemis phasianus* | 1.43E-51 | 10 | 1 |
| Anop 473 | cytochrome oxidase subunit 1 | 330 | *Leptocorisa vericornis* | 7.21E-55 | 10 | 1 |
| Anop 480 | ribonuclease uk114-like isoform 1 | 816 | *Drosophila virilis* | 3.86E-58 | 2 | 1 |
| Anop 485 | ankyrin repeat protein | 524 | *synthetic construct* | 9.81E-25 | 1 | 3 |
| Anop 494 | N/A | 662 |  |  | 0 | 6 |
| Anop 503 | AGAP010360-PA | 817 | *Anopheles gambiae str. PEST* | 4.83E-11 | 3 | 5 |
| Anop 517 | N/A | 244 |  |  | 0 | 1 |
| Anop 520 | ribosomal protein l24 | 544 | *Bombyx mori* | 9.31E-61 | 2 | 1 |
| Anop 523 | ribosomal protein l14 | 544 | *Lygus lineolaris* | 1.26E-68 | 3 | 1 |
| Anop 527 | luciferin-regenerating enzyme | 1169 | *Nasonia vitripennis* | 4.87E-70 | 3 | 1 |
| Anop 534 | N/A | 602 |  |  | 0 | 3 |
| Anop 541 | isoform cra_b | 403 | *Oncopeltus fasciatus* | 8.92E-20 | 7 | 1 |
| Anop 552 | acyl- -binding protein | 702 | *Rhodnius prolixus* | 3.61E-39 | 2 | 2 |
| Anop 561 | i-type lysozyme | 512 | *Nilaparvata lugens* | 3.77E-41 | 3 | 1 |
| Anop 568 | peroxiredoxin 1 | 1112 | *Coptotermes formosanus* | 8.53E-79 | 10 | 2 |
| Anop 569 | elongation factor 1 delta | 753 | *Graphocephala atropunctata* | 6.56E-65 | 9 | 1 |
| Anop 576 | alpha-glucosidase | 1276 | *Aedes aegypti* | 3.27E-90 | 4 | 13 |
| Anop 582 | cg31997 cg31997-pa | 616 | *Megachile rotundata* | 2.14E-43 | 3 | 1 |
| Anop 597 | polyadenylate-binding protein 1-like isoform 1 | 2456 | *Bombus terrestris* | 0 | 4 | 2 |
| Anop 602 | luciferin-regenerating enzyme | 843 | *Nasonia vitripennis* | 2.46E-45 | 3 | 12 |
| Anop 609 | cathepsin l | 523 | *Drosophila mojavensis* | 1.20E-61 | 5 | 2 |
| Anop 615 | cg12324 protein | 466 | *Triatoma infestans* | 8.81E-86 | 5 | 1 |
| Anop 622 | pheromone-degrading enzyme | 734 | *Pyrrhocoris apterus* | 4.12E-50 | 1 | 8 |
| Anop 643 | phosphatidylethanolamine-binding protein | 325 | *Apis mellifera* | 9.09E-11 | 0 | 5 |
| Anop 648 | N/A | 733 |  |  | 0 | 3 |
| Anop 658 | N/A | 374 |  |  | 0 | 5 |
| Anop 662 | ferritin heavy chain | 1009 | *Nasonia vitripennis* | 2.92E-56 | 6 | 1 |
| Anop 664 | salivary secreted cystatin 3 precursor | 574 | *Oncopeltus fasciatus* | 6.72E-16 | 5 | 3 |
| Anop 674 | odorant-binding protein | 732 | *Apolygus lucorum* | 3.77E-17 | 1 | 1 |
| Anop 678 | superoxide dismutase | 1406 | Cu-Zn | 8.83E-50 | 18 | 1 |
| Anop 683 | N/A | 350 |  |  | 0 | 8 |
| Anop 708 | N/A | 1002 |  |  | 0 | 6 |
| Anop 714 | N/A | 686 |  |  | 0 | 2 |
| Anop 729 | ubiquinol-cytochrome c reductase complex 14 kda protein | 709 | *Papilio xuthus* | 5.40E-43 | 6 | 3 |
| Anop 744 | mitochondrial cytochrome c oxidase subunit 5b isoform 1 | 562 | *Triatoma infestans* | 8.47E-51 | 4 | 2 |
| Anop 758 | N/A | 451 |  |  | 0 | 3 |
| Anop 759 | cytochrome c | 651 | *Graphocephala atropunctata* | 1.71E-62 | 9 | 3 |
| Anop 771 | N/A | 830 |  |  | 0 | 6 |
| Anop 772 | fructose -bisphosphate aldolase | 1414 | *Daphnia pulex* | 0 | 21 | 4 |
| Anop 782 | PREDICTED: hypothetical protein LOC100648520 | 386 | *Bombus terrestris* | 7.11E-07 | 0 | 6 |
| Anop 799 | cytochrome c oxidase polypeptide iv | 673 | *Locusta migratoria* | 4.36E-71 | 7 | 1 |
| Anop 800 | ribosomal protein l10 | 771 | *Acyrthosiphon pisum* | 1.12E-147 | 3 | 1 |
| Anop 805 | N/A | 402 |  |  | 0 | 6 |
| Anop 807 | 40s ribosomal protein s8-like | 709 | *Triatoma infestans* | 1.59E-123 | 3 | 1 |
| Anop 809 | ribosomal protein l31 | 696 | *Harpegnathos saltator* | 1.71E-62 | 3 | 2 |
| Anop 818 | glutamine synthetase 2 | 867 | *Nilaparvata lugens* | 1.07E-173 | 7 | 5 |
| Anop 825 | transferrin | 1095 | *Riptortus clavatus* | 4.20E-136 | 4 | 2 |
| Anop 828 | 60s ribosomal protein l5 | 972 | *Laodelphax striatella* | 4.66E-168 | 7 | 1 |
| Anop 829 | N/A | 644 |  |  | 0 | 1 |
| Anop 843 | fk506-binding protein | 638 | *Daphnia pulex* | 1.31E-63 | 6 | 2 |
| Anop 849 | 40s ribosomal protein s11 | 667 | *Maconellicoccus hirsutus* | 3.68E-74 | 3 | 1 |
| Anop 850 | peripheral-type benzodiazepine receptor | 1056 | *Drosophila grimshawi* | 3.22E-40 | 3 | 1 |
| Anop 857 | N/A | 731 |  |  | 0 | 1 |
| Anop 877 | suppressor of g2 allele of skp1 homolog | 1438 | *Gorilla gorilla gorilla* | 8.00E-47 | 6 | 2 |
| Anop 878 | inter-alpha-trypsin inhibitor heavy chain h4 precursor | 2250 | *Acyrthosiphon pisum* | 9.23E-130 | 2 | 7 |
| Anop 886 | pancreatic triacylglycerol lipase | 1779 | *Acyrthosiphon pisum* | 0 | 1 | 4 |
| Anop 902 | cathepsin b-like proteinase | 1396 | *Daphnia pulex* | 1.62E-157 | 3 | 1 |
| Anop 906 | heat shock protein 70 | 2713 | *Lycorma delicatula* | 0 | 5 | 8 |
| Anop 908 | guanine nucleotide-binding protein subunit beta-like | 1042 | *Blattella germanica* | 0 | 2 | 3 |
| Anop 911 | nadh dehydrogenase subunit 4 | 2548 | *Riptortus pedestris* | 2.50E-119 | 5 | 9 |
| Anop 958 | arginine kinase | 1742 | *Anasa tristis* | 0 | 3 | 2 |
| Anop 967 | N/A | 323 |  |  | 0 | 4 |
| Anop 971 | N/A | 818 |  |  | 0 | 3 |
| Anop 982 | N/A | 558 |  |  | 0 | 5 |
| Anop 1000 | thiamin pyrophosphokinase 1 | 1131 | *Tribolium castaneum* | 1.82E-55 | 1 | 1 |
| Anop 1017 | gelsolin precursor | 2022 | *Culex quinquefasciatus* | 1.14E-175 | 2 | 5 |
| Anop 1027 | N/A | 636 |  |  | 0 | 8 |
| Anop 1050 | phosphoenolpyruvate isoform a | 2429 | *Tribolium castaneum* | 0 | 4 | 8 |
| Anop 1084 | pneumolysin | 567 | *Streptococcus mitis SK597* | 1.69E-19 | 10 | 1 |
| Anop 1085 | serine rich protein | 2575 | *Nematostella vectensis* | 5.49E-54 | 5 | 2 |
| Anop 1109 | unknown | 1096 | *Lygus lineolaris* | 3.06E-10 | 0 | 1 |
| Anop 1125 | eukaryotic translation initiation factor x-chromosomal-like | 999 | *Triatoma infestans* | 2.40E-78 | 7 | 1 |
| Anop 1126 | arylphorin subunit a4 | 708 | *Calliphora vicina* | 1.25E-140 | 2 | 4 |
| Anop 1136 | N/A | 558 |  |  | 0 | 1 |
| Anop 1163 | sugar phosphate exchanger 2-like isoform 2 | 2076 | *Megachile rotundata* | 3.04E-179 | 3 | 1 |
| Anop 1166 | N/A | 407 |  |  | 0 | 2 |
| Anop 1170 | cathepsin d | 1027 | *Callosobruchus maculatus* | 8.02E-97 | 1 | 2 |
| Anop 1173 | N/A | 363 |  |  | 0 | 4 |
| Anop 1189 | vitellogenin | 624 | *Riptortus clavatus* | 5.41E-65 | 2 | 2 |
| Anop 1193 | phosphoserine aminotransferase | 1277 | *Pediculus humanus corporis* | 8.67E-168 | 4 | 2 |
| Anop 1201 | apolipoprotein d-like | 919 | *Nasonia vitripennis* | 1.28E-95 | 1 | 4 |
| Anop 1202 | chemosensory protein 1 | 414 | *Apolygus lucorum* | 2.14E-40 | 0 | 2 |
| Anop 1207 | cathepsin b | 1027 | *Branchiostoma floridae* | 3.92E-107 | 3 | 3 |
| Anop 1217 | ribosomal protein l13 | 787 | *Xenopsylla cheopis* | 8.21E-86 | 3 | 2 |
| Anop 1229 | cytosolic malate dehydrogenase | 1484 | *Pediculus humanus corporis* | 2.23E-164 | 8 | 2 |
| Anop 1233 | citrate synthase | 1848 | *Aedes aegypti* | 0 | 5 | 2 |
| Anop 1236 | ribosomal protein s15e | 445 | *Diaphorina citri* | 1.33E-66 | 6 | 1 |
| Anop 1237 | N/A | 650 |  |  | 0 | 3 |
| Anop 1243 | N/A | 703 |  |  | 0 | 3 |
| Anop 1268 | translationally controlled tumor protein | 866 | *Graphocephala atropunctata* | 2.33E-103 | 1 | 1 |
| Anop 1269 | atp synthase-like protein | 1078 | *Papilio xuthus* | 2.13E-86 | 8 | 1 |
| Anop 1276 | N/A | 1450 |  |  | 0 | 4 |
| Anop 1277 | N/A | 1292 |  |  | 1 | 1 |
| Anop 1294 | ribosomal protein l28 | 506 | *Triatoma brasiliensis* | 2.64E-74 | 3 | 2 |
| Anop 1295 | ankyrin repeat protein | 954 | *Synechococcus sp. JA-3-3Ab* | 7.39E-25 | 0 | 7 |
| Anop 1302 | cg31997 cg31997-pa | 540 | *Acyrthosiphon pisum* | 7.78E-47 | 1 | 2 |
| Anop 1312 | N/A | 727 |  |  | 0 | 4 |
| Anop 1349 | maltase a3 | 1179 | *Nilaparvata lugens* | 8.19E-96 | 1 | 2 |
| Anop 1357 | N/A | 843 |  |  | 0 | 1 |
| Anop 1361 | N/A | 938 |  |  | 0 | 3 |
| Anop 1375 | N/A | 1357 |  |  | 0 | 1 |
| Anop 1384 | N/A | 493 |  |  | 0 | 2 |
| Anop 1411 | vitellogenin | 1922 | *Riptortus clavatus* | 0 | 2 | 1 |
| Anop 1426 | nadh dehydrogenase subunit 5 | 295 | *Hydaropsis longirostris* | 1.02E-49 | 5 | 1 |
| Anop 1518 | nadh dehydrogenase subunit 5 | 962 | *Hydaropsis longirostris* | 6.06E-76 | 5 | 1 |
| Anop 1524 | elongation factor 1 beta | 772 | *Triatoma infestans* | 1.17E-108 | 3 | 1 |
| Anop 1526 | N/A | 450 |  |  | 0 | 5 |
| Anop 1550 | N/A | 441 |  |  | 0 | 1 |
| Anop 1553 | N/A | 939 |  |  | 0 | 2 |
| Anop 1563 | ribosomal protein l3 | 1276 | *Bombus terrestris* | 0 | 3 | 1 |
| Anop 1627 | small heat shock protein | 823 | *Maconellicoccus hirsutus* | 2.50E-46 | 1 | 6 |
| Anop 1666 | chemosensory protein 1 | 558 | *Apolygus lucorum* | 3.24E-51 | 0 | 1 |
| Anop 1686 | odorant binding protein 2 | 843 | *Apolygus lucorum* | 6.23E-11 | 0 | 4 |
| Anop 1692 | cellular retinaldehyde-binding protein | 1260 | *Tribolium castaneum* | 4.14E-120 | 3 | 3 |
| Anop 1703 | N/A | 467 |  |  | 0 | 3 |
| Anop 1766 | phosphoglycerate mutase | 991 | *Acyrthosiphon pisum* | 1.66E-140 | 2 | 1 |
| Anop 1776 | cytochrome p450 4g15 | 1857 | *Acromyrmex echinatior* | 0 | 6 | 1 |
| Anop 1794 | atp synthase-coupling factor mitochondrial | 809 | *Aedes albopictus* | 2.43E-33 | 4 | 1 |
| Anop 1815 | pyrimidine-specific ribonucleoside hydrolase riha-like | 1189 | *Bombus impatiens* | 4.91E-65 | 1 | 2 |
| Anop 1823 | lyzozyme m1 | 844 | Wolbachia endosymbiont of *Drosophila simulans* wNo | 5.29E-74 | 2 | 2 |
| Anop 1835 | acyl-protein thioesterase | 1828 | *Camponotus floridanus* | 2.65E-96 | 3 | 2 |
| Anop 1842 | 40s ribosomal protein s3a | 879 | *Triatoma infestans* | 4.32E-164 | 3 | 1 |
| Anop 1844 | serine proteinase stubble | 809 | *Megachile rotundata* | 1.69E-43 | 2 | 5 |
| Anop 1855 | chkov1 | 468 | *Drosophila persimilis* | 1.80E-12 | 4 | 1 |
| Anop 1856 | N/A | 849 |  |  | 0 | 7 |
| Anop 1896 | aminopeptidase -like | 1463 | *Apis mellifera* | 2.74E-177 | 2 | 3 |
| Anop 1909 | N/A | 430 |  |  | 0 | 2 |
| Anop 1945 | heat shock protein 60 | 2658 | *Apis mellifera* | 0 | 13 | 1 |
| Anop 1989 | catalase | 1763 | *Schistocerca gregaria* | 0 | 0 | 1 |
| Anop 2003 | heat shock protein | 963 | *Schistocerca gregaria* | 1.63E-53 | 2 | 1 |
| Anop 2142 | ribosomal protein l7 | 912 | *Papilio polytes* | 1.52E-103 | 2 | 1 |
| Anop 2149 | heat shock protein 90 | 419 | *Apis mellifera* | 1.70E-70 | 4 | 1 |
| Anop 2178 | thiol-activated cytolysin | 1072 | *Streptococcus mitis* | 2.69E-12 | 1 | 3 |
| Anop 2212 | glutathione s-transferase | 417 | *Aphis gossypii* | 5.51E-18 | 1 | 1 |
| Anop 2312 | hemi_pyrap ame: full=hemiptericin | 395 | *Pyrrhocoris apterus* | 8.00E-24 | 3 | 2 |
| Anop 2378 | brain protein 44-like | 450 | *Ixodes scapularis* | 1.30E-34 | 3 | 1 |
| Anop 2385 | N/A | 302 |  |  | 0 | 5 |
| Anop 2440 | ribosomal protein l11 | 679 | *Triatoma infestans* | 6.41E-120 | 7 | 2 |
| Anop 2477 | puromycin-sensitive aminopeptidase | 2848 | *Acromyrmex echinatior* | 1.90E-68 | 0 | 1 |
| Anop 2534 | cytochrome p450 | 1594 | *Culex quinquefasciatus* | 2.25E-45 | 8 | 1 |
| Anop 2559 | cytochrome b-c1 complex subunit mitochondrial-like | 1535 | *Tribolium castaneum* | 5.04E-67 | 6 | 1 |
| Anop 2571 | AGAP001981-PB | 818 | *Anopheles gambiae str. PEST* | 1.85E-09 | 0 | 3 |
| Anop 2593 | N/A | 513 |  |  | 0 | 1 |
| Anop 2635 | cathepsin l | 868 | *Litopenaeus vannamei* | 3.20E-87 | 1 | 2 |
| Anop 2636 | fatty acid binding protein | 529 | *Lygus lineolaris* | 1.02E-59 | 3 | 3 |
| Anop 2804 | N/A | 468 |  |  | 0 | 1 |
| Anop 2828 | N/A | 673 |  |  | 0 | 2 |
| Anop 2833 | GJ21142 | 344 | *Drosophila virilis* | 4.70E-23 | 0 | 9 |
| Anop 2861 | dihydrolipoamide dehydrogenase e3 subunit | 1732 | *Pediculus humanus corporis* | 0 | 5 | 1 |
| Anop 2926 | kda midgut protein | 332 | *Lygus lineolaris* | 6.26E-11 | 0 | 2 |
| Anop 3353 | probable maltase l-like | 376 | *Nilaparvata lugens* | 6.25E-18 | 1 | 15 |
| Anop 3481 | small heat shock protein | 946 | *Lygus hesperus* | 2.82E-65 | 2 | 3 |
| Anop 3688 | N/A | 321 |  |  | 0 | 6 |
| Anop 3823 | 3-hydroxyacyl-coa dehyrogenase | 1100 | *Papilio xuthus* | 6.73E-134 | 4 | 2 |
| Anop 4243 | N/A | 339 |  |  | 0 | 1 |
| Anop 5253 | N/A | 411 |  |  | 0 | 3 |
| Anop 6789 | N/A | 659 |  |  | 0 | 3 |
| Anop 8555 | N/A | 534 |  |  | 0 | 1 |

**Table S3b.**

| **Contig ID** | **Sequence Description** | **Length (bp)** | **Organism** | **Minimum E-value** | **#GOs** | **#SNPs** |
| --- | --- | --- | --- | --- | --- | --- |
| Aphis 41 | adp atp translocase | 1789 | *Acyrthosiphon pisum* | 0 | 4 | 2 |
| Aphis 45 | enolase | 1701 | *Acyrthosiphon pisum* | 0 | 5 | 1 |
| Aphis 85 | mitochondrial atp synthase f chain | 877 | *Acyrthosiphon pisum* | 1.73E-66 | 5 | 2 |
| Aphis 90 | 60s acidic ribosomal protein p2 | 454 | *Acyrthosiphon pisum* | 1.16E-33 | 3 | 3 |
| Aphis 102 | 60s ribosomal protein l11-like | 682 | *Acyrthosiphon pisum* | 1.86E-133 | 7 | 6 |
| Aphis 103 | ribosomal protein s2 | 968 | *Acyrthosiphon pisum* | 8.25E-154 | 5 | 2 |
| Aphis 111 | myosin light chain 2 | 1283 | *Acyrthosiphon pisum* | 1.41E-91 | 2 | 1 |
| Aphis 117 | acypi000079 | 453 | *Toxoptera citricida* | 2.55E-65 | 8 | 2 |
| Aphis 133 | ribosomal protein l10 | 762 | *Acyrthosiphon pisum* | 6.87E-159 | 3 | 2 |
| Aphis 200 | PREDICTED: hypothetical protein LOC100169357 isoform 1 | 1943 | *Acyrthosiphon pisum* | 4.44E-68 | 0 | 3 |
| Aphis 212 | muscle actin | 1670 | *Acyrthosiphon pisum* | 0 | 3 | 1 |
| Aphis 215 | elongation factor 1 alpha | 2548 | *Acyrthosiphon pisum* | 0 | 6 | 2 |
| Aphis 242 | elongation factor 2 | 2425 | *Toxoptera citricida* | 0 | 5 | 1 |
| Aphis 255 | zinc finger protein 512b-like | 917 | *Acyrthosiphon pisum* | 2.55E-49 | 0 | 11 |
| Aphis 273 | 40s ribosomal protein s8-like | 972 | *Hordeum vulgare* subsp*. vulgare* | 1.37E-131 | 3 | 4 |
| Aphis 309 | ribosomal protein s16 | 743 | *Acyrthosiphon pisum* | 2.57E-91 | 3 | 1 |
| Aphis 321 | h+ transporting atp synthase subunit g | 745 | *Acyrthosiphon pisum* | 1.46E-62 | 4 | 9 |
| Aphis 359 | malate cytoplasmic-like | 2642 | *Acyrthosiphon pisum* | 0 | 8 | 10 |
| Aphis 365 | cytochrome oxidase subunit i | 2550 | *Aphis nerii* | 6.23E-154 | 10 | 7 |
| Aphis 384 | N/A | 442 |  |  | 0 | 13 |
| Aphis 433 | ribosomal protein s13 | 811 | *Acyrthosiphon pisum* | 2.32E-101 | 4 | 2 |
| Aphis 480 | ribosomal protein l21 | 585 | *Acyrthosiphon pisum* | 2.50E-93 | 3 | 1 |
| Aphis 525 | 60s ribosomal protein l4-like | 1633 | *Hordeum vulgare* subsp*. vulgare* | 0 | 3 | 1 |
| Aphis 645 | cyclophilin 1 | 935 | *Acyrthosiphon pisum* | 1.44E-135 | 9 | 2 |
| Aphis 668 | ribosomal protein s7 | 723 | *Acyrthosiphon pisum* | 3.81E-136 | 3 | 1 |
| Aphis 704 | ribosomal protein l18a | 629 | *Acyrthosiphon pisum* | 1.55E-117 | 3 | 3 |
| Aphis 906 | PREDICTED: hypothetical protein LOC100570527 | 393 | *Acyrthosiphon pisum* | 1.92E-36 | 0 | 1 |
| Aphis 1221 | cg12324 protein | 831 | *Acyrthosiphon pisum* | 5.08E-87 | 5 | 1 |
| Aphis 1468 | isoform a | 495 | *Acyrthosiphon pisum* | 2.32E-36 | 0 | 1 |
| Aphis 1725 | tpa: cuticle protein | 746 | *Acyrthosiphon pisum* | 4.30E-59 | 1 | 1 |

**Table S3c.**

| **Contig ID** | **Sequence Description** | **Length (bp)** | **Organism** | **Minimum E-value** | **#GOs** | **#SNPs** |
| --- | --- | --- | --- | --- | --- | --- |
| Clavig 2 | hexamerin 1 | 1165 | *Riptortus clavatus* | 0 | 6 | 2 |
| Clavig 6 | N/A | 407 |  |  | 0 | 4 |
| Clavig 7 | serine rich protein | 389 | *Oncopeltus fasciatus* | 2.35E-10 | 0 | 6 |
| Clavig 9 | N/A | 439 |  |  | 0 | 6 |
| Clavig 17 | tropomyosin 1 | 1786 | *Lethocerus indicus* | 3.78E-158 | 0 | 1 |
| Clavig 18 | fk506-binding protein | 445 | *Daphnia pulex* | 1.12E-65 | 3 | 1 |
| Clavig 19 | fk506-binding protein | 412 | *Daphnia pulex* | 8.07E-48 | 3 | 2 |
| Clavig 20 | rrna intron-encoded homing endonuclease | 4211 | *Oxytricha trifallax* | 2.21E-55 | 1 | 2 |
| Clavig 26 | N/A | 717 |  |  | 0 | 1 |
| Clavig 37 | cathepsin l1 | 319 | *Ornithorhynchus anatinus* | 7.19E-43 | 4 | 2 |
| Clavig 39 | serine rich protein | 393 | *Oncopeltus fasciatus* | 1.50E-07 | 0 | 2 |
| Clavig 45 | cathepsin l | 310 | *Nematostella vectensis* | 1.19E-48 | 6 | 6 |
| Clavig 46 | ribosomal protein s6 | 808 | *Pediculus humanus corporis* | 4.56E-136 | 3 | 3 |
| Clavig 50 | apolipophorin-iii precursor | 464 | *Riptortus clavatus* | 4.79E-48 | 3 | 2 |
| Clavig 51 | h+ transporting atp synthase subunit d | 809 | *Papilio polytes* | 4.82E-71 | 10 | 3 |
| Clavig 53 | N/A | 437 |  |  | 0 | 1 |
| Clavig 66 | N/A | 491 |  |  | 0 | 2 |
| Clavig 67 | heat shock protein 90 | 1349 | *Lygus hesperus* | 4.57E-177 | 4 | 3 |
| Clavig 69 | N/A | 1089 |  |  | 0 | 8 |
| Clavig 70 | gluten hydrolyzing proteinase | 668 | *Triatoma brasiliensis* | 4.11E-35 | 3 | 1 |
| Clavig 75 | elongation factor 1-alpha | 1607 | *Riptortus pedestris* | 0 | 7 | 3 |
| Clavig 76 | elongation factor 1 alpha | 1434 | *Riptortus pedestris* | 0 | 7 | 2 |
| Clavig 81 | nadh dehydrogenase subunit 1 | 1531 | *Stictopleurus subviridis* | 8.91E-24 | 4 | 1 |
| Clavig 85 | ribosomal protein s4e | 890 | *Lygus lineolaris* | 1.66E-179 | 4 | 5 |
| Clavig 89 | lipoyltransferase mitochondrial-like | 2480 | *Bombus terrestris* | 9.43E-91 | 1 | 1 |
| Clavig 91 | odorant binding protein 24 | 619 | *Anopheles funestus* | 1.26E-07 | 3 | 1 |
| Clavig 103 | cathepsin l | 689 | *Aedes aegypti* | 3.99E-96 | 3 | 2 |
| Clavig 106 | cathepsin l | 497 | *Dermacentor variabilis* | 5.99E-68 | 3 | 9 |
| Clavig 107 | ankyrin repeat protein | 1315 | *Candidatus Amoebophilus asiaticus* 5a2 | 2.14E-60 | 3 | 5 |
| Clavig 111 | cathepsin l-like | 483 | *Apis florea* | 2.55E-60 | 3 | 1 |
| Clavig 115 | hexamerin 1 | 2153 | *Riptortus clavatus* | 0 | 6 | 2 |
| Clavig 116 | hexamerin 1 | 2098 | *Riptortus clavatus* | 0 | 6 | 2 |
| Clavig 126 | gluten hydrolyzing proteinase | 1035 | *Lygus lineolaris* | 1.61E-63 | 1 | 1 |
| Clavig 127 | N/A | 246 |  |  | 0 | 1 |
| Clavig 129 | cathepsin l | 948 | *Triatoma brasiliensis* | 3.59E-107 | 3 | 3 |
| Clavig 131 | transferrin | 2166 | *Riptortus clavatus* | 0 | 4 | 1 |
| Clavig 142 | N/A | 819 |  |  | 0 | 1 |
| Clavig 149 | N/A | 351 |  |  | 0 | 1 |
| Clavig 151 | N/A | 336 |  |  | 0 | 1 |
| Clavig 158 | N/A | 382 |  |  | 0 | 3 |
| Clavig 159 | lysozyme | 728 | *Anopheles gambiae* | 1.01E-46 | 2 | 1 |
| Clavig 166 | tubulin beta-1 chain | 1550 | *Manduca sexta* | 0 | 13 | 3 |
| Clavig 167 | N/A | 1319 |  |  | 0 | 5 |
| Clavig 168 | unknown | 1008 | *Lygus lineolaris* | 9.72E-13 | 0 | 4 |
| Clavig 172 | atp synthase subunit mitochondrial-like | 1949 | *Tribolium castaneum* | 0 | 9 | 3 |
| Clavig 174 | N/A | 1534 |  |  | 0 | 2 |
| Clavig 176 | heat shock protein 70 | 3830 | *Riftia pachyptila* | 0 | 37 | 1 |
| Clavig 177 | protein disulfide isomerase | 1026 | *Anopheles gambiae* str. PEST | 1.90E-138 | 14 | 2 |
| Clavig 178 | peritrophic matrix protein 1-b precursor | 430 | *Anopheles gambiae* str. PEST | 8.15E-08 | 3 | 5 |
| Clavig 180 | peritrophic matrix protein 1-b precursor | 384 | *Anopheles gambiae* str. PEST | 7.20E-08 | 3 | 1 |
| Clavig 187 | cuticle protein 34 | 426 | *Dendroctonus ponderosae* | 1.36E-24 | 1 | 5 |
| Clavig 191 | 60s ribosomal protein l4-like | 1360 | *Bombus impatiens* | 0 | 3 | 2 |
| Clavig 192 | probable maltase l-like | 665 | *Nilaparvata lugens* | 2.79E-26 | 2 | 2 |
| Clavig 196 | ribosomal protein l31 | 466 | *Acromyrmex echinatior* | 1.00E-53 | 3 | 1 |
| Clavig 201 | apolipophorins | 3607 | *Nilaparvata lugens* | 3.75E-121 | 14 | 2 |
| Clavig 206 | cg31997 cg31997-pa | 618 | *Megachile rotundata* | 8.50E-44 | 3 | 1 |
| Clavig 214 | myosin heavy muscle isoform 1 | 6145 | *Acyrthosiphon pisum* | 0 | 25 | 8 |
| Clavig 215 | ribosomal protein l13 | 760 | *Xenopsylla cheopis* | 1.68E-84 | 3 | 1 |
| Clavig 227 | N/A | 493 |  |  | 0 | 5 |
| Clavig 238 | N/A | 613 |  |  | 0 | 1 |
| Clavig 239 | alpha-amylase | 1615 | *Blattella germanica* | 3.91E-178 | 3 | 9 |
| Clavig 242 | maltase a3 | 775 | *Nilaparvata lugens* | 2.63E-41 | 3 | 1 |
| Clavig 243 | N/A | 1804 |  |  | 0 | 2 |
| Clavig 247 | N/A | 301 |  |  | 0 | 1 |
| Clavig 251 | serine rich protein | 488 | *Oncopeltus fasciatus* | 4.72E-19 | 0 | 4 |
| Clavig 256 | atp-citrate synthase-like | 3916 | *Acyrthosiphon pisum* | 0 | 10 | 5 |
| Clavig 261 | ribosomal protein l14 | 562 | *Lygus lineolaris* | 5.10E-68 | 3 | 1 |
| Clavig 266 | ribosomal protein l15 | 739 | *Pediculus humanus corporis* | 6.99E-118 | 3 | 1 |
| Clavig 284 | fatty acid synthase | 3168 | *Nasonia vitripennis* | 0 | 5 | 1 |
| Clavig 293 | serine rich protein | 1438 | *Oncopeltus fasciatus* | 8.70E-24 | 0 | 2 |
| Clavig 296 | alpha amylase catalytic region | 439 | *Fusarium oxysporum* Fo5176 | 2.59E-45 | 3 | 12 |
| Clavig 297 | alpha partial | 492 | *Acromyrmex echinatior* | 1.73E-42 | 3 | 1 |
| Clavig 298 | probable maltase h-like | 577 | *Tribolium castaneum* | 1.99E-71 | 3 | 5 |
| Clavig 300 | maltase 1 | 2668 | *Nilaparvata lugens* | 6.02E-144 | 3 | 2 |
| Clavig 301 | N/A | 335 |  |  | 0 | 8 |
| Clavig 318 | N/A | 719 |  |  | 0 | 3 |
| Clavig 320 | ribosomal protein l23a | 1016 | *Tribolium castaneum* | 2.30E-82 | 7 | 2 |
| Clavig 322 | ribosomal protein l7 | 894 | *Papilio polytes* | 3.66E-127 | 1 | 3 |
| Clavig 332 | N/A | 511 |  |  | 0 | 7 |
| Clavig 345 | cytochrome c | 647 | *Graphocephala atropunctata* | 1.65E-62 | 5 | 1 |
| Clavig 349 | atp synthase-coupling factor mitochondrial | 482 | *Tribolium castaneum* | 3.73E-36 | 10 | 2 |
| Clavig 356 | 60s acidic ribosomal protein p2-like protein | 478 | *Bombyx mori* | 3.88E-25 | 4 | 1 |
| Clavig 366 | probable phosphoserine aminotransferase-like | 1161 | *Harpegnathos saltator* | 3.28E-180 | 4 | 1 |
| Clavig 372 | conserved hypothetical protein | 432 | *Culex quinquefasciatus* | 4.59E-13 | 3 | 1 |
| Clavig 378 | N/A | 334 |  |  | 0 | 3 |
| Clavig 384 | cathepsin l-like | 656 | *Artemia salina* | 1.29E-80 | 3 | 1 |
| Clavig 386 | N/A | 988 |  |  | 0 | 4 |
| Clavig 398 | N/A | 849 |  |  | 0 | 2 |
| Clavig 399 | accessory gland protein | 1512 | *Gryllus firmus* | 5.08E-10 | 0 | 2 |
| Clavig 406 | abp2_ripcl ame: full=probable antibacterial peptide flags: precursor | 444 | *Riptortus clavatus* | 2.64E-34 | 3 | 4 |
| Clavig 408 | N/A | 1245 |  |  | 0 | 2 |
| Clavig 410 | cytochrome c oxidase subunit iii | 2075 | *Hydaropsis longirostris* | 1.12E-128 | 5 | 2 |
| Clavig 426 | aminopeptidase n-like | 3964 | *Strongylocentrotus purpuratus* | 7.23E-39 | 4 | 1 |
| Clavig 433 | 50 kda midgut protein | 737 | *Nasonia vitripennis* | 1.24E-20 | 0 | 1 |
| Clavig 456 | AGAP004851-PA | 376 | *Anopheles gambiae* str. PEST | 3.62E-10 | 3 | 2 |
| Clavig 466 | heat shock protein 70 | 2783 | *Culex quinquefasciatus* | 0 | 6 | 9 |
| Clavig 475 | vitellogenin | 435 | *Riptortus clavatus* | 1.59E-44 | 6 | 1 |
| Clavig 484 | N/A | 365 |  |  | 0 | 2 |
| Clavig 487 | cathepsin l-like | 432 | *Camponotus floridanus* | 9.24E-58 | 3 | 1 |
| Clavig 490 | N/A | 424 |  |  | 0 | 3 |
| Clavig 497 | N/A | 596 |  |  | 0 | 2 |
| Clavig 501 | vitellogenin | 3404 | *Riptortus clavatus* | 0 | 2 | 8 |
| Clavig 509 | odorant-binding protein partial | 371 | *Rhodnius prolixus* | 3.25E-36 | 1 | 2 |
| Clavig 513 | cytochrome b | 625 | *Hydaropsis longirostris* | 2.71E-96 | 6 | 1 |
| Clavig 518 | cytochrome b | 626 | *Hydaropsis longirostris* | 1.43E-92 | 6 | 2 |
| Clavig 524 | odorant-binding protein partial | 586 | *Adelphocoris lineolatus* | 4.22E-14 | 1 | 3 |
| Clavig 533 | actin | 1748 | *Drosophila melanogaster* | 0 | 15 | 5 |
| Clavig 544 | odorant binding protein 19d | 698 | *Apolygus lucorum* | 1.17E-15 | 1 | 4 |
| Clavig 564 | N/A | 377 |  |  | 1 | 1 |
| Clavig 576 | peripheral-type benzodiazepine receptor | 1114 | *Drosophila pseudoobscura pseudoobscura* | 3.85E-36 | 4 | 1 |
| Clavig 587 | isoform a | 597 | *Drosophila willistoni* | 7.77E-18 | 2 | 1 |
| Clavig 590 | N/A | 406 |  |  | 0 | 5 |
| Clavig 591 | hypothetical protein DAPPUDRAFT_70492 | 597 | *Daphnia pulex* | 2.39E-15 | 1 | 1 |
| Clavig 605 | N/A | 761 |  |  | 0 | 1 |
| Clavig 606 | af373879_1peritrophin-like protein 1 | 1921 | *Anopheles gambiae* str. PEST | 1.53E-12 | 3 | 1 |
| Clavig 607 | serine rich protein | 417 | *Oncopeltus fasciatus* | 4.33E-20 | 0 | 23 |
| Clavig 614 | ornithine decarboxylase | 1037 | *Pediculus humanus corporis* | 8.89E-44 | 2 | 2 |
| Clavig 615 | hypothetical protein AaeL_AAEL015254 | 1063 | *Aedes aegypti* | 6.46E-12 | 3 | 2 |
| Clavig 625 | nadh dehydrogenase subunit i | 827 | *Aeschyntelus notatus* | 2.17E-78 | 4 | 3 |
| Clavig 626 | fructose -bisphosphate aldolase | 1457 | *Daphnia pulex* | 1.97E-157 | 19 | 11 |
| Clavig 640 | gamma-interferon-inducible lysosomal thiol reductase-like | 1580 | *Maconellicoccus hirsutus* | 3.11E-23 | 0 | 1 |
| Clavig 643 | acetyl- mitochondrial | 1139 | *Acyrthosiphon pisum* | 0 | 14 | 4 |
| Clavig 670 | gluten hydrolyzing proteinase | 617 | *Triatoma brasiliensis* | 2.16E-37 | 3 | 2 |
| Clavig 686 | ankyrin repeat protein | 1050 | *Oncopeltus fasciatus* | 2.88E-49 | 0 | 5 |
| Clavig 692 | beta-tubulin | 1737 | *Manduca sexta* | 0 | 13 | 5 |
| Clavig 711 | nucleoside diphosphate kinase | 787 | *Aedes aegypti* | 1.19E-80 | 15 | 1 |
| Clavig 716 | elongation factor 1-gamma | 1340 | *Maconellicoccus hirsutus* | 2.52E-137 | 13 | 1 |
| Clavig 726 | N/A | 523 |  |  | 0 | 3 |
| Clavig 745 | N/A | 304 |  |  | 0 | 1 |
| Clavig 757 | N/A | 521 |  |  | 0 | 1 |
| Clavig 765 | ankyrin repeat protein | 1330 | *Trichomonas vaginalis* G3 | 1.41E-39 | 1 | 1 |
| Clavig 768 | counting factor associated protein d-like | 1722 | *Periplaneta americana* | 0 | 6 | 4 |
| Clavig 775 | apolipoprotein d | 1114 | *Pediculus humanus corporis* | 4.88E-85 | 1 | 2 |
| Clavig 784 | N/A | 645 |  |  | 0 | 1 |
| Clavig 799 | N/A | 654 |  |  | 0 | 2 |
| Clavig 817 | serine rich protein | 1196 | *Oncopeltus fasciatus* | 4.24E-19 | 0 | 1 |
| Clavig 858 | ribosomal protein l17 | 596 | *Pediculus humanus corporis* | 3.37E-97 | 3 | 1 |
| Clavig 861 | ankyrin repeat protein | 913 | *Diplorickettsia massiliensis* 20B | 1.06E-17 | 1 | 2 |
| Clavig 869 | endocuticle structural glycoprotein bd-1 | 2627 | *Anopheles gambiae* str. PEST | 8.82E-25 | 1 | 1 |
| Clavig 891 | phosphoenolpyruvate carboxykinase | 2001 | *Tribolium castaneum* | 0 | 5 | 1 |
| Clavig 936 | spike protein | 4244 | *Hana virus* | 5.94E-15 | 0 | 8 |
| Clavig 939 | N/A | 1018 |  |  | 0 | 1 |
| Clavig 945 | heat shock 70 kda protein cognate 3 | 2376 | *Nasonia vitripennis* | 0 | 6 | 1 |
| Clavig 953 | N/A | 746 |  |  | 0 | 3 |
| Clavig 954 | N/A | 448 |  |  | 0 | 2 |
| Clavig 959 | ribosomal protein l10 | 298 | *Acyrthosiphon pisum* | 8.66E-48 | 3 | 1 |
| Clavig 960 | ribosomal protein l10 | 396 | *Acyrthosiphon pisum* | 1.03E-89 | 3 | 2 |
| Clavig 964 | N/A | 972 |  |  | 0 | 3 |
| Clavig 965 | mitochondrial atp synthase gamma-subunit | 808 | *Graphocephala atropunctata* | 8.95E-142 | 8 | 1 |
| Clavig 984 | mitochondrial-processing peptidase subunit beta-like | 1569 | *Anopheles gambiae* str. PEST | 0 | 12 | 1 |
| Clavig 992 | polyadenylate-binding protein 1-like isoform 1 | 3350 | *Bombus terrestris* | 0 | 0 | 1 |
| Clavig 993 | N/A | 1126 |  |  | 0 | 10 |
| Clavig 996 | thiamin pyrophosphokinase 1 | 1356 | *Tribolium castaneum* | 1.65E-56 | 4 | 3 |
| Clavig 1003 | ribosomal protein l22 | 748 | *Danaus plexippus* | 1.54E-30 | 3 | 1 |
| Clavig 1007 | mdl1 | 592 | *Acromyrmex echinatior* | 3.38E-33 | 0 | 1 |
| Clavig 1029 | malic enzyme | 480 | *Pediculus humanus corporis* | 3.40E-29 | 4 | 2 |
| Clavig 1038 | phosphate carrier mitochondrial-like | 1349 | *Aedes aegypti* | 1.98E-158 | 2 | 1 |
| Clavig 1049 | fatty acid desaturase | 1866 | *Acheta domesticus* | 3.52E-164 | 15 | 2 |
| Clavig 1052 | N/A | 1094 |  |  | 0 | 4 |
| Clavig 1054 | ribosomal protein l26e | 456 | *Triatoma infestans* | 1.10E-80 | 3 | 1 |
| Clavig 1055 | isoform c | 1333 | *Drosophila ananassae* | 4.28E-25 | 3 | 8 |
| Clavig 1058 | hexamerin 1 | 576 | *Riptortus clavatus* | 1.36E-99 | 6 | 1 |
| Clavig 1075 | mitochondrial cytochrome c oxidase subunit 5b isoform 1 | 565 | *Tribolium castaneum* | 2.35E-53 | 6 | 3 |
| Clavig 1079 | serine protease | 613 | *Triatoma infestans* | 5.38E-32 | 3 | 8 |
| Clavig 1087 | ribosomal protein s9 | 665 | *Graphocephala atropunctata* | 6.36E-123 | 4 | 1 |
| Clavig 1088 | ribosomal protein s9 | 641 | *Graphocephala atropunctata* | 4.65E-123 | 4 | 2 |
| Clavig 1125 | chitin binding peritrophin-a domain-containing partial | 1714 | *Drosophila ananassae* | 5.52E-45 | 3 | 2 |
| Clavig 1126 | mitochondrial cytochrome c oxidase subunit 5b isoform 1 | 533 | *Triatoma infestans* | 7.91E-51 | 6 | 1 |
| Clavig 1167 | nucleoplasmin-like protein | 1088 | *Maconellicoccus hirsutus* | 1.64E-55 | 1 | 1 |
| Clavig 1182 | N/A | 217 |  |  | 0 | 1 |
| Clavig 1187 | N/A | 369 |  |  | 0 | 1 |
| Clavig 1213 | N/A | 568 |  |  | 0 | 8 |
| Clavig 1243 | 60s ribosomal protein l7a | 840 | *Solenopsis invicta* | 1.82E-136 | 2 | 1 |
| Clavig 1251 | aminopeptidase -like | 957 | *Solenopsis invicta* | 1.91E-105 | 5 | 1 |
| Clavig 1261 | N/A | 850 |  |  | 0 | 2 |
| Clavig 1280 | N/A | 976 |  |  | 0 | 1 |
| Clavig 1286 | 40s ribosomal protein s16 | 591 | *Apis florea* | 3.29E-91 | 3 | 1 |
| Clavig 1292 | cathepsin l precursor | 365 | *Triatoma brasiliensis* | 1.08E-18 | 1 | 1 |
| Clavig 1319 | ribosomal protein s12 | 422 | *Manduca sexta* | 5.41E-61 | 3 | 1 |
| Clavig 1321 | ribosomal protein l27e | 514 | *Hister sp.* APV-2005 | 1.61E-54 | 3 | 1 |
| Clavig 1347 | ribosomal protein l13a | 650 | *Bombus terrestris* | 2.78E-113 | 3 | 1 |
| Clavig 1350 | imaginal disc growth factor | 1070 | *Oncometopia nigricans* | 1.76E-133 | 3 | 2 |
| Clavig 1364 | 15-hydroxyprostaglandin dehydrogenase | 1045 | *Acromyrmex echinatior* | 1.04E-53 | 2 | 5 |
| Clavig 1401 | atp synthase subunit mitochondrial | 2063 | *Megachile rotundata* | 0 | 9 | 4 |
| Clavig 1418 | cg12324 protein | 468 | *Triatoma infestans* | 2.56E-84 | 7 | 1 |
| Clavig 1429 | N/A | 899 |  |  | 0 | 6 |
| Clavig 1431 | kininogen-1 isoform 2 precursor | 635 | *Oncopeltus fasciatus* | 2.74E-19 | 4 | 6 |
| Clavig 1501 | odorant-binding protein | 972 | *Apolygus lucorum* | 5.66E-16 | 1 | 1 |
| Clavig 1509 | nadh dehydrogenase subunit 5 | 1560 | *Hydaropsis longirostris* | 3.27E-160 | 4 | 1 |
| Clavig 1522 | atp-dependent rna helicase-like protein | 797 | *Trypanosoma brucei gambiense* DAL972 | 1.78E-22 | 4 | 3 |
| Clavig 1573 | guanine nucleotide-binding protein subunit beta-like | 1073 | *Blattella germanica* | 0 | 2 | 1 |
| Clavig 1605 | N/A | 358 |  |  | 0 | 2 |
| Clavig 1606 | N/A | 565 |  |  | 0 | 1 |
| Clavig 1626 | N/A | 581 |  |  | 0 | 1 |
| Clavig 1697 | nadh dehydrogenase subunit 4 | 455 | *Riptortus pedestris* | 3.18E-34 | 4 | 1 |
| Clavig 1708 | p8 nuclear protein | 860 | *Amblyomma variegatum* | 1.83E-26 | 2 | 1 |
| Clavig 1724 | N/A | 396 |  |  | 0 | 2 |
| Clavig 1772 | calmodulin | 1707 | *Drosophila melanogaster* | 3.64E-96 | 35 | 1 |
| Clavig 1782 | hypothetical protein | 558 | *Triatoma brasiliensis* | 2.02E-12 | 0 | 1 |
| Clavig 1785 | midline fasciclin | 1385 | *Tribolium castaneum* | 3.08E-58 | 1 | 3 |
| Clavig 1842 | af414430_1trypsin precursor | 601 | *Lygus lineolaris* | 4.91E-48 | 3 | 1 |
| Clavig 1849 | cathepsin l | 1503 | *Triatoma brasiliensis* | 9.22E-133 | 3 | 5 |
| Clavig 1913 | lyzozyme m1 | 832 | Wolbachia endosymbiont of *Drosophila ananassae* | 2.41E-76 | 2 | 5 |
| Clavig 1979 | translocon-associated protein subunit beta | 1392 | *Maconellicoccus hirsutus* | 5.91E-68 | 9 | 1 |
| Clavig 1983 | endocuticle structural glycoprotein bd- | 637 | *Acyrthosiphon pisum* | 8.20E-22 | 1 | 1 |
| Clavig 2008 | cathepsin l | 2153 | *Harpegnathos saltator* | 1.27E-109 | 3 | 1 |
| Clavig 2035 | serine protease | 674 | *Ranatra unicolor* | 7.66E-45 | 3 | 2 |
| Clavig 2045 | ribosomal protein l30 | 426 | *Daphnia pulex* | 1.52E-63 | 3 | 1 |
| Clavig 2054 | 15-hydroxyprostaglandin dehydrogenase | 1011 | *Acromyrmex echinatior* | 1.69E-43 | 2 | 13 |
| Clavig 2077 | cathepsin d | 1339 | *Triatoma infestans* | 8.43E-100 | 3 | 1 |
| Clavig 2094 | 60s ribosomal protein l18 | 627 | *Camponotus floridanus* | 2.24E-103 | 3 | 2 |
| Clavig 2104 | sorbitol dehydrogenase | 1123 | *Pyrrhocoris apterus* | 0 | 4 | 1 |
| Clavig 2132 | N/A | 262 |  |  | 0 | 11 |
| Clavig 2209 | N/A | 338 |  |  | 0 | 1 |
| Clavig 2326 | elongation factor 1 delta | 825 | *Graphocephala atropunctata* | 9.39E-61 | 8 | 4 |
| Clavig 2422 | salivary secreted peptide | 491 | *Lygus lineolaris* | 2.08E-15 | 0 | 3 |
| Clavig 2438 | serine 3-dehydrogenase | 861 | *Triatoma infestans* | 2.43E-47 | 2 | 3 |
| Clavig 2677 | cytochrome c oxidase polypeptide iv | 624 | *Locusta migratoria* | 2.37E-71 | 8 | 1 |
| Clavig 2823 | 10 kda heat shock mitochondrial-like | 819 | *Lygus hesperus* | 7.81E-55 | 4 | 3 |
| Clavig 2828 | ubiquinol-cytochrome c reductase complex core protein | 1515 | *Tribolium castaneum* | 6.13E-98 | 6 | 1 |
| Clavig 2851 | N/A | 583 |  |  | 0 | 2 |
| Clavig 2856 | protein 5nuc-like | 2073 | *Camponotus floridanus* | 5.06E-121 | 3 | 1 |
| Clavig 2888 | PREDICTED: uncharacterized protein LOC100863228 | 1252 | *Apis florea* | 1.48E-37 | 0 | 7 |
| Clavig 3671 | voltage-dependent anion-selective channel protein 2 | 557 | *Homalodisca vitripennis* | 1.70E-78 | 7 | 1 |
| Clavig 4404 | v-type proton atpase subunit g-like | 475 | *Acyrthosiphon pisum* | 2.19E-27 | 5 | 2 |

**Table S3d.**

| **Contig ID** | **Sequence Description** | **Length (bp)** | **Organism** | **Minimum E-value** | **#GOs** | **#SNPs** |
| --- | --- | --- | --- | --- | --- | --- |
| Megal 1 | elongation factor 2 | 1780 | *Schistocerca gregaria* | 0 | 6 | 1 |
| Megal 11 | N/A | 1006 |  |  | 2 | 9 |
| Megal 13 | vitellogenin | 4590 | *Trigonotylus caelestialium* | 0 | 2 | 31 |
| Megal 25 | storage protein 1 | 2148 | *Chilo suppressalis* | 0 | 3 | 5 |
| Megal 34 | vitellogenin | 2855 | *Trigonotylus caelestialium* | 0 | 2 | 2 |
| Megal 47 | arylphorin-type storage protein | 462 | *Omphisa fuscidentalis* | 2.77E-71 | 3 | 1 |
| Megal 49 | mitochondrial aldehyde dehydrogenase | 624 | *Danaus plexippus* | 8.33E-107 | 4 | 3 |
| Megal 68 | ribosomal protein l27ae | 517 | *Camponotus floridanus* | 5.94E-64 | 3 | 3 |
| Megal 72 | ribosomal protein l4 | 1323 | *Biphyllus lunatus* | 0 | 3 | 2 |
| Megal 76 | 60s ribosomal protein l5-like | 1257 | *Helianthus annuus* | 6.70E-165 | 5 | 1 |
| Megal 85 | arylphorin precursor | 1485 | *Omphisa fuscidentalis* | 0 | 1 | 2 |
| Megal 92 | ribosomal protein s12 | 543 | *Apis florea* | 2.42E-74 | 3 | 2 |
| Megal 107 | vitellogenin | 379 | *Lethocerus deyrollei* | 7.45E-33 | 4 | 2 |
| Megal 143 | N/A | 423 |  |  | 0 | 3 |
| Megal 157 | N/A | 987 |  |  | 0 | 1 |
| Megal 183 | actin | 1612 | *Ornithodoros moubata* | 0 | 13 | 1 |
| Megal 191 | heat shock protein 90 | 2413 | *Megachile rotundata* | 0 | 4 | 4 |
| Megal 193 | 40s ribosomal protein s15 | 545 | *Diaphorina citri* | 1.09E-81 | 6 | 1 |
| Megal 199 | mitochondrial atp synthase coupling factor 6 | 537 | *Tribolium castaneum* | 4.06E-25 | 5 | 2 |
| Megal 202 | N/A | 1411 |  |  | 0 | 4 |
| Megal 215 | 60s ribosomal protein l12 | 865 | *Drosophila ananassae* | 2.02E-98 | 5 | 1 |
| Megal 236 | hexamerin 2 beta | 2534 | *Helicoverpa armigera* | 0 | 3 | 10 |
| Megal 238 | vitellogenin | 974 | *Lethocerus deyrollei* | 4.17E-80 | 4 | 7 |
| Megal 312 | ribosomal protein s7 | 650 | *Carabus granulatus* | 2.00E-113 | 3 | 2 |
| Megal 346 | cuticle protein 1 | 349 | *Lonomia obliqua* | 1.10E-35 | 1 | 3 |
| Megal 360 | 40s ribosomal protein s14 | 569 | *Dascillus cervinus* | 8.78E-73 | 4 | 2 |
| Megal 362 | cytochrome c oxidase subunit iii | 398 | *Frankliniella intonsa* | 1.84E-41 | 4 | 1 |
| Megal 370 | heat shock protein 70 | 1555 | *Frankliniella occidentalis* | 0 | 3 | 2 |
| Megal 376 | troponin i | 871 | *Loxostege sticticalis* | 1.48E-85 | 1 | 1 |
| Megal 378 | ribosomal protein s8 | 713 | *Megachile rotundata* | 1.45E-124 | 3 | 1 |
| Megal 408 | N/A | 216 |  |  | 0 | 1 |
| Megal 419 | ribosomal protein l35 | 512 | *Chrysomela tremula* | 1.03E-50 | 3 | 1 |
| Megal 471 | atp synthase-like protein | 1067 | *Culex quinquefasciatus* | 9.70E-64 | 2 | 1 |
| Megal 475 | actin | 688 | *Diaphorina citri* | 1.76E-159 | 3 | 1 |
| Megal 491 | ribosomal protein s3 | 781 | *Scarabaeus laticollis* | 4.38E-156 | 16 | 2 |
| Megal 533 | N/A | 662 |  |  | 1 | 2 |
| Megal 537 | partial | 721 | *Trigonotylus caelestialium* | 3.39E-103 | 2 | 3 |
| Megal 551 | ribosomal protein s28 | 408 | *Biphyllus lunatus* | 1.09E-27 | 4 | 1 |
| Megal 569 | N/A | 806 |  |  | 1 | 2 |
| Megal 572 | N/A | 475 |  |  | 0 | 2 |
| Megal 591 | tubulin alpha-1 chain | 1518 | *Pediculus humanus corporis* | 0 | 12 | 1 |
| Megal 603 | ribosomal protein s18 | 523 | *Cicindela campestris* | 2.71E-74 | 4 | 1 |
| Megal 608 | N/A | 533 |  |  | 0 | 4 |
| Megal 624 | 60s ribosomal protein l23a-like | 716 | *Tribolium castaneum* | 3.42E-73 | 7 | 2 |
| Megal 656 | endocuticle structural glycoprotein bd-8-like | 784 | *Papilio xuthus* | 1.42E-28 | 1 | 1 |
| Megal 675 | ribosomal protein l34 | 437 | *Spodoptera frugiperda* | 1.04E-74 | 3 | 3 |
| Megal 716 | cytochrome oxidase subunit viic | 447 | *Ixodes pacificus* | 4.41E-09 | 1 | 1 |
| Megal 749 | apolipoprotein d-like | 672 | *Aedes aegypti* | 9.06E-39 | 6 | 1 |
| Megal 829 | tpa: cuticle protein | 374 | *Papilio xuthus* | 1.95E-17 | 1 | 2 |
| Megal 836 | female neotenic-specific protein 3 | 1303 | *Trigonotylus caelestialium* | 1.58E-11 | 2 | 1 |
| Megal 861 | glutathione s-transferase | 633 | *Choristoneura fumiferana* | 2.54E-100 | 2 | 6 |
| Megal 909 | alo2_acrlo ame: full=antimicrobial peptide alo-2 | 338 | *Acrocinus longimanus* | 1.42E-14 | 3 | 9 |
| Megal 952 | ribosomal protein l21 | 534 | *Euphydryas aurinia* | 9.58E-81 | 3 | 1 |
| Megal 1068 | N/A | 2803 |  |  | 0 | 1 |
| Megal 1087 | histone h2a | 809 | *Crassostrea gigas* | 1.18E-75 | 5 | 1 |
| Megal 1114 | N/A | 723 |  |  | 4 | 2 |
| Megal 1118 | pyruvate dehydrogenase | 1656 | *Acromyrmex echinatior* | 0 | 4 | 2 |
| Megal 1263 | trypsin-like serine protease | 676 | *Ostrinia nubilalis* | 1.55E-101 | 3 | 4 |
| Megal 1348 | elongation factor-1alpha partial | 366 | *Blasticotoma filiceti* | 3.34E-79 | 5 | 1 |
| Megal 1482 | ribosomal protein s11 | 492 | *Bombyx mori* | 1.03E-92 | 3 | 2 |
| Megal 1626 | 60s acidic ribosomal protein p0 | 650 | *Blaptica dubia* | 3.76E-135 | 5 | 1 |
| Megal 1634 | odorant-binding protein | 638 | *Danaus plexippus* | 6.46E-44 | 1 | 6 |
| Megal 1739 | translocon-associated protein subunit delta | 718 | *Acromyrmex echinatior* | 2.53E-52 | 2 | 1 |
